# Multi‐tissue Metabolic GWAS and Drought‐Responsive Multi‐omics Reveal the Genetic Basis of the Quinoa Metabolome

**DOI:** 10.1002/advs.76426

**Published:** 2026-07-07

**Authors:** Julia von Steimker, Elodie L. Rey, Clara Stanschewski, Regina Wendenburg, Annabella Klemmer, Markéta Macho, Venkatesh P. Thirumalaikumar, Noha O. Saber, Aleksandra Skirycz, Alisdair R. Fernie, Mark Tester, Saleh Alseekh

**Affiliations:** ^1^ Max‐Planck‐Institute of Molecular Plant Physiology Potsdam‐Golm Germany; ^2^ Plant Science Program Biological and Environmental Science and Engineering Division King Abdullah University of Science and Technology (KAUST) Thuwal Saudi Arabia; ^3^ Center for Desert Agriculture KAUST Thuwal Saudi Arabia; ^4^ Crop Genetics Team BASF Belgium Coordination Center CommV Gent Zwijnaarde Belgium; ^5^ Center for Plant Biology Purdue University West Lafayette Indiana USA; ^6^ College of Natural Science Department of Biochemistry & Molecular Biology Michigan State University East Lansing Michigan USA; ^7^ Center of Plant Systems Biology and Biotechnology Plovdiv Bulgaria

**Keywords:** *Chenopodium quinoa*, drought stress, genome‐wide association study, metabolomic profiling, proteomic profiling, transcriptome profiling

## Abstract

Quinoa (*Chenopodium quinoa*) is a nutrient‐rich pseudocereal with diverse specialized metabolites, yet the genetic basis of this metabolic diversity is poorly understood. Here we integrate whole‐genome sequencing and multi‐tissue metabolic profiling of 603 quinoa accessions. We detected 4688 metabolic features and identified over 1000 metabolites in seeds, leaves, and roots. Using multi‐tissue genome‐wide association, we mapped the genetic architecture of quinoa metabolome by identifying 584 quantitative trait loci (QTL) and prioritized 219 candidate genes across 58 major QTL governing saponin, betalain, and flavonoid biosynthesis. Moreover, we constructed a drought‐responsive multi‐omics regulatory network and uncovered additional key genes involved in quinoa stress signaling and metabolic pathways. Finally, we cloned and functional validated the roles of cytochrome P450 76AD1 (CYP76AD1) in betalamate accumulation, UDP‐glycosyltransferase (UGT91C1) in flavonoid glycosylation, and CYP72A154 and soyasapogenol B glucuronide galactosyltransferase in saponin biosynthesis. This multi‐omic framework provides a high‐resolution map of the quinoa metabolome and a foundation for breeding nutrient‐rich and stress‐resilient quinoa cultivars.

## Graphical abstract

A multi‐omics framework combining multitissue genome‐wide association studies, metabolomics, transcriptomics, proteomics, and functional validation uncovers the genetic basis of specialized metabolism in quinoa. The study identifies hundreds of metabolite‐associated loci, prioritizes candidate genes for saponin, betalain, and flavonoid biosynthesis, and links metabolic diversity with drought‐responsive regulatory networks.

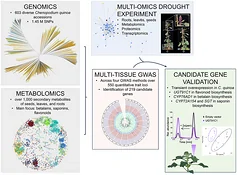

## 1 Introduction

Quinoa (*Chenopodium quinoa* Willd.) is an allotetraploid crop (2*n* = 4*x* = 36) that originated in the Andean region of South America approximately 7000 years ago [1, 2]. It is emerging as a highly nutritious food, with seeds rich in gluten free protein and essential amino acids [3, 4]. Quinoa is adaptable to a wide range of agricultural systems due to its remarkable tolerance to multiple abiotic stresses, including salinity [5, 6], drought [7], frost [8, 9], and high ultraviolet radiation [10], rendering it a promising crop for ensuring food security under climate change.

Recent advances in quinoa research have led to the development of extensive genetic and genomic resources aimed at improving agronomic performance and stress resilience [1, 11, 12, 13, 14]. These efforts have enabled the application of genome‐wide association studies (GWAS) to uncover the genetic architecture of traits such as seed weight, mildew resistance [15], salinity tolerance [14], flowering time [15], and seed color [16].

The plant metabolome, characterized by vast chemical diversity, plays critical roles in development, environmental adaptation, and defense responses [17, 18, 19]. Many plant‐derived metabolites, such as vitamins, flavonoids, phytosterols, phenolics, and carotenoids, also contribute significantly to human health as nutritional and medicinal compounds [20, 21, 22]. Over the past decades, integrative omics approaches – including metabolomics, genomics, and transcriptomics – have proven effective in elucidating gene function and metabolic pathways [23, 24]. Exploring metabolite diversity and its underlying genetic variation offers valuable insights for crop improvement, germplasm conservation, and the understanding of domestication [22, 25, 26].

GWAS and metabolome quantitative trait locus (mQTL) mapping have been successfully employed in numerous crops to reveal the genetic basis of metabolic variation [27] and to identify loci associated with quality traits and environmental responses [28]. As a result, biosynthetic and regulatory genes for key metabolite classes – such as flavonoids in rice [29, 30] and maize [31], steroidal glycoalkaloids in tomato [32], and saponins [1, 33, 34] – have been identified and functionally characterized.

Despite recent progress in quinoa genomics and phenotypic characterization, the genetic basis of metabolic diversity in quinoa remains largely unexplored. Notably, quinoa metabolites such as saponins, betalains, and flavonoids contribute to both seed quality – impacting flavor, nutrition, and processing – and plant stress tolerance [35, 36, 37, 38]. For example, betalains, exclusive to the Caryophyllales order, are potent antioxidants that influence seed coloration and protect against drought, UV, and salinity [39]. Their biosynthesis originates from l‐tyrosine through pathways involving cytochrome P450‐mediated reactions, yielding betalamic acid and *cyclo*‐DOPA or through dopamine by decarboxylation, generating a highly structurally diverse compound class by spontaneous conjugation and condensation reactions [39, 40].

Saponins are abundant in quinoa seed coats, contributing to bitterness and requiring removal prior to consumption [41, 42]. Nonetheless, they play protective roles against pathogens and pests [43] and some have recognized health benefits [44]. Their biosynthesis involves squalene epoxidation, followed by complex cyclization, oxidation, and glycosylation steps, which generate a diverse spectrum of saponins through these secondary modifications [45, 46, 47]. Notably, quinoa exhibits remarkable structural diversity of triterpenoid saponins compared to many related species, with nearly 100 distinct saponins described across different quinoa genotypes [1, 48, 49]. This diversification likely reflects both conserved Caryophyllales biosynthetic pathways and species‐specific expansion of regulatory and metabolic gene families involved in specialized metabolism. Saponin content is also thought to differentiate bitter and sweet quinoa varieties and is largely regulated by transcription factors such as TSARL1 and TSARL2 [1, 33, 34], although recent studies suggest flavonoids and polyphenols as additional main bitterness driver [50]. Similarly, flavonoids are a major class of metabolites with diverse physiological functions in plants and implications for human health [51, 52].

To date, most metabolomic studies in quinoa have examined a limited number of accessions, and no large‐scale metabolic GWAS (mGWAS) has been conducted. Understanding the genetic basis of quinoa's metabolome is therefore critical for leveraging natural variation in key functional metabolites. In this study, we integrated whole‐genome sequencing with untargeted liquid chromatography–mass spectrometry (LC–MS)‐based metabolic profiling across three tissues – seeds, leaves, and roots – of 603 quinoa accessions. Using mGWAS, we dissected the genetic architecture of metabolite accumulation, with an emphasis on saponins, flavonoids, and betalains in a tissue‐specific manner. Furthermore, through a multi‐omics approach incorporating transcriptomics, proteomics, and metabolomics under drought stress, we identified key molecular components involved in stress adaptation. Our findings provide a foundational resource for dissecting metabolic regulation in quinoa and offer promising targets for breeding nutritionally superior and stress‐resilient cultivars.

## 2 Results

### 2.1 The Genomic Diversity of the Quinoa Diversity Panel

Previously we analyzed a quinoa core collection of 310 accessions, reporting 2.9 million polymorphic single‐nucleotide variants [15]. Here, we made use of and characterized a quinoa diversity panel comprising 603 *C. quinoa* accessions originating primarily from Argentina, Bolivia, Chile, Peru, and the United States, spanning elevations from 12 to 4899 meters above sea level (Figure 1A). Whole‐genome sequencing yielded 13.7 million single‐nucleotide polymorphisms (SNPs), of which 1.45 million high‐confidence variants were retained after stringent quality control (60 417–104 938 SNPs per chromosome; transition/transversion ratio = 1.53; Figure S1A).

**FIGURE 1:**
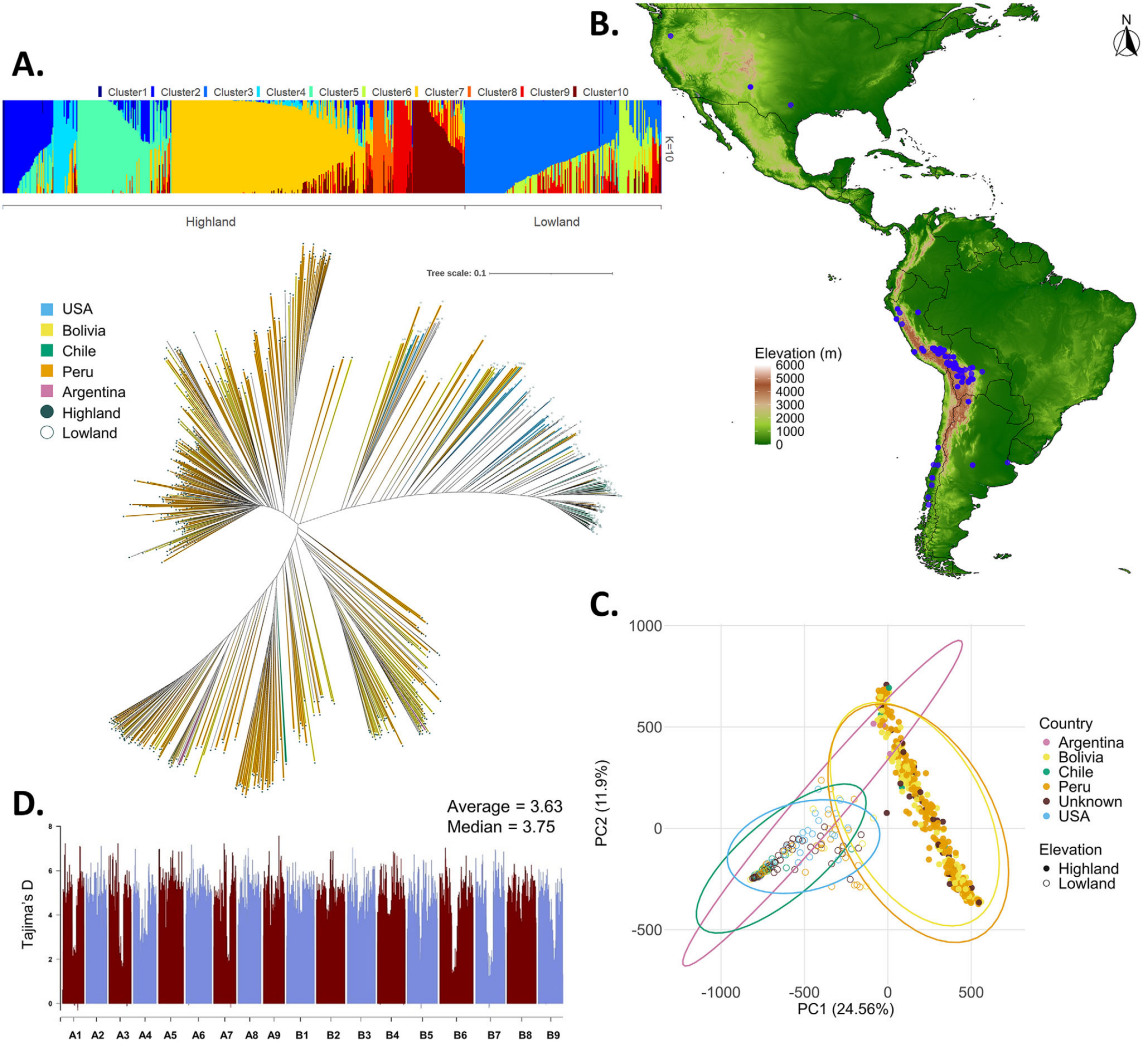
Genomic diversity of 603 *C. quinoa* accessions. (A) Neighbor‐joining tree of 603 accessions with inferred population structure (*K* = 10, corresponding to the model complexity that maximized marginal likelihood) using 1.45 m single‐nucleotide polymorphisms (SNPs), grouped by geographic origin. (B) Geographic distribution of quinoa accessions across the Americas, with collection sites (blue dots) shown in relation to altitude. Map data: Natural Earth (public domain); geocoding: OpenStreetMap contributors; elevation: SRTM DEM. (C) Principal component analysis (PCA) of 603 accessions using 1.45 m SNPs, color‐coded by geographic origin and shaped by elevation, illustrating genetic stratification largely along an altitudinal gradient. PCA plot displays the first two principal components with percentage variance explained, each point represents one accession. (D) Genome‐wide Tajima's D values calculated in 100 kb sliding windows, showing predominantly positive values indicative of high genetic diversity and balancing selection within the population.

Population structure analysis fastStructure showed that *K* = 1 was sufficient to explain the major structure present across the quinoa diversity panel, indicating limited broad‐scale differentiation among accessions. However, the model complexity that maximized marginal likelihood was *K* = 10, supporting the presence of finer‐scale genetic subdivision within the panel rather than deeply divergent lineages. Geographic assignment revealed that clusters 2, 4, 5, 7, 8, 10 were enriched for highland accessions, whereas clusters 3 and 6 largely lowland (Figure 1A and Figure S2), suggesting an association between elevation and population differentiation. Consistent with this pattern, clusters 4, 5, 7, and 10 were mainly composed of Bolivian and Peruvian accessions, whereas cluster 6 was largely Chilean (Figure S1B). Cluster 5 was predominantly associated with bitter quinoa accessions, while cluster 7 was enriched for sweet types. By contrast, saponin content did not show a clear correspondence with population structure.

Genomic principal component analysis (PCA) explained 24.6% of the total genetic variance, primarily reflecting geographic origin: Chilean and U.S. accessions formed a distinct cluster from Bolivian and Peruvian accessions [15], a pattern closely correlated with the collection site in highland or lowland regions (Figure 1B,C). This suggests altitude as a major driver of population differentiation. Within‐group variance accounted for 11.9% of the total genetic variation, a pattern that was recapitulated using a representative subset of accessions for later GWAS analysis (Figure S1C). To assess potential heterogeneity, Tajima's D was examined across chromosomes and genomic windows revealing broadly consistent positive values (mean = 3.63, median = 3.75; Figure 1D and Table S1), indicating an excess of intermediate‐frequency alleles across the genome. While such patterns may be consistent with balancing selection or historical population admixture, they may also reflect underlying population structure or demographic history in the panel. Data completeness was high, with 532 accessions retaining >90% of SNP calls (Figure S1D), and inbreeding coefficients exceeded 0.72 in 464 accessions (Figure S1D and Table S2), confirming extensive homozygosity and validating the suitability of this panel for high‐resolution genome‐wide association studies.

### 2.2 Multi‐tissue Metabolic Diversity in the Quinoa GWAS Panel

We profiled specialized metabolites and lipids in seeds of 581 *C. quinoa* accessions, and specialized metabolites in roots and leaves of a subset of 166 accessions using LC–MS. In total, we detected 4688 polar secondary metabolic features and 4949 apolar lipid features in seeds, 1203 secondary metabolic features in roots, and 608 in leaves (Figure 2A). PCA revealed clear tissue‐specific metabolic variation, with seeds explaining 13.1% and 6.9% of variance along PC1 and PC2, respectively, leaves 18.4% and 10.6%, and roots 17.1% and 10.8%. Importantly, secondary metabolite variation in seeds mirrored genomic structure, with highland accessions separating from lowland accessions (Figures 1C and 2A). This trend was observed in the polar but not the apolar seed fraction (Figure S3).

**FIGURE 2:**
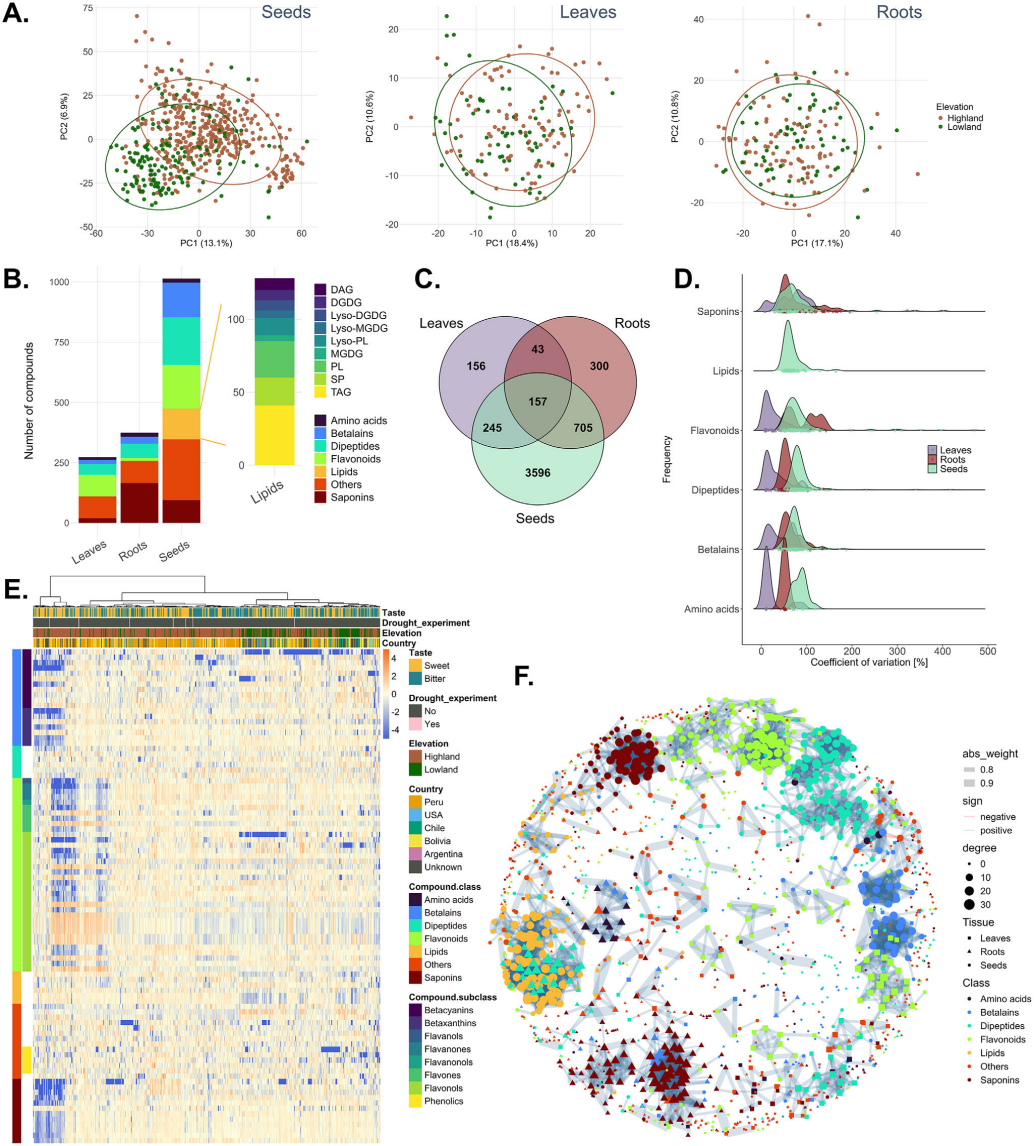
Metabolic diversity of the *C. quinoa* diversity panel across seeds, leaves, and roots. (A) Principal component analysis of 4706 seed, 609 leaf, and 1210 root metabolic features from 581, 166, and 167 accessions, respectively. Accessions are color‐ or shape‐coded by geographic origin or elevation. PCA plot displays the first two principal components with percentage variance explained, each point represents one accession. (B) Distribution of compound classes per tissue (excluding unknowns): 275 in leaves, 375 in roots, and 1014 in seeds, including 128 lipids. Data are shown as counts. (C) Venn diagram of shared and tissue‐specific metabolites across seeds, leaves, and roots. (D) Ridgeline plot of the coefficient of variation [%] for leaf (purple, *n*_amino acids_ = 13, *n*_betalains_ = 17, *n*_dipeptides_ = 45, *n*_flavonoids_ = 90, *n*_saponins_ = 20), root (red, *n*_amino acids_ = 18, *n*_betalains_ = 28, *n*_dipeptides_ = 59, *n*_flavonoids_ = 12, *n*_saponins_ = 166), and seed (green, *n*_amino acids_ = 17, *n*_betalains_ = 143, *n*_dipeptides_ = 198, *n*_flavonoids_ = 181, *n*_lipids_ = 128, *n*_saponins_ = 95) secondary metabolites. (E) Heatmap of 96 annotated seed metabolites with significant genomic associations, including 18 betalains, 7 dipeptides, 38 flavonoids, 6 lipids, 12 saponins, and 15 other metabolites like sugars or organic acids. Accessions are annotated by taste type (sweet/bitter), origin, and elevation; those used in the drought experiment are highlighted in pink (Ames‐13760, LM2, CHEN‐199, D‐12393, CHEN‐384, D‐12165, PI‐665276). Heatmap represents normalized metabolite abundances. (F) Network analysis of annotated seed, leaf, and root secondary metabolites and lipids (for *n* see (D), *n*_others‐seed_ = 252, *n*_others‐leaf_ = 90, *n*_others‐seeds_ = 92) based on Pearson correlation (|*r*| > 0.7, *p* < 0.05), visualized using the Kamada–Kawai layout.

We annotated 1014 metabolites in seeds, 375 in roots, and 275 in leaves (Figure 2B and Tables S3 and S4). As expected from tissue specialization, seeds were enriched in storage‐related lipids (12.6%, mainly triacylglycerols), betalains (14.1%, primarily betacyanins), flavonoids (17.7%, mostly flavonols), and dipeptides (19.5%). Roots were strongly enriched in saponins (44.2%), while leaves were dominated by flavonoids (32.7%, largely flavonols) reflecting tissue‐specific allocation of defense‐ and photoprotection‐related metabolites. Beyond these expected tissue‐specific signatures, a key insight from the multi‐tissue comparison was the identification of a shared metabolic core: 157 metabolites were detected across all tissues, spanning multiple chemical classes including dipeptides (15.9%), saponins (9.5%), betalains (8.9%), and flavonoids (5.7%) (Figure 2C and Table S5). This indicates that, in addition to strong tissue specialization, a subset of metabolites is broadly distributed across tissues, potentially reflecting conserved metabolic functions, coordinated regulation, or long‐distance metabolite transport.

Pairwise comparisons further revealed asymmetric metabolite sharing between tissues, with 43 metabolites shared between leaves and roots, 245 between leaves and seeds (notably flavonoids), and 705 between roots and seeds (enriched for saponins), indicating differential degrees of metabolic overlap. Notably, the strong overlap between roots and seeds was enriched for saponins, suggesting possible long‐distance transport, coordinated biosynthetic regulation of these compounds or shared metabolic storage functions.

Variation within compound classes, quantified by the coefficient of variation (CV), highlighted amino acids, betalains, and dipeptides as highly variable (mean CV: 85.1%, 75.3%, and 86.2%, respectively). Flavonoids showed greatest variability in roots (90.9%) and seeds (85.9%), while saponins were highly variable in both roots (78.7%) and seeds (78.3%) (Figure 2D). Heatmap analyses underscored extensive diversity across accessions, particularly in dipeptides, flavonoids, and saponins (Figure 2E and Figure S4). In seeds, a distinct subgroup of accessions diverged strongly from the main population, driving separation along PC1 (Figure 2A,E).

Network analysis resolved 19 major metabolic clusters (≥10 compounds) using Louvain community detection (Figure 2F and Table S6). These included three saponin clusters (two root‐enriched, one seed‐associated), four dipeptide clusters (two seed, one root, one leaf), three lipid clusters (seed), and four flavonoid clusters (two seed, two leaf). Additional clusters comprised betalains (two), amino acids (one), and mixed compound classes (two). For example, ferulate strongly correlated with paselate methyl ester (*r* = 0.78), while together with ferulate isorhamnetin‐3,7‐*O*‐glucoside correlated with *p*‐coumaroylmalate (*r* = 0.80, 0.71). The other mixed leaf cluster highlighted strong correlations among arginine, glutamyl‐arginine, histidine, pyroglutamate, and proline‐betaine (*r* ≥ 0.71).

We further identified a secondary metabolite (m/z 505.227, retention time 4.68 min) that showed strong association with the sweet versus bitter classification of quinoa accessions, consistent with previously reported taste‐associated chemotypes [15, 53]. Rather than representing a confirmed causal determinant of taste, this feature should be interpredeted as a putative marker correlated with the underlying metabolite composition associated with bitterness, including known saponin variation. Using this feature, accessions were stratified into sweet and bitter groups, enabling comparative analysis of metabolite profiles across tissues (Figure S5 and Table S7).

Rather than representing a single dominant marker of taste, differential abundance analysis revealed coordinated metabolic shifts between sweet and bitter accessions across tissues. In seeds, 58 betalains, 74 dipeptides, 114 flavonoids, 47 saponins, 48 lipids, six amino acids, and 90 other metabolites (including sugars, organic acids, and amino acid derivatives) differed significantly between groups. In roots, differences were primarily driven by saponins (53 compounds), along with smaller contributions from dipeptides, betalains, and other metabolites. In leaves, differences were associated with flavonoids (13 compounds), saponins, and dipeptides, indicating that taste‐associated metabolic variation is not restricted to seeds but is reflected across multiple tissues.

Within these tissue‐specific patterns, several individual metabolites showed particularly strong group‐specific differences. Notably, the saponin phytolaccoside E was more abundant in leaves of bitter accessions (*FC* = 0.47), while quercetin‐dihexoside showed higher abundance in seeds (*FC* = 2). In addition, multiple flavonoids, including quercetin‐3‐*O*‐2‐*O*‐galloyl‐arabinoside, kaempferol‐hexoside, and luteolin, as well as soyasaponin‐II, were enriched in leaves (*FC* > 2). Roots showed strong enrichment of saponins, including hederagenin‐pen‐3x hex, goyasaponin‐III, licoricesaponin A3, cloversaponin‐I, licoricesaponin E2, and betalains gomphrenin‐I, and isogomphrenin‐I (*FC* > 2). Together, these metabolites reflect coordinated shifts in flavonoid‐ and saponin‐associated pathways across tissues rather than isolated compound effects, suggesting that the observed taste‐associated marker likely captures broader metabolic network variation.

To explore metabolite divergence between domesticated quinoa and its wild relative, we performed an exploratory untargeted metabolite comparison of *Chenopodium suecicum*. Among 828 shared polar metabolic features, 208 compounds were structurally annotated (Figure S6), of which 88 exhibited significant differences in abundance between *C. suecicum* and the *C. quinoa* diversity panel. PCA positioned *C. suecicum* within the broader variation of *C. quinoa* accessions, with variation along PC1 (16.1%) largely driven by saponin abundance. This pattern is consistent with the central role of saponins in differentiating wild and domensticated *Chenopodium taxa*. Although the present data do not allow formal inference of evolutionary history, the observed saponin‐associated differentiation is compatible with the possibility that divergence in saponin content emerged after the hybridization events leading to modern quinoa or may reflect contributions from other wild progenitors, such as *Chenopodium pallidicaule*. When compared to a single quinoa accession (CHEN‐543), PCA showed a clearer separation, explaining 89.6% of the variance (Figure S7). We note that CHEN‐543 was included as an available reference genome accession and not selected as a representative of quinoa diversity in this study; therefore, this comparison should be interpreted as illustrative rather than population‐representative. Overall, 474 metabolites differed significantly in abundance between *C. suecicum* and *C. quinoa* (adjusted *p* < 0.05, |log_2_ *FC*| > 1), of which 119 could be assigned to metabolic classes including amino acids, betalains, flavonoids, dipeptides, and saponins.

Together, these results reveal extensive metabolic diversity across tissues in the quinoa diversity panel, highlight tissue‐specific clustering of major metabolite classes, and identify metabolite features associated with sweet and bitter seed chemotypes. Comparative metabolic profiling of *C. suecicum* further revealed pronounced divergence in metabolite composition, particularly in saponin‐associated profiles. Although the present data do not allow formal inference of evolutionary history, these patterns are compatible with the possibility that saponin‐related divergence emerged during the domestication and hybridization processes leading to modern quinoa or may reflect contributions from other wild progenitors.

### 2.3 Genome‐Wide Association Study of the Quinoa Metabolome

We performed GWAS on 4688 polar secondary metabolite features and 4949 lipid‐related apolar features in seeds, as well as 1203 and 608 polar secondary metabolite features in roots and leaves, respectively. Six complementary models were applied (see materials and methods). Metabolites were first prefiltered using factored spectrally transformed linear mixed model (FaST‐LMM) with genotype binning and Haseman–Elston (HE) as the variance component, and only associations surpassing a Bonferroni threshold were carried forward. Subsequent analyses employed mixed linear model (MLM), CMLM, MLMM, FarmCPU, and BLINK (Figure S8). To ensure robustness, only SNPs identified by at least four methods were retained (Tables S8 and S9). Using this conservative framework, we identified 615 high‐confidence marker‐trait associations (MTAs). These comprised 82 MTAs for 119 seed apolar metabolites, 365 MTAs for 652 seed polar metabolites, 137 MTAs for 209 root metabolites, and 46 MTAs for 44 leaf metabolites (Tables S9–S11). As individual metabolites may map to multiple loci, while individual loci can simultaneously affect multiple metabolites, the number of MTAs is not expected to scale directly with the number of significantly associated metabolites. In total, 295 MTAs corresponded to annotated compounds (26 leaf, 66 root, 205 seed MTAs; Tables S9 and S10). Across tissues, the strongest enrichment of associations was observed for saponins, which accounted for 163 seed MTAs and 53 root MTAs, indicating that saponin metabolism represents a major genetically structured component of the quinoa metabolome. In contrast, flavonoids contributed substantial numbers of associations across all tissues, particularly in seeds (39 MTAs) and leaves (9 MTAs), suggesting both shared and tissue‐specific genetic control of specialized metabolite accumulation. Overall, the distribution of associations across many metabolites and chemical classes indicates that the genetic architecture of the quinoa metabolome is predominantly polygenic. However, the repeated detection of multiple associations within particular metabolite classes, especially saponins, also points to loci with comparatively stronger and more coordinated effects on specific biosynthetic pathways.

To establish a framework for candidate gene prioritization of metabolic traits, we integrated GWAS results with complementary layers of genomic and functional evidence, including comparative genomics (OrthoFinder, Figure S9), published transcriptomic resources, and newly generated transcriptomic and proteomic datasets from a drought experiment involving seven genetically diverse accessions selected based on seed metabolic profiles (Figure 6). These datasets were used to provide independent functional context for GWAS‐identified loci rather than to rely on positional association signals. As part of this framework, we applied weighted gene co‐expression network analysis (WGCNA), which identified 46 distinct co‐expression modules that organized into four major clusters. WGCNA was included at this stage because module membership information was subsequently used as an additional criterion for candidate gene prioritization in the downstream locus‐specific analyses.

Together, the multi‐omic datasets generated here establish a framework that integrates genetic associations with transcriptomic, proteomic, and co‐expression evidence, enabling more informed prioritization of candidate genes underlying metabolic diversity in quinoa.

### 2.4 Genomic Hotspots and Candidate Genes Underlying Metabolic Diversity

To validate the robustness of our mapping, we recovered TSARL1 and TSARL2 as candidate genes in a major hotspot QTL on chromosome B5 (Figure 3A, Figure S10A; Figure S11B,C; Tables S8–S11). This is consistent with previous reports identifying this locus as a key QTL for saponin abundance [1, 15]. Interestingly, adjacent to these genes, we additionally detected six UDP‐glycosyltransferases (UGTs; *CQ004358*, *CQ004359*, *CQ004360*, *CQ004361*, *CQ004362*, *CQ004365*), three of which were differentially expressed under drought conditions and belong to the black (*CQ004358*), tan (*CQ004359*), and blue module (*CQ004361*). In seeds, major hotspot regions were detected (Figure 3A and Tables S9 and S12) on chromosome A4, harboring two NAC domain‐containing proteins (CQ036847, CQ036858) and an arabinosyltransferase (CQ036871); chromosome A5 containing a flavonoid 3′‐monooxygenase (CQ031304); chromosome B2 with an ABC transporter (CQ014552) and a laccase (CQ014575); chromosome B7 containing a MYB transcription factor (CQ007594), cinnamyl alcohol dehydrogenase (CQ007599) and two UDP‐glycosyltransferases (CQ007602, CQ007604); and chromosome B9 harboring a delta(24)‐sterol reductase (CQ034816), four chalcone synthases (CQ034822, CQ034825, CQ034827, CQ034828), two monooxygenases (CQ034830, CQ034832), and a 2‐hydroxyisoflavanone dehydratase (CQ034839). In roots, prominent hotspot regions were observed on chromosome B2 containing an ABC transporter (CQ013414) and zeaxanthin epoxidase (CQ013418), as well as chromosome B3 harboring two terpene synthases (CQ018501, CQ018502) and a sesquiterpene synthase (CQ018505). The concentration of metabolite‐associated loci within these genomic intervals suggests coordinated genetic regulation of specialized metabolic pathways and highlights key regions contributing to metabolic diversification.

**FIGURE 3:**
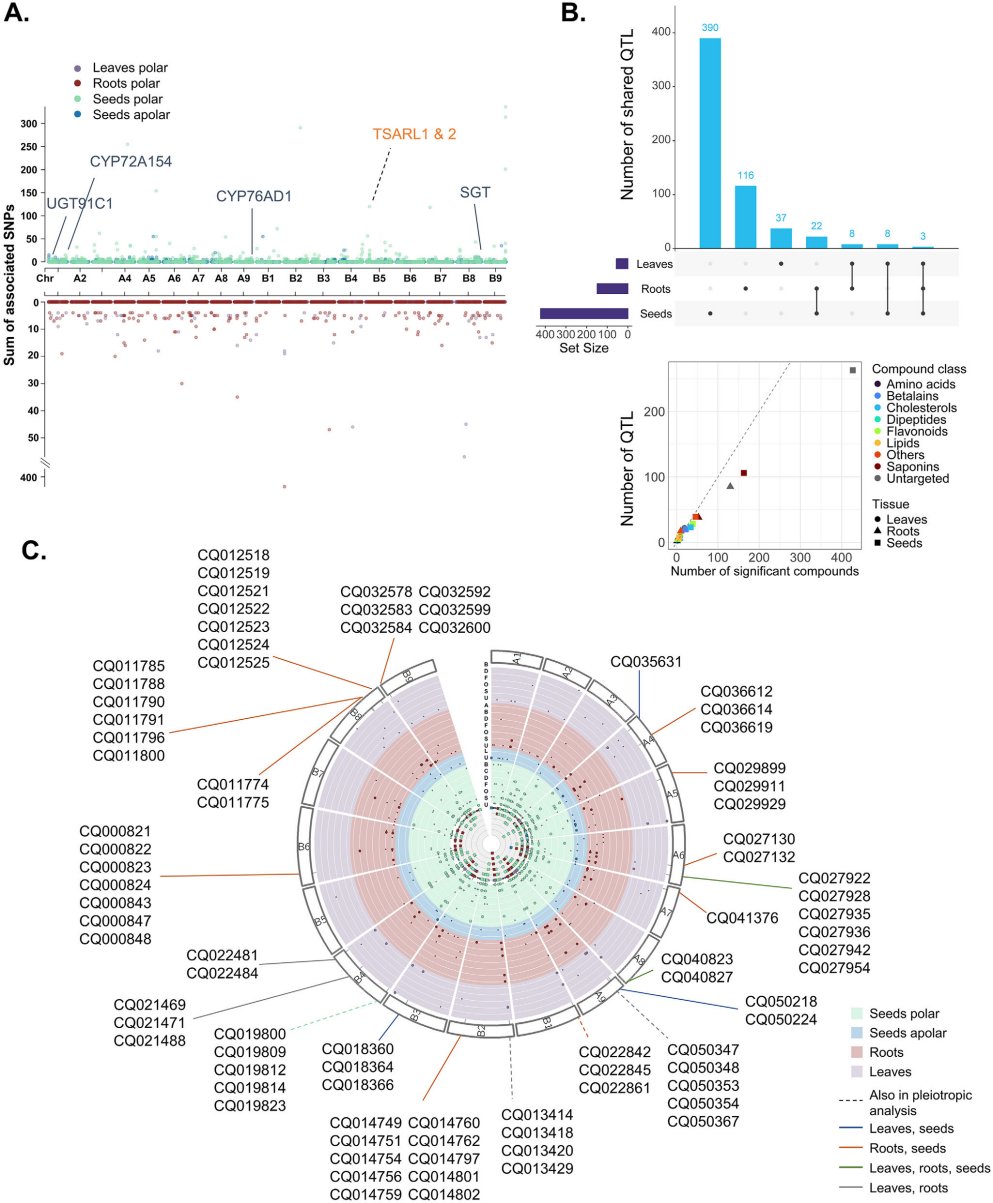
Multi‐tissue QTLome of *C. quinoa* secondary metabolites. (A) Number of metabolic features with significant genomic associations across tissues: 652 seed secondary metabolites, 119 seed lipid features, 44 leaf secondary metabolites, and 209 root secondary metabolites, detected in 573, 166, and 167 quinoa accessions, respectively. In total, 365, 82, 46, and 137 marker‐trait associations (MTAs) were identified for seed secondary metabolites, seed lipids, and leaf and root secondary metabolites, respectively. Each dot represents a significant genomic association. (B) Intersections of QTL in a 50 kb sliding window across tissues. As input the sum of the marker‐trait associations across compound classes was used. In total 584 QTL were detected with 41 overlaps. Number of identified QTL in a 50 kb sliding window versus number of compounds with a GWAS association grouped by compound class and shaped by tissue. Data are shown as counts. Each point represents one compound class. (C) Pleiotropic analysis in a 50 kb window size of seed polar and apolar metabolites, root metabolites, and leaf metabolites with their candidate genes. The outer ring represents the chromosomes; the inner rings show compound classes per tissue; and the dots indicate SNPs associated with genomic regions. The innermost circle summarizes the total number of associations per QTL. Compound class abbreviations: A = amino acids, B = betalains, D = dipeptides, F = flavonoids, L = lipids, O = other compounds, S = saponins, U = unknown.

In addition, GWAS for elevation revealed three QTL overlapping with the genomic PC1 reported by Patiranage et al. [15], largely reflecting adaptation to altitude (Figure S9B). Given the role of oxygen limitation at high elevations [54, 55], we further investigated hypoxia‐related pathways and identified 35 candidate genes involved in hormone signaling, development, and transcriptional regulation (Table S12).

In total, we identified 584 QTL associated with secondary metabolites across seed, root, and leaf tissues (Figure 3B). Among these, 41 QTL were shared between tissues, while 390, 116, and 37 QTL were uniquely detected in seeds, roots, and leaves, respectively. Notably, for seed and root saponins as well as untargeted compounds, the number of detected QTL was lower than the number of significant metabolites, suggesting that multiple compounds may co‐localize to shared genomic regions. This pattern is consistent with the hypothesis that some of these loci may represent regulatory QTL with pleiotropic effects influencing coordinated metabolic pathways. Most QTL, SNPs, and significantly associated compounds were linked to saponins in roots and seeds, and to flavonoids in leaves (Figure S11A). Across compound classes, we detected 493, 157, and 49 QTL in seeds, roots, and leaves, respectively, of which 148, 46, and 6 QTL were shared among multiple compound classes (Figure S12A). The genomic distribution of QTL revealed major hotspots on chromosomes A1, A4, A5, and B8 for seed metabolites; A4, A6, A7, B3, B6, and B7 for root metabolites; and A1, A4, A9, B8, and B9 for leaf metabolites (Figure S12B).

Genome‐wide hotspot mapping, based on the density of significant associations and pleiotropic signals across tissues, revealed 58 novel QTL comprising 219 candidate genes (Figure 3C; Figure S13; Table S12). These included, among other gene families, 26 UGTs, 20 transporters, 34 cytochrome P450 enzymes, 34 transcription factors (including nine basic helix–loop–helix [bHLH]), and 23 sugar‐, methyl‐, or acetyltransferases. Tissue‐specific candidates comprised 10 candidate genes in leaves, 11 in roots, and 112 in seeds, while pleiotropic analyses identified 14 candidates shared between leaves and roots, 50 between roots and seeds, six between seeds and leaves, and eight common to all three tissues. Two of which, an arginase (*CQ047623*), and fructose‐bisphosphate aldolase (*CQ007727*) were differentially regulated under drought belonging to the blue module of the drought experiment.

Examples of key loci include: (i) a leaf QTL on chromosome 5A at 61.7 Mb associated with ferulate and decarboxybetanin, harboring two CYPs (*CQ032038*, *CQ032039*), two bHLH transcription factors (*CQ032045*, *CQ032046*), and three amino acid transporters (*CQ032055*, *CQ032056*, *CQ032058*) (Figure S13A); (ii) a multi‐tissue QTL on chromosome A9 at 9.2 Mb containing three ABC transporters (*CQ050347*, *CQ050348* [grey module], *CQ050353*), a 4‐hydroxy‐3‐methylbut‐2‐en‐1‐yl diphosphate synthase (*CQ050354*, [blue module]) involved in isoprenoid biosynthesis, and CYP81D1 (*CQ050367*), associated with betalains and saponins in leaves and roots (Figure 4 and Figure S13B); and (iii) two major seed QTL: one on chromosome 4 at 43.2 Mb harboring seven CYPs, including 11‐oxo‐β‐amyrin 30‐oxidase (*CQ036986*), three CYP72A219 (*CQ036984*, *CQ036987*, *CQ036990*), and three CYP72A15 (*CQ036985*, *CQ036996*, *CQ037000*) (Figure S13C); and another on chromosome B8 at 9.2 Mb comprising 12 CYPs (*CQ010896* – *CQ010921* [*CQ010911* pink module; *CQ010912* brown module; *CQ010921* turquoise module]) and two bHLH transcription factors (*CQ010905*, *CQ010906*), associated with sterol‐ and saponin‐derived metabolites.

**FIGURE 4:**
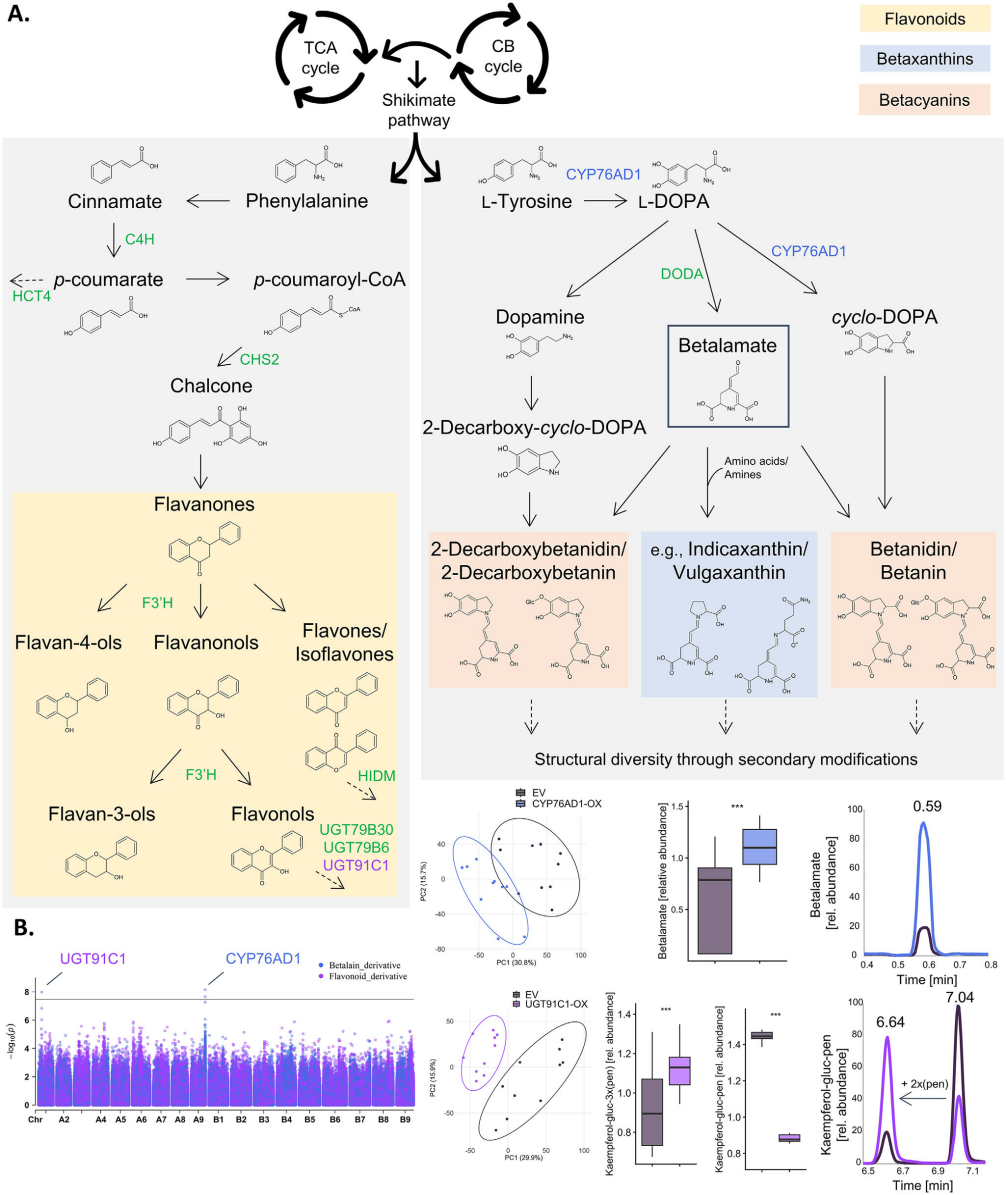
Refinement of the flavonoid and betalain pathways in *C. quinoa*. (A) The flavonoid pathway originates from phenylalanine and proceeds through phenylpropanoid intermediates such as cinnamate, *p*‐coumarate, *p*‐coumaroyl‐CoA, and chalcone. From these precursors, diverse flavonoid structures are synthesized, including flavanones, flavan‐4‐ols, flavanonols, flavones and isoflavones, flavan‐3‐ols, and flavonols. The betalain pathway, in contrast, derives from L‐tyrosine via L‐DOPA, *cyclo*‐DOPA, betalamate, and dopamine, leading to the formation of betacyanins (e.g., betanidin, betanin) and betaxanthins (e.g., indicaxanthin, vulgaxanthin). These core molecules serve as substrates for secondary modifications such as glycosylation and oxidation. Candidate genes are marked in green, validated genes are colored in purple and blue. (B) Overlay Manhattan plots for a representative betalain derivative and a flavonoid derivative highlight the QTL associated with CYP76AD1 and UGT91C1, respectively. Each dot represents a single‐nucleotide polymorphism with −log10(*p*) from the genome‐wide association study. Principal component analysis (*n*_samples_ = 10, *n*_metabolites‐CYP76AD1_ = 6627, *n*_metabolites‐UGT91C1_ = 6040) of transiently overexpressed (OX) lines compared to empty vector (EV) controls in *C. quinoa* demonstrates clear metabolic differentiation. Both genes were functionally validated, as shown by the boxplots (*n* = 10) and chromatograms of betalamate and the addition of two pentosides (pen) to kaempferol‐glucuronide‐pentoside (kaempferol‐gluc‐pen). PCA plots display the first two principal components with percentage variance explained, each point represents a sample. Boxplots show median, interquartile range (IQR), and 1.5× IQR whiskers. Asterisks indicate significance ***p < 0.001, Wilcoxon test. Abbreviations: TCA, tricarboxylic acid cycle; CB, Calvin–Benson cycle; C4H, cinnamate 4‐hydroxylase; HCT4, hydroxycinnamoyltransferase 4; CHS, chalcone synthase; F3′H, flavonoid 3′‐hydroxylase; HIDM, 2‐hydroxyisoflavanone dehydratase; UGT, UDP‐glycosyltransferase; L‐DOPA, L‐3,4‐dihydroxyphenylalanine; DODA, L‐DOPA 4,5‐dioxygenases; CYP, cytochrome P450.

### 2.5 Candidate Genes Underlying Flavonoid, Betalain, and Saponin Biosynthesis

To investigate the genetic architecture underlying metabolic biosynthetic pathways in quinoa, next, we focused on three key classes of specialized metabolites – flavonoids, betalains, and saponins (Figures 4 and 5). For flavonoids, we identified seven candidate genes involved in flavonoid and phenylpropanoid biosynthesis. On chromosome B2, a QTL shared between roots and seeds encompassed cinnamate 4‐hydroxylase (C4H; *CQ014797*), which catalyzes the conversion of *trans*‐cinnamate to *p*‐coumarate, and hydroxycinnamoyltransferase 4 (HCT4; *CQ014802*), a key enzyme in lignin precursor formation. Additional QTL on chromosomes B8 and B9 contained one and four chalcone synthase (CHS) genes (*CQ011785*, *CQ034822*, *CQ034825*, *CQ034827*, *CQ034828*) associated with flavonoid accumulation in seeds and roots. The same region on B9 also included 2‐hydroxyisoflavanone dehydratase (HIDM; *CQ034839*), which catalyzes the dehydration of isoflavones. In seeds, a major QTL on A5 harbored flavonoid 3′‐monooxygenase (F3′H; *CQ031304*), while another on B5 (adjacent to TSARL1 and 2) comprised five UGT79B30 glycosyltransferases (*CQ004358*, *CQ004359*, *CQ004360*, *CQ004361*, *CQ004365*) previously implicated in flavonol glycosylation in *Glycine max* [56]. Similarly, a QTL on B6 contained three UGT79B6 genes (*CQ000822*, *CQ000823*, *CQ000824*), known for flavonol glycosylation in *Arabidopsis thaliana* [57], co‐localizing with one bHLH (*CQ000843*), and two ICE transcription factors (*CQ000847*, *CQ000848*), suggesting coordinated transcriptional control of flavonoid modification and accumulation. We further identified UGT91C1 (*CQ048026*; chromosome A1, 11 Mb), associated with a flavonoid derivative and annotated as a soyasaponin III rhamnosyltransferase orthologue in beetroot (*Beta vulgaris*). To provide more insight and functionally validate their roles in the biosynthetic pathways, transient overexpression in both *Nicotiana benthamiana* and *C. quinoa* resulted in a metabolic alteration, which was more pronounced in *C. quinoa* (Figure 4B and Figure S14). While no significant changes were observed in saponin levels (Figure S15), differential effects were evident among flavonoids: kaempferol‐glucuronide‐pentoside accumulated more in the empty vector control, whereas kaempferol‐glucuronide‐3x(pentoside) was enriched in UGT91C1‐overexpressed (OX) lines, supporting a role for UGT91C1 in flavonoid glycosylation and decoration.

**FIGURE 5:**
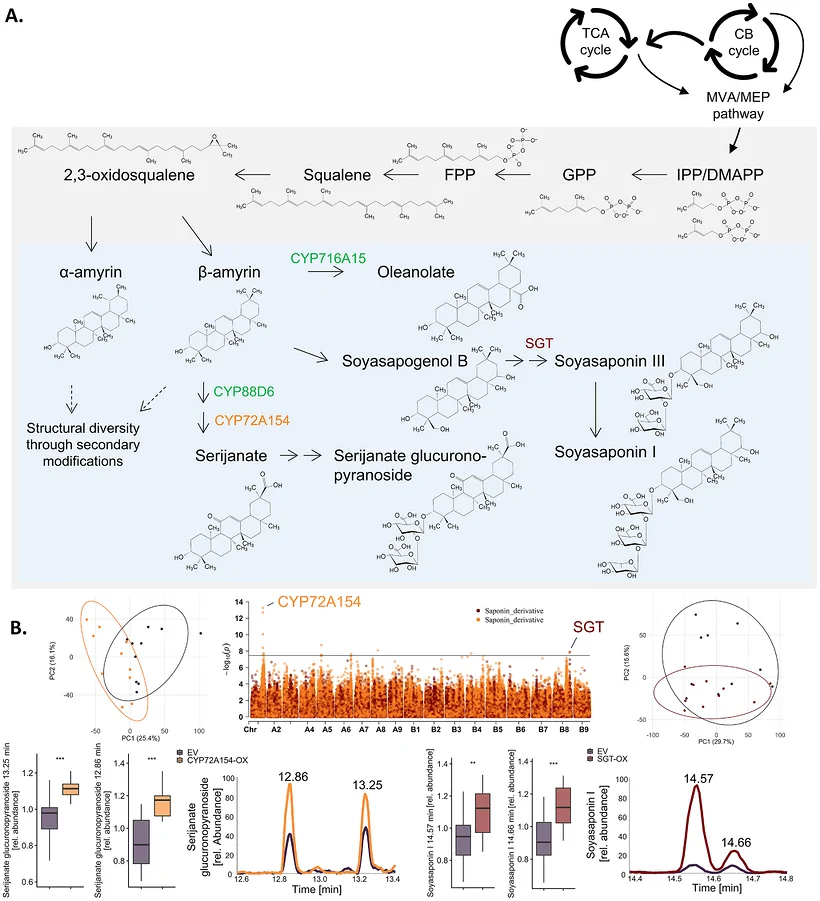
Refinement of the saponin pathway in *C. quinoa*. (A) The saponin pathway originates from the mevalonate (MVA) and methylerythritol phosphate (MEP) cytosolic and plastidic pathways, respectively, and proceeds through multiple condensation reactions of isopentenyl pyrophosphate (IPP)/ dimethylallyl pyrophosphate (DMAPP) forming the saponin precursor molecules α‐ and β‐amyrin. Example of structural diversity through secondary modification are oleanolate which is synthesized through C‐28 hydroxylation by CYP716A15, and serijanate glucuronopyranoside and soyasaponin by hydroxylation and glycosylation by soyasapogenol B glucuronide galactosyltransferase (SGT), CYP88D6, and CYP72A154. Candidate genes are marked in green, validated genes are colored in red and orange. (B) Overlay Manhattan plots for a representative saponin derivatives highlight the QTL associated with CYP72A154 and SGT. Each point represents a single‐nucleotide polymorphism with −log10(*p*) from the genome‐wide association study. Principal component analysis (*n* = 10) of transiently OX lines compared to EV controls in *C. quinoa* demonstrates metabolic differentiation. Both genes were functionally validated, as shown by the boxplots (*n*_samples_ = 10, *n*_metabolites‐CYP72A154_ = 5100, *n*_metabolites‐SGT_ = 7074) and chromatograms of serijanate glucuronopyranoside and soyasaponin I. PCA plots display the first two principal components with percentage variance explained, each point represents a sample. Boxplots show median, IQR, and 1.5× IQR whiskers. Asterisks indicate significance **p < 0.01, ***p < 0.001, Wilcoxon test. Abbreviations: GPP, geranyl pyrophosphate; FPP, farnesyl pyrophosphate.

For betalains, we identified a QTL on B4 contained five L‐DOPA 4,5‐dioxygenases (DODA; *CQ020378*, *CQ020379*, *CQ020383*, *CQ020384*, *CQ020385*), which convert L‐DOPA to betalamate, co‐localizing with an extradiol ring‐cleavage dioxygenase (*CQ020383*) and four UGTs (*CQ020393*, *CQ020394*, *CQ020395*, *CQ020397*), forming a tightly co‐regulated cluster likely responsible for betalain biosynthesis and modification. Additionally, two major seed QTL on chromosomes A8 and A9, each containing CYP76AD1 (*CQ039659*, *CQ052677*, *CQ052678*, *CQ052696*, *CQ052698*, *CQ052697*), which catalyzes the sequential oxidation of l‐tyrosine to l‐DOPA and *cyclo*‐DOPA. The A9 QTL, harboring the functionally cloned CYP76AD1 (*CQ052697*), also included two bHLH transcription factors (*CQ052675*, *CQ052676*) at 53.7 Mb. This locus was associated with 2‐decarboxybetanin and several unknown polar metabolites. By transient overexpression in *C. quinoa*, we were able to functionally validate its role in betalain biosynthesis (Figure 4B). PCA revealed that PC1 accounted for 30.8% of the variation, clearly separating the overexpression line from the control, whereas in *N. benthamiana* no distinct separation was observed, even when co‐infiltrated with DODA (Figure S14). In total, 56 metabolites – including betalains, flavonoids, and saponins – were differentially abundant upon overexpression, of which 14 were betalains (Figure S15). Notably, betalamate levels were significantly higher in the overexpression line compared to the empty vector control (Figure 4B), confirming the gene's role in betalain biosynthesis.

For the triterpenoid saponin biosynthetic pathway, we identified four genes directly involved in saponin formation (Figure 5). A prominent QTL shared between roots and seeds on chromosome B2 harbored CYP716A15 (*CQ014759*), a β‐amyrin 28‐monooxygenase implicated in oleanolate synthesis in *Vitis vinifera* [58]. Another QTL on chromosome A8 (33.4 Mb) contained CYP88D6 (*CQ039380*), encoding a β‐amyrin 11‐oxidase that introduces a hydroxyl group at the C‐11 position of the triterpene scaffold. Here, two additional candidate genes were cloned and functionally validated through transient overexpression in *C. quinoa* (Figure 5B). These included: (i) CYP72A154 (*CQ049330*; chromosome A1, 47 Mb), which catalyzes the subsequent oxidation at C‐30 to produce serijanate, and (ii) a soyasapogenol B glucuronide galactosyltransferase (SGT; *CQ012413*; chromosome B8, 67.8 Mb), responsible for the galactosylation of soyasapogenol B. Both genes conferred discrete metabolic signatures upon overexpression, as evidenced by PCA in *N. benthamiana* (Figure S14) and an even more pronounced effect in *C. quinoa* (Figure 5B). Their overexpression significantly altered the abundance of 76 and 45 metabolites, respectively, including betalains, flavonoids, and saponins (Figure S16), with serijanate and soyasaponin I being significantly increased in the CYP72A154‐OX and SGT‐OX lines relative to empty vector controls (Figure 5B).

Together, these functionally supported loci provide an entry point for dissecting saponin biosynthesis in quinoa. However, additional genes among the 219 candidates identified across 58 QTL are likely to contribute to the biosynthesis, transport, or modification of flavonoids, betalains, and saponins. These findings underscore both the complexity of specialized metabolism in quinoa and the current limitations of genome annotation for this species.

### 2.6 Multi‐omic Validation of Drought‐Responsive Candidate Genes

To provide functional context for candidate genes prioritized through GWAS, we conducted a drought experiment using seven quinoa accessions selected to capture broad seed metabolic diversity (Figure 2E and Figure S15). This experiment was not performed to provide a separate analysis of drought adaptation, but to assess whether GWAS‐prioritized candidate genes also show transcriptional or proteomic responsiveness under an environmentally relevant perturbation. For that, plants were grown under both greenhouse and polytunnel conditions, experiencing temperatures from 6.7 to 44.9°C and a maximum photosynthetically active radiation (PAR) of 1717 µmol m^−2^ s^−1^ (Figure S16). Drought stress caused significant reductions in growth, fresh and dry biomass, and altered photosynthetic activity in some lines (Figures S17–S21).

Integration of multi‐omics datasets revealed tissue‐ and environment‐specific drought signatures (Table S13). Metabolomics (LC–MS polar/apolar and GC–MS primary metabolites) showed strong tissue‐ and environment‐dependent structuring. PC1 (36.5%) separated polytunnel‐grown leaves from greenhouse leaves, roots, and seeds, indicating a major effect of growth conditions. PC2 (24%) distinguished greenhouse leaves from other tissues, reflecting tissue‐specific metabolic profiles, while PC3 (11.7%) further separated seeds from roots (Figure 6A). These separations were associated with coordinated metabolite shifts, including increased sugars and organic acids in leaves and roots, as well as tissue‐specific enrichment of secondary metabolites classes such as flavonoids, dipeptides, TAGs in leaves, saponins and phospholipids in roots, and betalains and saponins in seeds (Figures S22–S24). Consistently, secondary metabolites contributed most to variance across tissues and conditions compared to primary metabolites and lipids (Figure S25A). Proteomics of leaves and roots explained 59.8% of variance along PC1, reflecting strong tissue separation, while transcriptomics separated seeds from vegetative tissues along PC1 (31.9%) and further resolved tissues along PC2 (23.4%; Figure 6B,C and Figure S25B,C), indicating consistent hierarchical structuring across omics layers.

**FIGURE 6:**
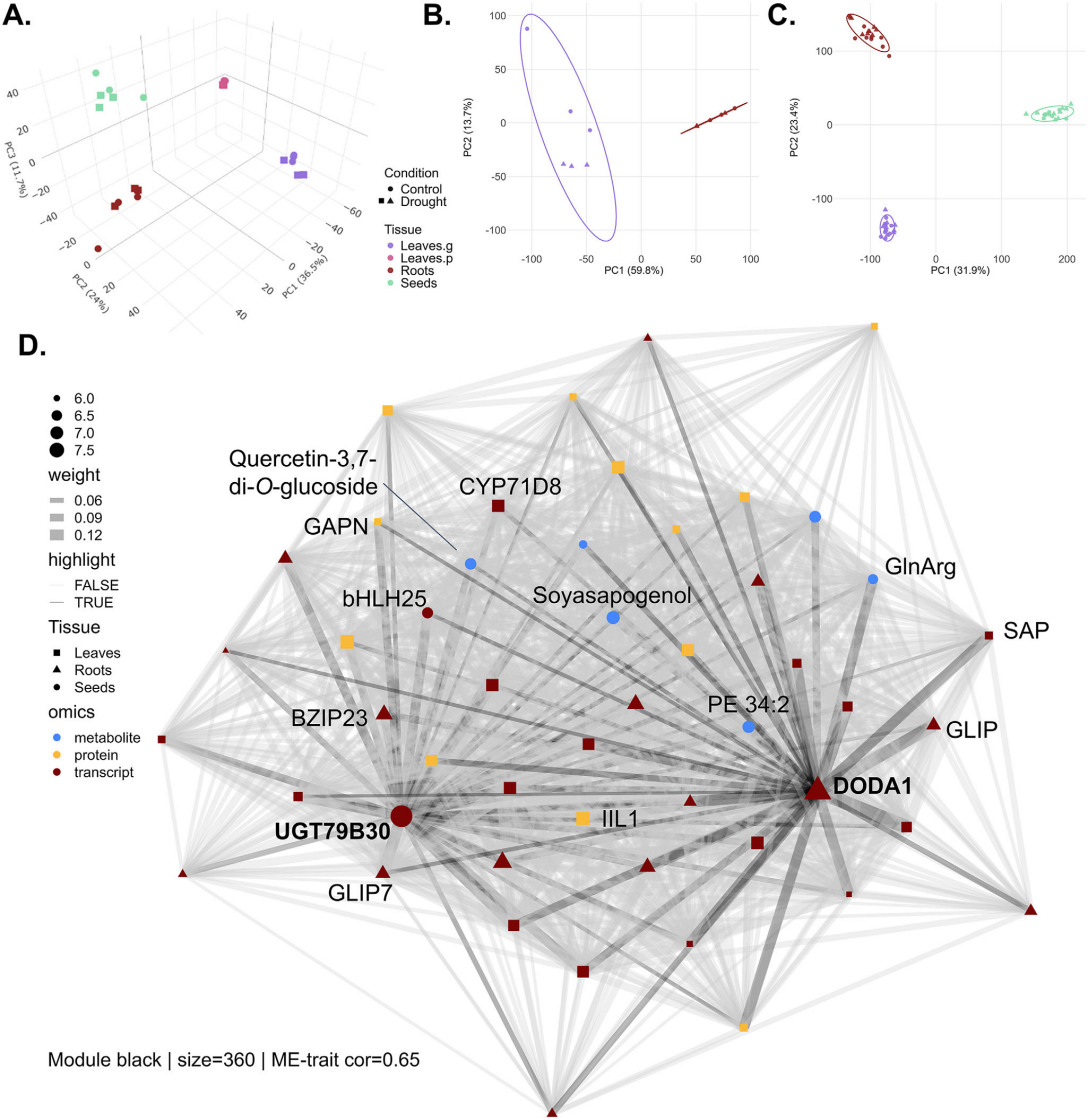
Multi‐omic diversity of three quinoa accessions across tissues. (A) 3D PCA of primary metabolites, secondary metabolites, and lipids (*n* = 5360 metabolites) in leaves (p = polytunnel, g = greenhouse), roots, and seeds of accessions CHEN‐384, LM2, and PI‐665276 (*n*_drought_ = 6, *n*_control_ = 6). (B) PCA of proteomic profiles (*n* = 8,575 proteins) from greenhouse‐grown leaves and roots of the same accessions (*n*_drought_ = 3, *n*_control_ = 3). (C) PCA of transcriptomes (*n* = 51 527 transcripts) from leaves, roots, and seeds under control and drought conditions (*n*_drought_ = 3, *n*_control_ = 3). PCA plots display the first two principal components with percentage variance explained, each point represents an accession under drought or control. (D) Top 50 features of black module (size = 360, module eigengene (ME)‐trait correlation = 0.65, TRUE = positive correlation, FALSE = negative correlation) of weighted gene co‐expression network analysis (WGCNA) of 15 809 metabolic, transcriptomic, and proteomic features across leaf, root and seed tissues of CHEN‐384, LM2, and PI‐665276 identifying 46 modules of four block‐wise clusters. DODA1 and UGT79B30 were identified as candidate genes in GWAS analysis, meaningful correlated features were highlighted. GAPN, NADP‐dependent glyceraldehyde‐3‐phosphate dehydrogenase; GLIP, GDSL esterase/lipase; BZIP, basic leucine zipper; bHLH, basic helix–loop–helix; SAP, sterile apetala; GlnArg, glutamyarginine; PE, phosphatidylethanol; IIL, 3‐isopropylmalate dehydratase large subunit.

WGCNA identified 46 modules (Figures S26 and S27 and Table S14), six of which showed the strongest associations with drought response: turquoise (1663 traits, *r* = 0.92, |GS| = 0.60), black (360 traits, *r* = 0.65, |GS| = 0.51), tan (183 traits, *r* = 0.69, |GS| = 0.49), purple (238 traits, *r* = 0.65, |GS| = 0.48), skyblue (86 traits, *r* = 0.72, |GS| = 0.46), and greenyellow (184 traits, *r* = 0.61, |GS| = 0.44). Functional enrichment revealed distinct biological signatures: the turquoise module was enriched in transcripts and proteins linked to cellular and metabolic processes, strongly correlated with lipids, dipeptides, and saponins; black and tan modules were dominated by proteins associated with metabolic and oxidoreductase activity and molecular/cellular processes, co‐varying primarily with saponins, lipids, and dipeptides; the purple module combined lipids, saponins, and flavonoids with transferase‐related functions; the skyblue module featured seed metabolites including phenylalanine, quercetin‐3,4‐di‐*O*‐glucoside, and miraxanthin‐I, associated with oxidoreductase activity and energy metabolism; and the greenyellow module comprised only proteomic and transcriptomic features enriched in energy generation and small‐molecule metabolism (Figures S28–S32).

The black module included two candidate genes identified via GWAS: DODA1 (*CQ020379*) in roots and UGT79B30 (*CQ004358*) in seeds (Figure 6D). Both genes were embedded within a co‐expression network showing coordinated variation with seed metabolites including soyasapogenol, glutamyarginine, phosphatidylethanol, and quercetin‐3,7‐di‐*O*‐glucoside, as reflected by topological overlap in the WGCNA network. In addition, several other network members exhibited tissue‐specific co‐expression: leaf proteins NADP‐dependent glyceraldehyde‐3‐phosphate dehydrogenase (*CQ055725*) and 3‐isopropylmalate dehydratase large subunit (*CQ021690*); root transcripts GDSL esterase/lipase (*CQ011072*, *CQ015414*) and basic leucine zipper 23 (*CQ021500*); seed transcript bHLH25 (*CQ015892*); and leaf transcripts CYP71D8 (*CQ012278*) and the transcriptional regulator sterile apetala (*CQ050157*).

This multi‐omic integration links GWAS‐identified loci to coordinated transcript‐protein‐metabolite modules, thereby prioritizing candidate genes that are embedded within biologically coherent networks. This framework highlights both previously identified GWAS candidates and additional genes within shared modules, providing a systems‐level context for future functional studies aimed at dissecting regulatory networks underlying metabolic and stress‐associated traits in quinoa.

## 3 Discussion

### 3.1 The Quinoa Diversity Panel Reveals High Genomic and Metabolic Variation

The resequenced *C. quinoa* diversity panel, comprising 603 accessions, was subjected to integrated genomic and metabolic analyses followed by GWAS. This panel included 121 accessions previously profiled for seed metabolites [48] and 310 accessions earlier characterized by sequencing, population structure, and GWAS‐based QTL mapping [15]. The panel was assembled to capture broad geographic and agroecological diversity, including accessions originating from different elevations and environmental backgrounds (Figure 1), which likely contributed to part of the observed metabolomic diversity. However, because all accessions were cultivated under standardized growth conditions, the detected metabolic variation primarily reflects underlying genetic differences rather than direct environmental effects. Similarly, population structure clusters could be assigned to the altitude of the accessions collection site (Figure 1A and Figures S1 and S2). Genome‐wide positive Tajima's D values were observed which reflect an excess of intermediate‐frequency alleles. While such patterns can be consistent with balancing selection, they may also arise from population structure, admixture, or complex demographic history, all of which are present in this diversity panel (Figure 1 and Figures S1 and S2). High inbreeding coefficients (*F* > 0.5) observed in 92% of accessions (Figure S1D) further suggest a predominantly structured and partially selfing population. Notably, genomic variation clearly separated highland accessions from those originating in lowland (Figure 1C), indicating strong geographic structure. Together, these patterns are consistent with historical divergence between elevation‐associated groups; however, additional demographic and phylogenetic analyses would be required to formally test hypotheses of distinct domestication origins.

This genomic stratification was mirrored in the metabolic landscape, particularly among secondary metabolites, with saponins emerging as the most prominent discriminators, corroborating earlier observations [48]. Tissue‐specific profiling revealed root saponins, leaf flavonoids, and both seed saponins and flavonoids as the most abundant metabolite classes (Figure 2), consistent with their ecological roles in defense and photoprotection [43, 51]. In seeds, TAGs, sphingolipids, and PLs dominated the lipidome, reflecting their conserved functions in energy storage, membrane stability, and desiccation tolerance [59, 60, 61]. The pronounced tissue specificity of the quinoa metabolome reflects organ‐specialized functions, including root defense through saponin accumulation, flavonoid‐mediated photoprotection in leaves, and storage‐ and stress‐associated metabolic processes in seeds. The highest CV values were observed for root and seed flavonoids and saponins, highlighting their dynamic evolutionary turnover under both natural and artificial selection [43, 44, 51]. This variability likely reflects local ecological adaptation as well as human‐driven selection for taste, palatability, and reduced antinutritional content.

Despite the large number of annotated compounds identified in this study, most detected metabolic features remain structurally unresolved, highlighting the substantial extent of “metabolic dark matter” within the quinoa metabolome [62, 63]. This is particularly expected for specialized metabolites in underexplored crops, where available spectral databases remain incomplete. While high‐resolution LC–MS profiling enabled the detection of thousands of metabolic features and their integration into GWAS and multiomics analyses, definitive structural characterization of many unknown compounds will require complementary approaches such as metabolite purification, nuclear magnetic resonance (NMR) spectroscopy, stable isotope tracing, and targeted MS/MS validation [62, 63]. Importantly, the integration of unknown metabolites with genomic loci, transcriptomic signatures, and co‐expression networks provides a foundation for future functional metabolomics studies. Such approaches may uncover previously undescribed biosynthetic pathways, regulatory modules, and metabolite functions associated with stress adaptation, defense, and nutritional quality.

We classified quinoa accessions as bitter or sweet based on a discriminant compound eluting at 4.68 min. This feature is likely to belong to the flavonoid class rather than to saponins; however, its exact chemical identity and direct contribution to taste perception remain to be fully resolved. While saponins have long been considered the primary determinants of quinoa bitterness, recent studies have suggested that other compound classes, including flavonoids and polyphenols, may also contribute to bitter taste perception in quinoa [50]. In addition, growing evidence shows that di‐ and oligopeptides can elicit bitter or umami sensations [64, 65], further supporting that quinoa taste is likely shaped by a complex interplay of multiple metabolite classes rather than by saponins alone. These findings underscore that breeding strategies aimed at reducing bitterness should take flavonoid, polyphenol, and dipeptide composition, alongside saponin content, into account.

Consistent with previous analyses of quinoa leaf drought responses [66], we observed a significant downregulation of trehalose in roots under drought stress considering all accessions (Figure S25). Glucose, however, displayed contrasting dynamics, showing leaf‐specific upregulation in our study while being downregulated in variety Dianli 129, indicating context‐dependent sugar signaling roles in stress responses. These divergent patterns underscore the pleiotropic functions of sugars in drought signaling and their integration within broader metabolic regulatory networks. Notably, seed metabolism remained comparatively stable, in agreement with findings in other species [67], although recent evidence in quinoa highlights cultivar‐specific transcriptional and metabolic shifts in sugar metabolism under drought [68]. The subdued metabolic response in seeds cannot be attributed to insufficient drought exposure (applied for two weeks, interrupted for two weeks, and then resumed until harvest), but instead may indicate metabolic buffering, developmental canalization, or delayed stress signaling in this tissue. Across all tissues, however, clear genotype‐specific differences were observed at the metabolome, proteome, and transcriptome levels. Rehydration experiments would further help to resolve recovery dynamics and potential longer‐term stress‐associated “memory” effects in metabolism, which were not addressed in the present study.

Our study provides a comprehensive view of the drought regulatory network in quinoa, integrating multi‐omic and multitissue data from three diverse varieties. This approach not only revealed novel metabolites and gene candidates underpinning drought tolerance but also highlights the power of combining genomic, transcriptomic, proteomic, and metabolomic datasets to dissect complex traits. While the primary aim here was to support candidate gene identification in the GWAS framework, these results lay a foundation for future functional studies and for breeding quinoa varieties with enhanced resilience to environmental stress.

### 3.2 GWAS Identifies QTL Linked to Adaptation, Secondary Metabolism, and Seed Pigmentation

Among QTL associated with elevation (Figure S10) – previously shown to explain PC1 variation in a subset of 296 genotypes [15] – candidate genes included amongst the 35 identified genes *MAP1A*, *Arginase 2*, an ERF transcription factor, and *EER5*, all involved in hypoxic stress responses crucial for high‐altitude adaptation in Andean crops like quinoa [54, 55, 69]. Notably, a cluster of 21 SAUR‐like auxin‐responsive genes – implicated in plant development and hypoxia responses [70, 71] – was also linked to elevation. This aligns with known altitude‐related growth constraints such as reduced CO_2_ partial pressure and increased UV radiation [72, 73].

The saponin regulators *TSARL1* and *TSARL2* (on chromosome B5), previously identified by Jarvis et al. [1], were validated in this study using the LC–MS. GWAS of metabolite levels identified a set of candidate genes with direct functional relevance to secondary metabolism, including 26 UGTs, 20 transporters, 34 cytochrome P450s, 34 transcription factors (including nine bHLH), and 23 sugar‐, methyl‐, or acetyltransferases. These gene families collectively mediate biosynthetic tailoring, chemical modification (Figure 5), transport, and transcriptional control of specialized metabolites [51], providing a mechanistic explanation for the observed variation in flavonoid and saponin content. Their association with metabolic traits highlights the power of integrating genomic variation with biochemical phenotypes to uncover regulatory architecture.

Four candidate genes were functionally implicated in the saponin biosynthetic pathway (Figure 5), including CYP88D6 and CYP72A154, previously characterized in serijanate glucuronopyranoside (also known as glycyrrhizin) biosynthesis [74, 75, 76], as well as a SGT as glycosyltransferase responsible for converting soyasapogenol B to soyasaponin III [77]. Serijanate glucuronopyranoside, widely used as a natural sweetener, also exhibits anti‐inflammatory properties [78]. Moreover, group B saponins such as soyasaponin I, which lack the C22 moiety present in group A saponins, are less bitter and considered health‐promoting [44]. While soyasaponins have been considered Fabaceae‐specific [44], our results suggest that structurally similar group B saponins may also occur in Amaranthaceae, highlighting the need for further structural and functional characterization.

Flavonoids and betalains are key specialized metabolites in quinoa with important ecological and nutritional functions. Flavonoids contribute to UV protection, defense, and pollinator attraction [51, 52], yet their biosynthesis and modification remain poorly explored in *C. quinoa* [79, 80]. Among the 21 candidate genes implemented in the flavonoid pathway in our study (Figure 4), UGT91C1 mediates the glycosylation of a kaempferol derivative, a modification that enhances solubility and stability. Notably, UGT91C1 also glycosylates diterpenes such as steviol in rice and *Arabidopsis* [81, 82], highlighting its broad substrate versatility and potential role in modulating diverse secondary metabolites.

Betalains, pigments unique to the Caryophyllales order, provide antioxidant and anticancer benefits [39, 83] and are increasingly valued as natural food colorants. Engineering betalain accumulation in crops has been demonstrated in tomato, potato, and tobacco via targeted expression of biosynthetic genes [40, 84]. Our identification and functional validation of CYP76AD1 in quinoa (Figure 4) provide robust targets for the bioengineering of betalain‐enriched quinoa varieties, opening opportunities to enhance both nutritional quality and market value of this increasingly popular crop.

While these results support the involvement of the candidate genes in saponin‐, flavonoid‐, and betalain‐associated metabolism, transient overexpression does not directly resolve endogenous gene function or regulatory context. Notably, the broader metabolic changes observed beyond the expected pathways may reflect metabolic network interconnectivity, shared precursor usage, or indirect perturbations of downstream metabolism rather than direct enzymatic effects on multiple compound classes.

Integration of the multi‐Theomic drought‐response analyses (Figure 6) identified additional candidate genes through correlation‐based network approaches, thereby providing a framework for the systematic discovery of metabolic genes and their co‐regulators associated with stress resilience. Although the drought experiment was primarily designed to contextualize GWAS‐derived candidate genes under environmentally relevant conditions, the resulting multiomics network further suggests that drought adaptation in quinoa is mediated by coordinated interactions between specialized metabolism, transcriptional regulation, and central stress‐response pathways. Beyond the candidate genes highlighted here, the integrated metabolomic, transcriptomic, proteomic, and lipidomic datasets represent a valuable resource for future studies aimed at dissecting the molecular basis of drought adaptation and stress resilience in quinoa.

Overall, our findings provide a roadmap for breeding programs aimed at improving nutritional quality, stress tolerance, and palatability in quinoa. The identified QTL and candidate genes provide a foundation for marker‐assisted selection and future genomics‐assisted breeding approaches targeting specialized metabolite composition and drought‐responsive traits. By linking metabolic phenotypes to their underlying genetic architecture, the presented metabolome map may facilitate earlier and more precise selection of breeding material, thereby contributing to the development of nutrient‐rich and stress‐resilient cultivars while potentially accelerating breeding cycles. More broadly, this study demonstrates the utility of integrating genomic, metabolomic, transcriptomic, and proteomic approaches to dissect complex traits in an orphan crop with high agronomic and ecological relevance.

## 4 Conclusion

The quinoa diversity panel enabled the identification of 219 candidate genes across 58 high‐confidence QTL, while also providing a scalable framework for uncovering additional loci linked to the secondary metabolome in leaves, roots, and seeds. By integrating multimethod GWAS with stringent SNP selection, we prioritized robust candidates, yet variants detected by fewer methods may reveal additional biologically relevant associations. The integration of metabolomics, genomics, transcriptomics, and proteomics further enabled the identification of candidate genes associated with saponin, betalain, and flavonoid metabolism, while providing insight into the complex regulatory networks underlying metabolic diversity and drought‐responsive processes in quinoa. In addition, our findings suggest that quinoa bitterness may involve contributions from multiple metabolite classes, including flavonoids, polyphenols, dipeptides, and saponins, highlighting the complexity of taste‐associated metabolic traits. By releasing the full mapping data for numerous unknown metabolites – including mass‐to‐charge ratios (m/z) and retention times – we furthermore enable researchers to perform their own annotations and discover QTL for metabolites not captured in our study. This dataset therefore functions both as a curated candidate‐gene resource and a flexible toolkit for expanding the genetic and metabolic understanding of quinoa.

## 5 Material and Methods

### 5.1 Plant Materials, Sequencing, and SNP Identification

Seeds of 603 *C. quinoa* accessions were provided by Prof. Mark Tester from the King Abdullah University of Science and Technology (KAUST). The resequenced 603 accessions were used in study (Stanschewski et al. [85]) of which 296 accessions were previously published [15]. Whole‐genome sequencing (WGS) was performed following [1]. In total, 13 743 142 polymorphic sites were identified by SNP calling following https://github.com/IBEXCluster/Wheat‐SNPCaller [86]. After filtering using vcftools (https://vcftools.github.io/man_latest.html) eliminating SNPs with 90% of missing data, considering 5% minor allele frequency and 30 as quality score threshold 1 450 189 high confidence SNPs remained and used to conduct the genomic and GWAS analysis on the full 603 accessions. For the GWAS of quinoa leaf and root samples, triplicates of 166 accessions were germinated using 0.1% agarose containing 500 µm gibberellic acid A_3_ and stratified for two days at 4°C. Plants were grown using sandy soil and 1% NovaTec classic fertilizer in the greenhouse and leaf and root tissues were harvested after four weeks of growth.

### 5.2 Drought Experiment: Growth Conditions and Sampling

Seeds were surface sterilized following the protocol of Hesami et al. [87]. Briefly, seeds were incubated in 70% ethanol for 15 s, rinsed with double‐distilled water (ddH_2_O), and then treated for 5 min with 20% sodium hypochlorite containing 0.001% Tween‐20. After four washes with ddH_2_O, seeds were stratified for two days at 4°C in 0.1% agarose supplemented with 500 µm gibberellic acid A_3_. Seedlings were grown in sandy soil supplemented with 1% NovaTec Classic fertilizer under controlled conditions in both the polytunnel and greenhouse starting in May. The following quinoa accessions were grown in the polytunnel: Ames‐13760, PI‐665276, LM2, CHEN‐199, CHEN‐384, and D‐12393. In the greenhouse, the accessions CHEN‐199, D‐12165, Ames‐13760, CHEN‐384, PI‐665276, and LM2 were cultivated. Drought stress was applied by modifying the irrigation regime: in the polytunnel, plants were watered every second day instead of daily; in the greenhouse, the amount of water was reduced by half every other day. Irrigation volumes were adjusted based on the full turgor of control plants. After two weeks of drought stress, leaf and root samples were harvested from quinoa accessions in the greenhouse at five weeks of age. At the same time, fresh and dry weight of the above‐ground tissue was recorded. Leaf samples from polytunnel‐grown plants were harvested at the same developmental stage. Photosynthetic parameters were recorded using the MultispeQ device (PhotosynQ), and plant height was measured. Drought stress was then paused for two weeks and subsequently reapplied. Plants were then grown to maturity under continued drought conditions, and final measurements of plant height and dry weight were taken.

### 5.3 Genomic Analysis

For the analysis of the genomic diversity Tassel 5 [88] and vcftools [89] was used. Tajima's D was calculated in a 100 kb sliding window and the plot was designed using the R package ‘CMplot’. The phylogenetic tree was designed using iTOL (https://itol.embl.de/). The population structure of the GWAS population was determined using the software fastSTRUCTURE v1.0 [90] was utilized with *K* 1 – 15. The optimal *K* value was determined by fastSTRUCTUREs chookeK.py implementation and visualized with the R package ‘pophelperShiny’.

### 5.4 Metabolite Profiling Using Ultra‐High‐Pressure Liquid Chromatography (UHPLC)–MS

Metabolites were extracted using a methanol–chloroform–water biphasic protocol based on [91] and adapted for plant tissues [92]. In brief, 50 mg of dried seeds were ground for 1 min at 23 Hz, after addition of 700 µL of 100% methanol spiked with 0.4 µg/mL isovitexin, the material was ground for another minute at 17 Hz, shaken for 10 min at 28°C, sonicated for 5 min and 500 µL of chloroform (containing 0.5 µg/mL phosphocholine 17:0) was added before adding 700 µL of ddH_2_O. The extract was centrifuged for 5 min at 10 000 *g* and 100 µL of the semipolar phase was transferred to LC–MS vials for analysis. For lipid analysis 300 µL of the apolar phase was concentrated and resuspended in 200 µL acetonitrile: isopropanol [7:3]. After vortexing the samples were ultrasonicated for 2 or 5 min for polar and lipid extracts, respectively, centrifuged (5 min, room temperature, 10 000 *g*) and 100 µL of the supernatant was used for ultra‐high‐pressure liquid chromatography electrospray ionization mass spectrometry (UHPLC–ESI–MS).

Analysis of semipolar metabolites was conducted on a UHPLC–ESI–MS machine as described previously [93]. For semipolar compounds a technical injection was performed in order to exclude machine errors. In brief, the UHPLC system was equipped with an HSS T3 C18 reverse‐phase column (100× 2.1 mm internal diameter, 1.8 µm particle size; waters) that was operated at a temperature of 40°C. The mobile phases consisted of either 0.1% formic acid in water (solvent A) and 0.1% formic acid in acetonitrile (solvent B) for semipolar compounds and of 1% 1 m NH_4_‐acetate, 0.1% acetic acid in UHPLC grade water (solvent A) and 1% 1 m NH_4_‐acetate, 0.1% acetic acid in UHPLC grade acetonitrile:isopropanol [7:3] (solvent B) for the lipophilic fraction. The flow rate of the mobile phase was 400 µL min^−1^, and 3 µL of the sample was loaded per injection. The UHPLC instrument was connected to an Exactive Orbitrap focus (Thermo Fisher Scientific) via a heated electrospray source (Thermo Fisher Scientific). The spectra were recorded using full‐scan in positive ion‐detection mode, covering a mass range from m/z 100 to 1500 with an ESI approach (capillary conditions at 3 kV, 200°C; drying gas at 350°C; sheet and auxiliary gas flow at 60 and 20 U, respectively; skimmer and tube lens at 25 and 130 V, respectively) [94].The resolution was set to 70 000 and the maximum scan time was set to 250 ms. The sheath gas was set to a value of 60 while the auxiliary gas was set to 35. The transfer capillary temperature was set to 150°C while the heater temperature was adjusted to 300°C. The spray voltage was fixed at 3 kV, with a capillary voltage and a skimmer voltage of 25 and 15 V, respectively. MS spectra were recorded from 0 to 19 min of the UPLC gradient. Processing of chromatograms, peak detection, and integration was performed using RefinerMS (version 5.3; GeneData).

### 5.5 Metabolite Profiling Using GC–MS

Primary metabolites were extracted as described above. An aliquot of 100 µL of the polar phase was dried in a SpeedVac concentrator and stored at −20°C. For derivatization of the primary metabolites, 55 µL of 30 mg mL^−1^ methoxyaminhydrochloride in pyridine was added to the concentrate and incubated (120 min, 37°C, 950 rpm). Following, 110 µL *N*‐Methyl‐*N*‐(trimethylsilyl) trifluoracetamid (MSTFA) was added, shaken (30 min, 37°C, 950 rpm) and 100 µL was used for GC–MS analysis. GC–MS was performed according to [95]. Separation took place by injection of 1 µL of samples in a splitless mode with helium as carrier gas with a flow set to 2 mL min^−1^ (constant with electronic pressure control) by usage of an autosampler setup. For high‐abundance metabolites (glucose, fructose, saccharose, malic acid, glutamic acid), injection was executed in a split mode with a 1:20 ratio. For metabolite profiling, a 30 m MDN‐35 capillary column with an isothermal temperature program (2 min at 80°C, 15°C per min increase to 330°C and 6 min at 330°C, followed by rapid cooling; transfer line temperature at 250°C matching ion source conditions). The instrument recorded 20 scans per s in a mass range of m/z 70 to 600 (remaining chromatography with a 170 s solvent delay with filaments turned off). The manual mass defect was set to 0, and detector voltage to 1700–1850 V with a filament bias current at −70 V. Processing of chromatograms, peak detection, and integration was performed using RefinerMS (version 5.3; GeneData).

### 5.6 Compound Annotation, Filtering, Normalization, and Transformation

Metabolite identification and annotation were performed using in‐house standard compounds, tandem MS (MS/MS) fragmentation, and metabolomics databases. When using the in‐house reference compound library, we allowed for a 5‐ppm mass error and a dynamic retention‐time shift of 0.1. Additionally, we conducted a literature and integrated metabolic data analysis from previous studies, for example, betalain annotation from Dini et al. [96], Xie and Chen [97], Escribano et al. [83]; saponin annotation from Madl et al. [98], Tabatabaei et al. [48], Escribano et al. [83]; and flavonoids based on Wang et al. [51]. Metabolite data is reported following updated standards for metabolite reporting [99].

Data were filtered removing features with quality controls (QCs) that contain the pooled samples >5%, the coefficient of variation of QCs >60%, missing values of samples >80%, and the signal/blank ratio <10%. Only features from 1–17 min were used. Data were imputed by 10% of the minimum value of the trait before normalization based on the sample weight, the internal standard isovitexin, the feature mean, the QCs per batch and a day mean standardization. For GWAS, samples were log_2_ transformed.

### 5.7 Metabolite Network Analysis

Metabolite networks were constructed from normalized metabolite abundances across accessions, with compounds annotated by tissue and class. Pairwise Pearson correlations between metabolites were calculated, and edges were retained for correlations |*r*| > 0.7 with *p* < 0.05. Networks were built using ‘igraph’, with node degree calculated to identify hub metabolites. Networks were visualized using ‘ggraph’ with the Kamada–Kawai layout. All analyses were performed in R (4.3.2.) using the ‘tidyverse’, ‘igraph’, ‘ggraph’, and ‘viridis’ packages.

### 5.8 GWAS Analysis

To screen for significant associations using GWAS the R package ‘rMVP’ [100] was used employing HE regression as variance component method and as method for genotype binning, FaST‐LMM was utilized. To correct for population structure effects with a kinship matrix and three PCs as fixed effects. Single‐locus MLM was used as an association testing method for the identification of significant associations between genetic variants (SNPs) and the phenotype of interest. Missing values were excluded from the GWAS. We used the Bonferroni correction to adjust the significance threshold for multiple comparisons. Significant compounds were reanalyzed using efficient mixed‐model association (EMMA) as variance component and binning method, and MLM and farmCPU for association testing method. Candidate genes were identified by scanning the genetic area ± 100 kb of the lead SNP and determining the biological relevance by including the results of OrthoFinder [101, 102]. Protein sequences were obtained from the Joint Genome Institute (https://data.jgi.doe.gov/; *A. thaliana* – TAIR10, *Spinacia oleracea* – Spov3, *Amaranthus hypochondriacus* – v2.1, *Arachis hypohaea* cv. Tifrunner, *B. vulgaris* – EL10_1.0, *Cicer arietinum* – v1.0, *C. quinoa –* v1.0, *Chlamydomonas reinhardtii* – v5.6, *Crocus sativus* – v1.0, *Gossypium hirsutum* – v2.1, *G. max* – Wm82.a2.v1, *Lupinus albus* – v1, *Lens culinaris* – v1, *Medicago truncatula* – Mt4.0v1, *Oryza sativa* – v7.0, *Phaseolus vulgaris* – v2.1, *Sorghum bicolor* – v3.1.1, *Solanum lycopersicum* – ITAG4.0, *S. tuberosum* – v6.1, *Zea mays* – V4) and NCBI (https://www.ncbi.nlm.nih.gov/protein/; *S. lycopersicum* – ITAG4.1). In addition, transcriptomic data of previous quinoa studies were integrated for candidate gene identification [66, 103, 104, 105, 106]. Furthermore, the differential abundance of primary and secondary metabolites, lipids, proteins, and RNA during the drought experiment was an additional proxy for the involvement in the secondary metabolome.

Common QTL across tissues were assigned in a ± 50 kb sliding window using ‘IRanges’ and ‘GenomicRanges’. The pleiotropic maps were designed with the circos representation Fuji plot [107]. Common QTL were assigned in a ± 25 kb window following the method previously described [108, 109].

### 5.9 Proteomics

Proteins from seeds, leaves and roots of LM2, CHEN‐384, and PI‐665276 grown under drought and control conditions were extracted using the methyl tert‐butyl ether (MTBE) extraction method [110, 111]. In brief, 50 mg of seed, leaf or root material was incubated for 10 min at 4°C on an orbital shaker after adding 1 mL of −20°C precooled extraction solvent mixture 1 (MTBE/methanol [3:1]). After 10 min of ultrasonication 500 µL of extraction solvent, mixture 2 (methanol/water [3:1]) was added and samples were centrifuged (5 min, 4°C, 11 000 g). The supernatant was removed and the pellets dried at room temperature. Proteins were digested using in‐solution digestion protocol with modifications described in [112, 113]. Briefly, proteins were digested using a trypsin/Lys‐C mixture (Mass Spec Grade; Promega, V5073) according to the manufacturer's instructions. Digested peptides were desalted on C18 SEP‐Pak columns (Teknokroma, TR‐F034000), in which peptides were eluted using centrifuge. The dried peptides were resolubilized and subsequently, analyzed by LC–MS/MS using a 480‐Exploris mass spectrometer coupled to an Ultimate U3000 nano LC (Thermo fisher, Scientific). The gradient and mass spec settings were kept as same as Wagner et al. [114]. Raw data were processed using MaxQuant software [115]. The Hela digest was used before and after the run to monitor the accuracy of the experiment.

### 5.10 Transcriptomics

Total RNA of leaves and roots from the greenhouse and seeds from the polytunnel of LM2, CHEN‐384, and PI‐665276 grown under drought and control conditions was extracted using NucleoSpin RNA plant kit (Macherey–Nagel). RNA sequencing was performed by Novogene (Munich, Germany). Total RNA quality was assessed prior to library preparation. Polyadenylated RNA was enriched from total RNA using poly‐T oligo–attached magnetic beads, fragmented, and reverse‐transcribed using random hexamer primers. Second‐strand cDNA synthesis was performed using dUTP to generate strand‐specific libraries. Libraries were constructed following standard Illumina protocols, including end repair, A‐tailing, adapter ligation, size selection, polymerase chain reaction (PCR) amplification, and purification. Library quality and concentration were assessed using Qubit fluorometry, quantitative PCR, and an Agilent Bioanalyzer. Sequencing was performed on an Illumina NovaSeq X Plus platform using paired‐end sequencing (PE150). Raw reads were processed to remove adapter contamination, reads containing more than 10% ambiguous nucleotides, and reads with low base quality, as previously described [116]. Clean reads were aligned to the reference genome using HISAT2, a graph‐based splice‐aware aligner optimized for RNA‐seq data [117]. Transcript assembly and novel transcript identification were performed using Cufflinks [118]. Gene expression levels were quantified based on mapped reads and normalized as fragments per kilobase of transcript per million mapped reads (FPKM) to account for sequencing depth and gene length [117, 119, 120].

### 5.11 Gene Enrichment Analysis

Gene Ontology (GO) is an acknowledged bioinformatics tool for representing gene product properties across all species through defined GO terms. The functions of genes and their products are represented by GO terms and effectively predicted through GO annotation [121]. Functional enrichment analysis of significant proteins of drought versus control with cut‐off criterion *p*‐value < 0.05 and |log_2_ fold change| ≥ 1 were identified using the database g:Profiler (https://biit.cs.ut.ee/gprofiler/gost) with the gene IDs of *C. quinoa* – v1.0 matched through OrthoFinder.

### 5.12 WGCNA

Co‐expression networks were constructed using the ‘WGCNA’ R package. Multiomics features (metabolites, proteins, transcripts) were clustered based on pairwise correlations, and modules of highly correlated features were identified using hierarchical clustering and dynamic tree cutting. Module eigengenes (first principal component) summarized module expression, and correlations with phenotypic traits were used to assess module significance. Hub features within each module were defined by high module membership (kME) and trait relevance (gene significance, GS). Network visualizations were generated in R using igraph and ggraph, with node color representing omics type and shape indicating tissue. Cytoscape‐compatible node and edge tables were exported for downstream analysis and are available upon request.

### 5.13 Candidate Gene Cloning

Cloning of candidate genes was performed as described in [108]. In brief, the total RNA from leaf, root, and seed tissue of LM2, CHEN‐384, and PI‐665276 was isolated using a NucleoSpin RNA plant kit (Macherey–Nagel) according to the manufacturer's instructions. First‐strand cDNA was synthesized using 1.5 mg RNA and Prime Script RT reagent Kit with gDNA eraser (Takara) according to the manufacturer's instructions. Full‐length cDNA of quinoa seeds was amplified using 250 ng (Table S15). The entry clone was obtained through recombination of the PCR product with pDONR207 (Invitrogen). By LR recombination error‐free clones were introduced into pK7FWG2 [122]. The transformation of three leaves of four *N. benthamiana* plants and five leaves of two *C. quinoa* plants of varieties CHEN‐369 and Cauquenes was performed following [123] and [124] with *Agrobacterium tumefaciens* (AGL1) containing vector pBin61‐p19, infiltrated with an OD600 of 0.5. DM6000B/SP5 confocal laser scanning microscope (Leica Microsystems, Wetzlar, Germany) was used for verification of expression. Metabolic shifts were analyzed after 3 days using 50 mg of agro‐injected leaves and subjected to LC–MS analysis using chloroform:water:methanol extraction [91, 92] as described previously.

### 5.14 Statistical Analysis

The data were normalized using the internal standard, sample weight, the feature mean, QCs per batch and day mean standardization for metabolomics, for proteomics and transcriptomics feature mean normalization was applied and data were log_2_ transformed. Outliers were detected using the interquartile range. Boxplots show median, interquartile range (IQR), and 1.5× IQR whiskers. Heatmaps represent normalized metabolite abundances. PCA plots display the first two principal components with percentage variance explained. Data are shown as counts for barplots. Each point in PCA represents one accession or sample. Each point in scatter plot represents a compound class. Each point in Manhattan plots represent a SNP. For genomic analysis *n*_SNPs_ = 1 450 000, *n*_accessions_ = 603, for metabolomics of the GWAS panel *n*_GWAS‐seed‐metabolites_ = 4706, *n*_GWAS‐leaf‐metabolites_ = 609, *n*_GWAS‐root‐metabolites_ = 1210, *n*_GWAS‐seed‐accessions_ = 573, *n*_GWAS‐leaf‐accessions_ = 166, *n*_GWAS‐root‐accessions_ = 167. For the ridgeline plot of the CV for leaf (*n*_amino_acids_ = 13, *n*_betalains_ = 17, *n*_dipeptides_ = 45, *n*_flavonoids_ = 90, *n*_saponins_ = 20), root (*n*_amino acids_ = 18, *n*_betalains_ = 28, *n*_dipeptides_ = 59, *n*_flavonoids_ = 12, *n*_saponins_ = 166), and seed (*n*_amino acids_ = 17, *n*_betalains_ = 143, *n*_dipeptides_ = 198, *n*_flavonoids_ = 181, *n*_lipids_ = 128, *n*_saponins_ = 95) secondary metabolites and lipids were used. For the heatmap *n*_betalains_ = 18, *n*_dipeptides_ = 7, *n*_flavonoids_ = 38, *n*_saponins_ = 12, *n*_lipids_ = 6, *n*_others_ = 15. Network analysis was conducted using Pearson correlation (|*r*| > 0.7, *p* < 0.05) with *n*_others‐seed_ = 252, *n*_others‐leaf_ = 90, *n*_others‐seeds_ = 92, and the residual metabolites as for the ridgeline plot. For the transient overexpression experiments (PCA and boxplot) *n* = 10 samples were used and for PCA *n*_metabolites‐CYP76AD1_ = 6627, *n*_metabolites‐UGT91C1_ = 6040, *n*_metabolites‐CYP72A154_ = 5100, *n*_metabolites‐SGT_ = 7074. For the PCA of the drought experiment *n* = 5360 secondary metabolites and lipids with *n*_drought_ = 6 and *n*_control_ = 6 samples, *n* = 8575 proteins and *n* = 51,527 transcripts with *n*_drought_ = 3 and *n*_control_ = 3 were used. In order to compute volcano plots between conditions, data were tested for normal distribution and significance was tested by either Student's *t*‐test or Wilcoxon rank‐sum test (***p < 0.001, **p < 0.01, *p < 0.5). Multiple comparison of accessions was performed either by two‐way ANOVA and post hoc Tukey HSD test or Kruskal–Wallis test and post hoc Dunn's test, ‘multcomp’ was utilized for computation of significance letters (*p* < 0.05). All data were analyzed in the R environment version 4.3.2. PCA was performed using ‘ggbiplot’. Heatmaps were designed using ‘pheatmap’ with median normalization and log_2_ transformation using Ward's clustering method. Venn diagrams were computed using the R package ‘VennDiagram’, barplots, boxplots, volcano plots, and histograms were designed using ‘ggplot2’ and ‘viridis’ for color selection.

## Author Contributions

M.T. and S.A. conceptualized the experiment. J.v.S. and S.A. wrote the manuscript with input from all authors. A.R.F. provided guidance on experimental strategy. E.L.R., C.S., N.O.S., and M.A.T. provided the germplasm and the sequencing data. A.K. filtered sequencing data. V.P.T. and A.S. performed proteomic analysis. J.v.S. performed data analysis. J.v.S., R.W., and M.M. performed experiments.

## Conflicts of Interest

The authors declare no conflicts of interest.

## Supporting information




**Supporting File 1**: advs76426‐sup‐0001‐FiguresS1‐S33.pdf.

**Supporting File 2**: advs76426‐sup‐0002‐TablesS1#U2013S15.xlsx.

## Data Availability

Raw sequence data have been deposited in the National Center for Biotechnology Information BioProject database in the NCBI Sequence Read Archive: PRJNA1357073 and PRJNA673789.
