# Supplementary material for: Multi‐tissue Metabolic GWAS and Drought‐Responsive Multi‐omics Reveal the Genetic Basis of the Quinoa Metabolome
Source: Adv Sci (Weinh). 2026 Jul 7:e76426. Online ahead of print. doi: 10.1002/advs.76426 (PMC13339065; doi:10.1002/advs.76426)
Supplement: Supplementary file 1 — Supporting File 1: advs76426‐sup‐0001‐FiguresS1‐S33.pdf. [file ADVS-9999-e76426-s001.pdf]

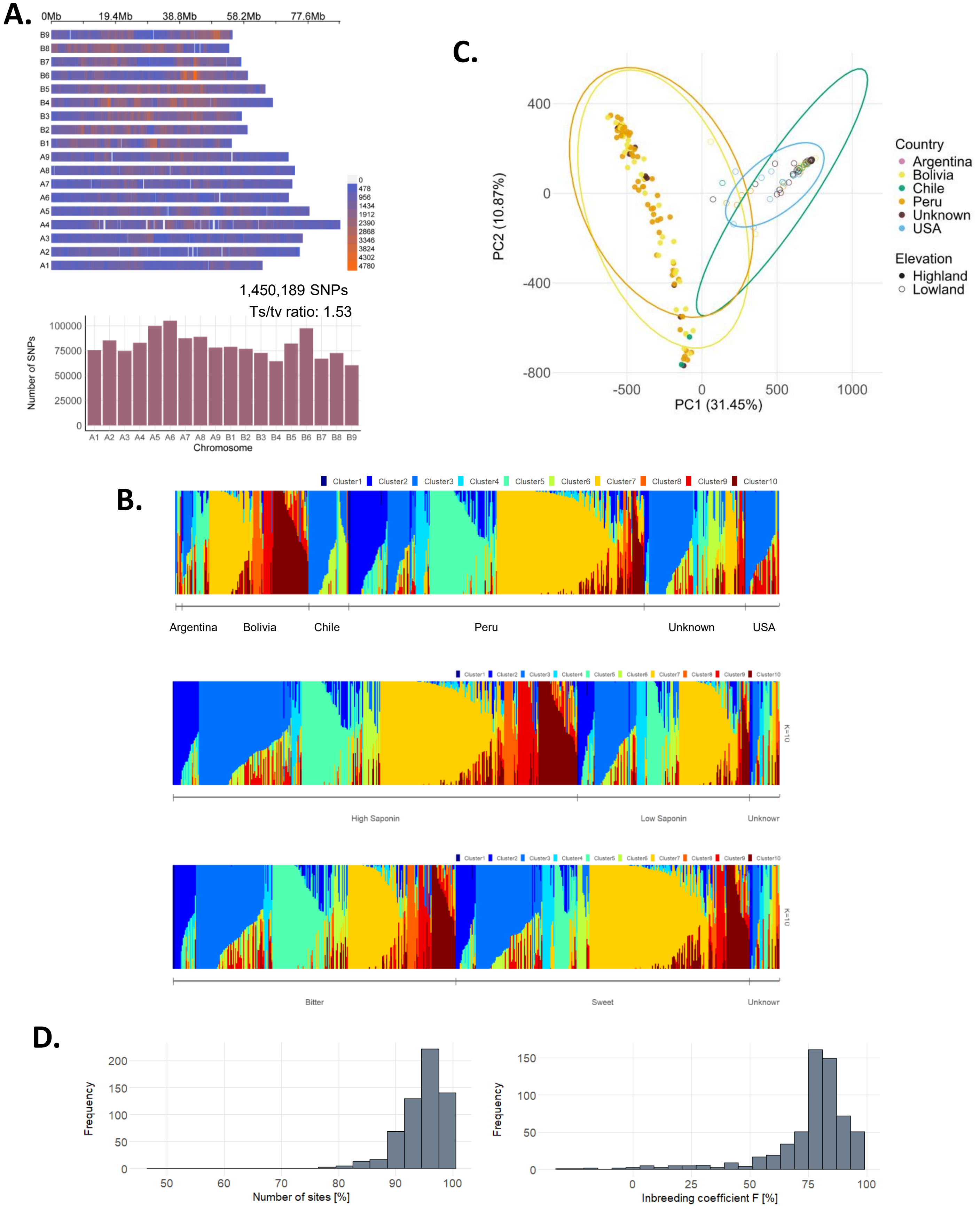

Figure S1. The quinoa diversity panel by its elevation. (**A**) SNP density in 1 Mb non-overlapping bins and number of SNPs per chromosome (60,417–104,938), based on 1,450,189 SNPs with a transition/transversion (Ts/Tv) ratio of 1.53. (**B**) Principal component analysis (PCA) of 166 quinoa accessions used for root and leaf GWAS colored by elevation and shaped by location. (**D**) Proportion of segregating sites and inbreeding coefficient (F [%]) indicate a high degree of inbreeding, consistent with a short breeding history typical of modern crops.

A.

Df	SumOfSqs	R^2	F	Pr(>F)
1	57.305	0.273	217.09	0.001***
Residual	579	152.839	0.727	
Total	580	210.145	1	

B.

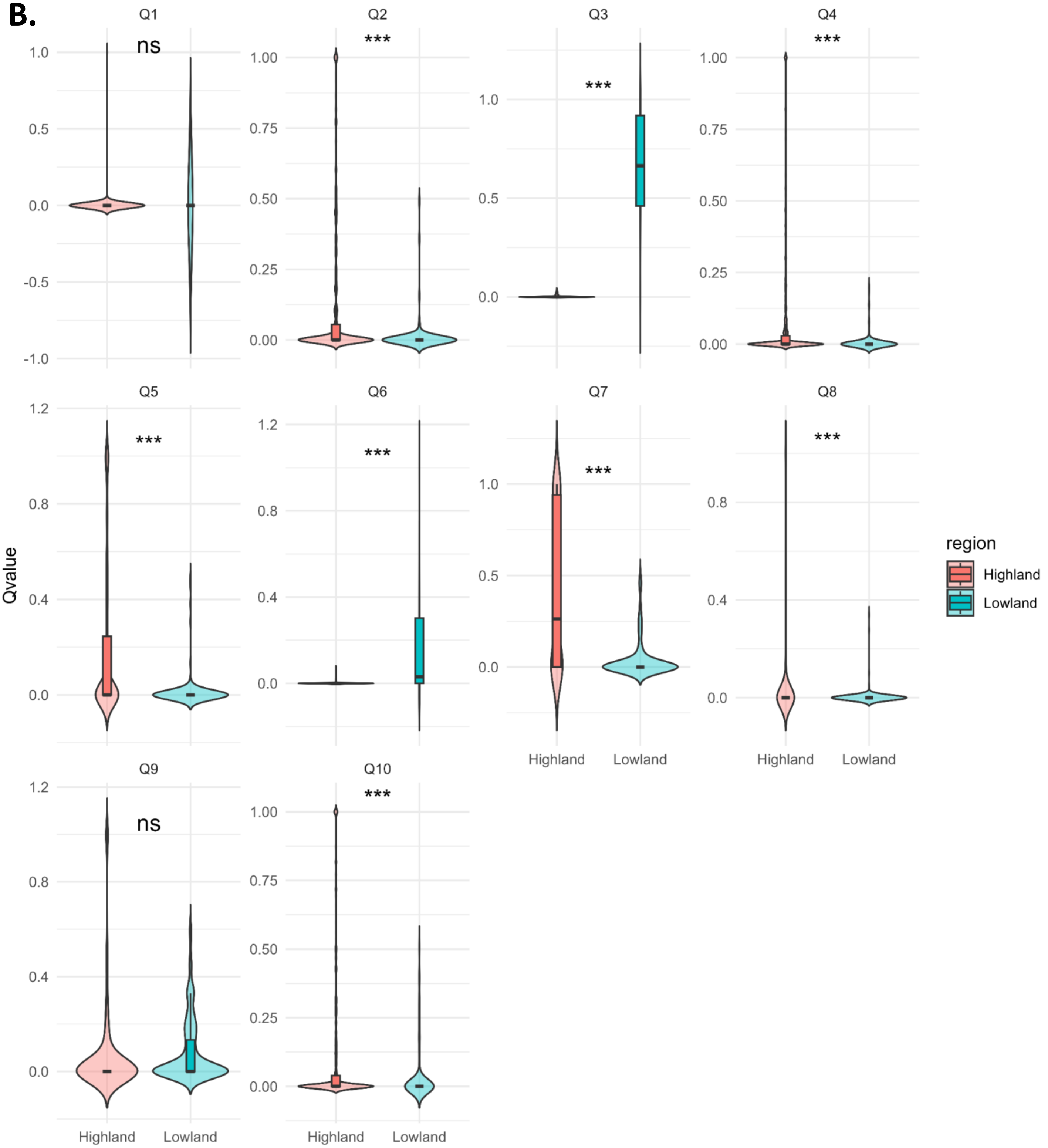

Figure S2. Distribution of ancestry coefficients (fastStructure Q values) across highland and lowland accessions. (A) PERMANOVA result table (adonis2(Qdist_mat ~ region, data = dat)). (B) Each facet represents an inferred ancestral component (K = 10). Violin plots show density distributions, with boxplots indicating median and interquartile ranges. Significances indicate ***p<0.001 (Student's *t*-test).

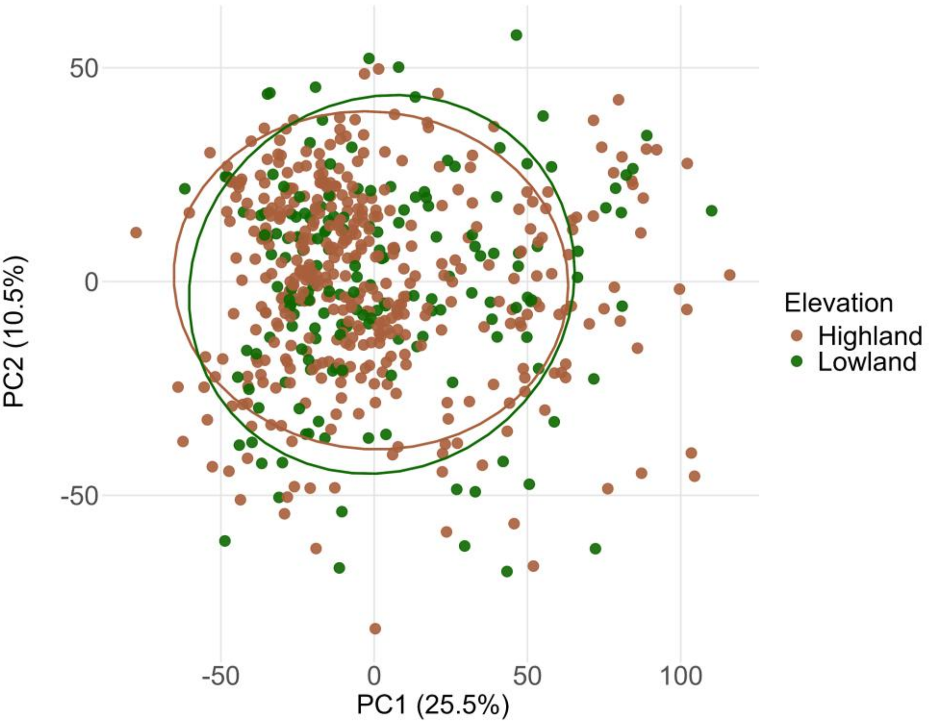

Figure S3. Principal component analysis of apolar seed compounds. 4,949 metabolic features from 581 accessions. Accessions are color-coded by elevation.

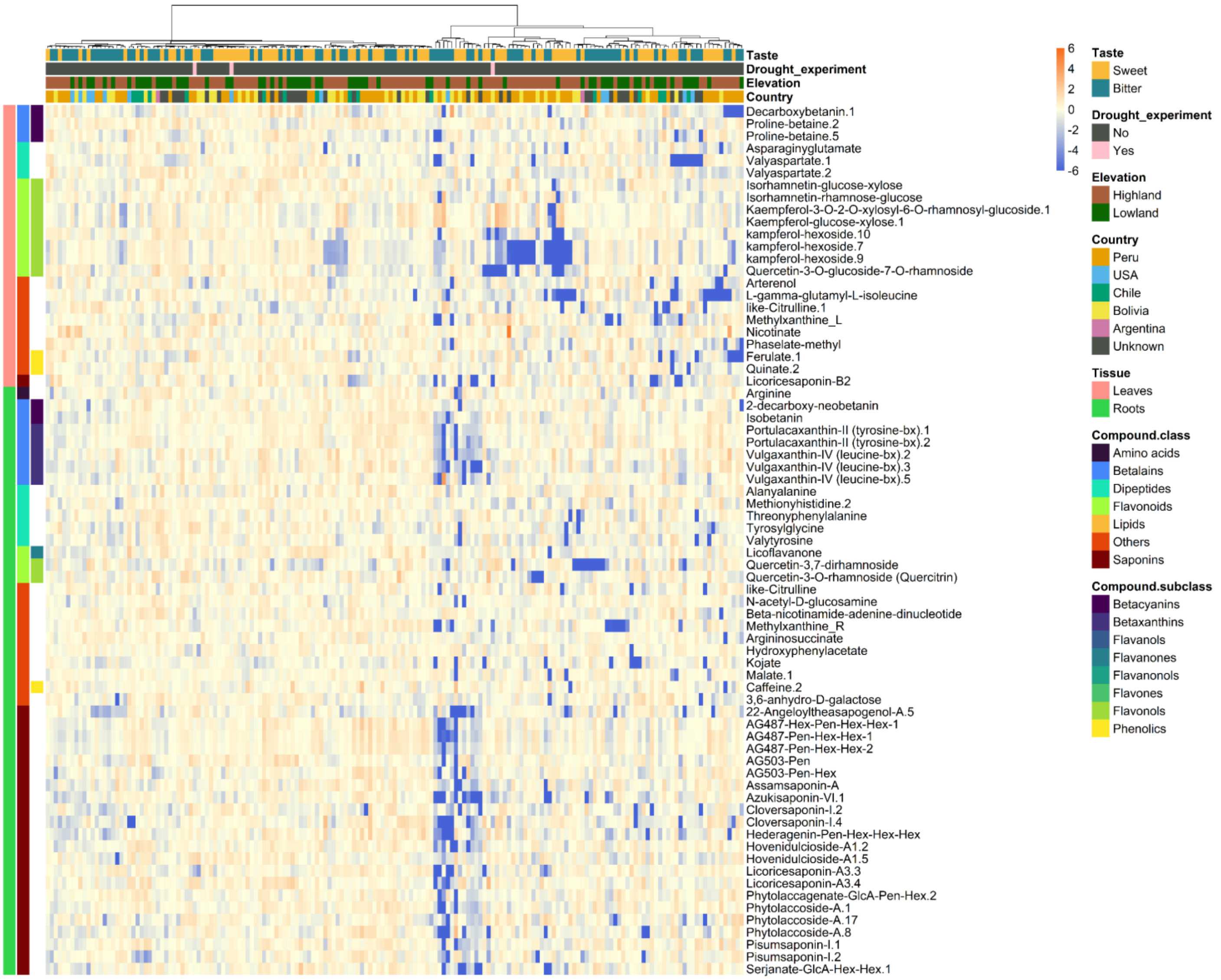

Figure S4. Secondary metabolic diversity of leaf and root tissue. Heatmap of 23 annotated leaf secondary metabolites and 48 root secondary metabolites with a genomic association. For leaves, 3 betalains, 3 dipeptides, 8 flavonoids, 1 saponin, and 8 other compounds, for roots, 1 amino acid, 7 betalains, 5 dipeptides, 3 flavonoids, 23 saponins, and 10 other metabolites. Accessions are color-coded by sweet and bitter taste, location and elevation of origin. Accessions which are used for the drought experiment are marked in pink (from left to right: Ames-13760, D-12393, PI-665276).

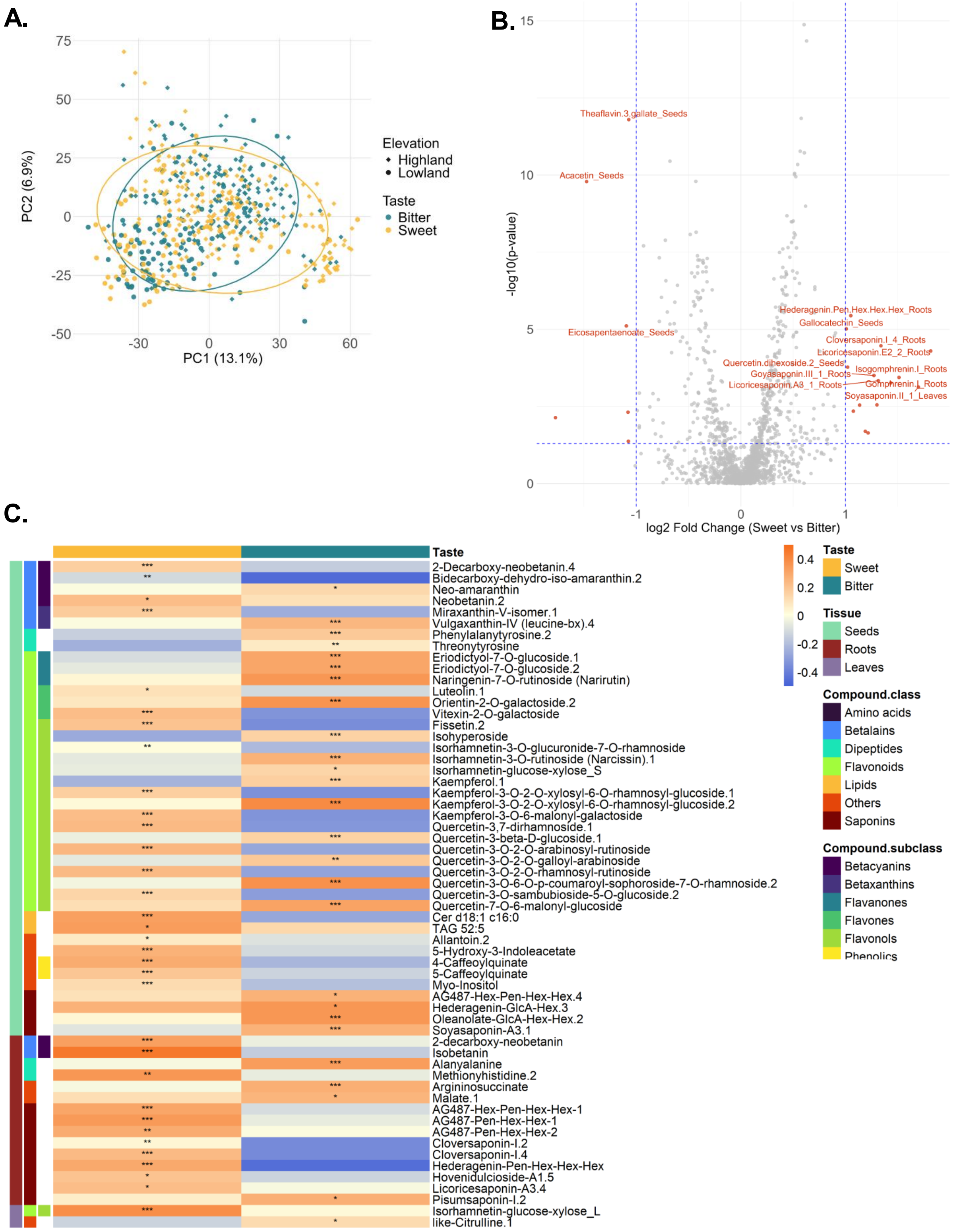

Figure S5. GWAS significant secondary and lipid compounds which correlate with sweet and bitter taste. (**A**) Principal component analysis of secondary metabolites of Fig. 2a grouped by elevation and taste. (**B**) Volcano plot of all annotated secondary metabolic features. Positive $\log_2$ fold change means the trait is higher in sweet accessions. Cut-off: $p < 0.05$, $|\log_2$ fold change$| > 1$. Names are displayed if $p < 0.001$, $|\log_2$ fold change$| > 1$. (**C**) Heatmap of 42 seed secondary and lipid features (6 betalains, 2 dipeptides, 23 flavonoids, 5 others, 4 saponins, 2 lipids), 15 root (2 betalains, 2 dipeptides, 2 others, 9 saponins) and 2 leaf secondary metabolites (1 flavonoid, 1 other) with a GWAS significant association. Significances were determined by either Student's *t*-test or Wilcoxon test based on the data normal distribution (*$p < 0.05$, **$p < 0.01$, ***$p < 0.001$).

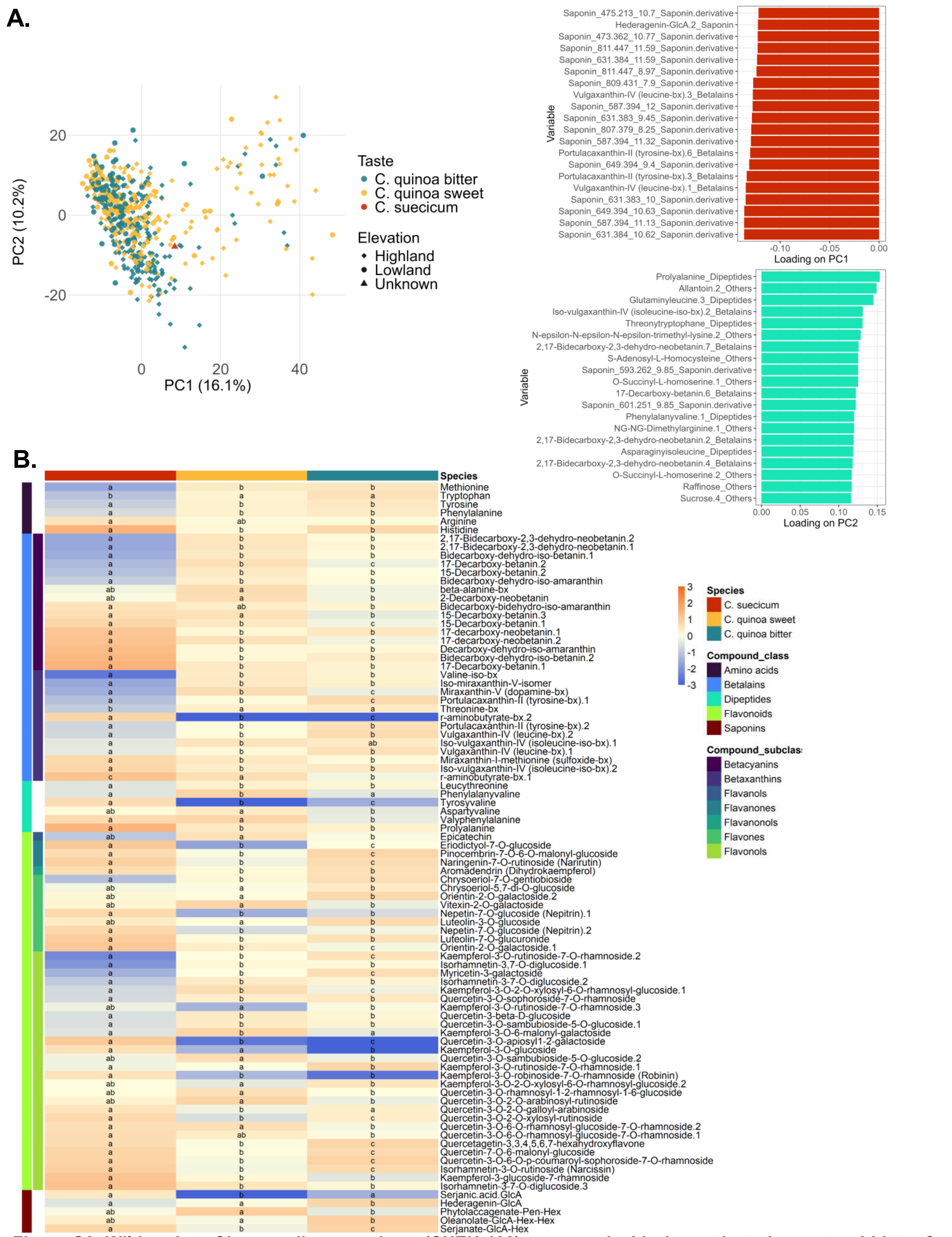

Figure S6. Wild variety *Chenopodium suecicum* (CHEN-100) compared with domesticated sweet and bitter *C. quinoa* varieties. (A) Principal component analysis of 828 common polar compounds (5 saponins, 71 saponin derivatives, 36 betalains, 10 dipeptides, 48 flavonoids, 31 other compounds, and 620 unknown features) and variable contribution plot of PC1 and PC2. (B) Heatmap of 88 compounds (6 amino acids, 29 betalains, 6 dipeptides, 42 flavonoids, 5 saponins) showing a significant difference across *C. suecicum*, and sweet and bitter *C. quinoa*. Letters indicate significances which were determined by either two-way-ANOVA or KRUSKAL-Wallis test based on the data normal distribution.

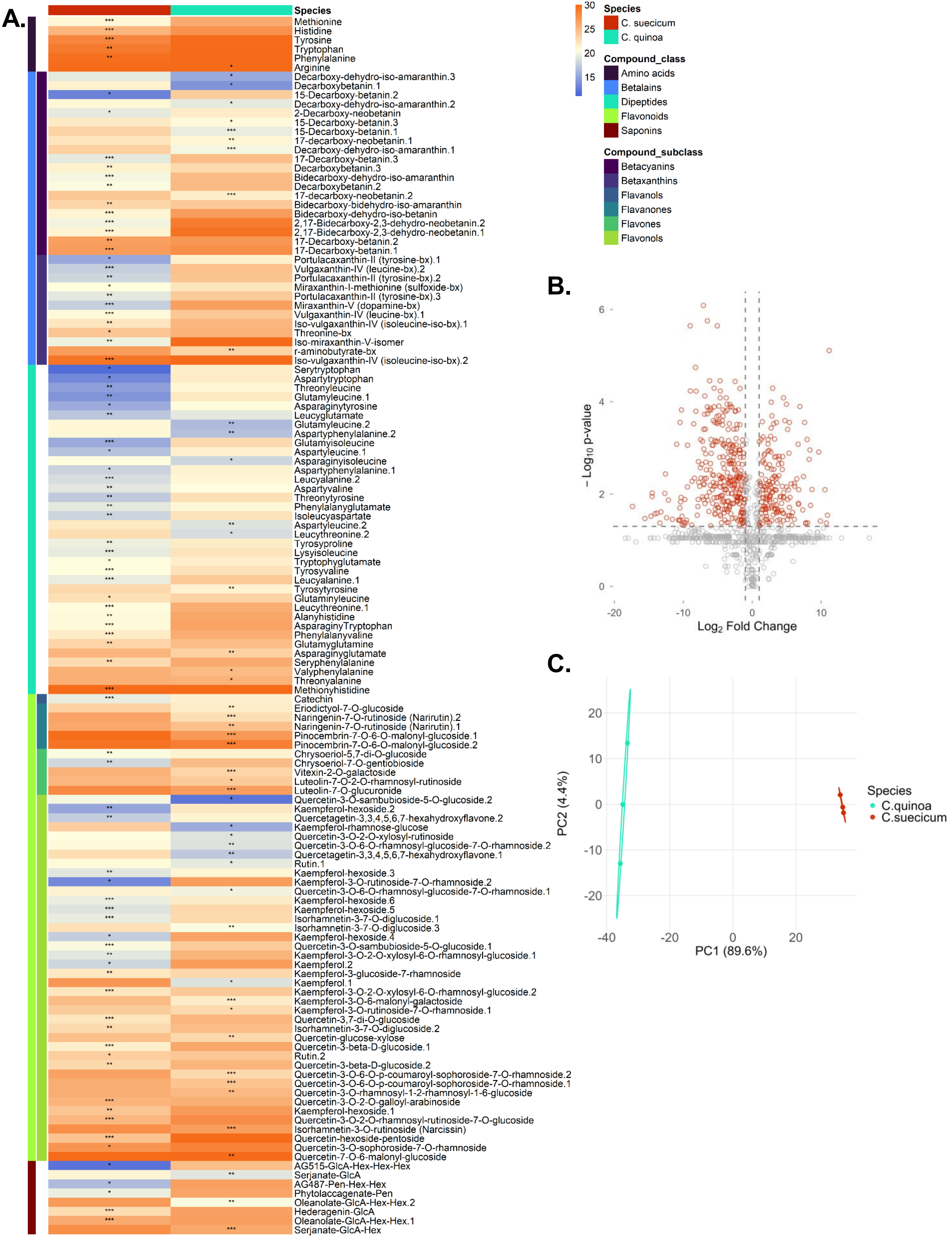

Figure S7. Wild variety *Chenopodium suecicum* (CHEN-100) compared with domesticated *C. quinoa* (CHEN-543). (**A**) Heatmap of 133 annotated secondary metabolites (6 amino acids, 32 betalains, 36 dipeptides, 51 flavonoids, 8 saponins) showing a significant difference across *C. suecicum*, and *C. quinoa*. Asterisks indicate significances which were determined by either Student's *t*-test or Wilcoxon test based on the data normal distribution. (**B**) Volcano plot (cut-off: p-adj < 0.05, $|\log_2 FC| > 1$) of 1465 compounds. (**C**) Principal component analysis of 106 common polar compounds (22 saponins, 8 betalains, 10 dipeptides, 27 flavonoids, and 39 other compounds).

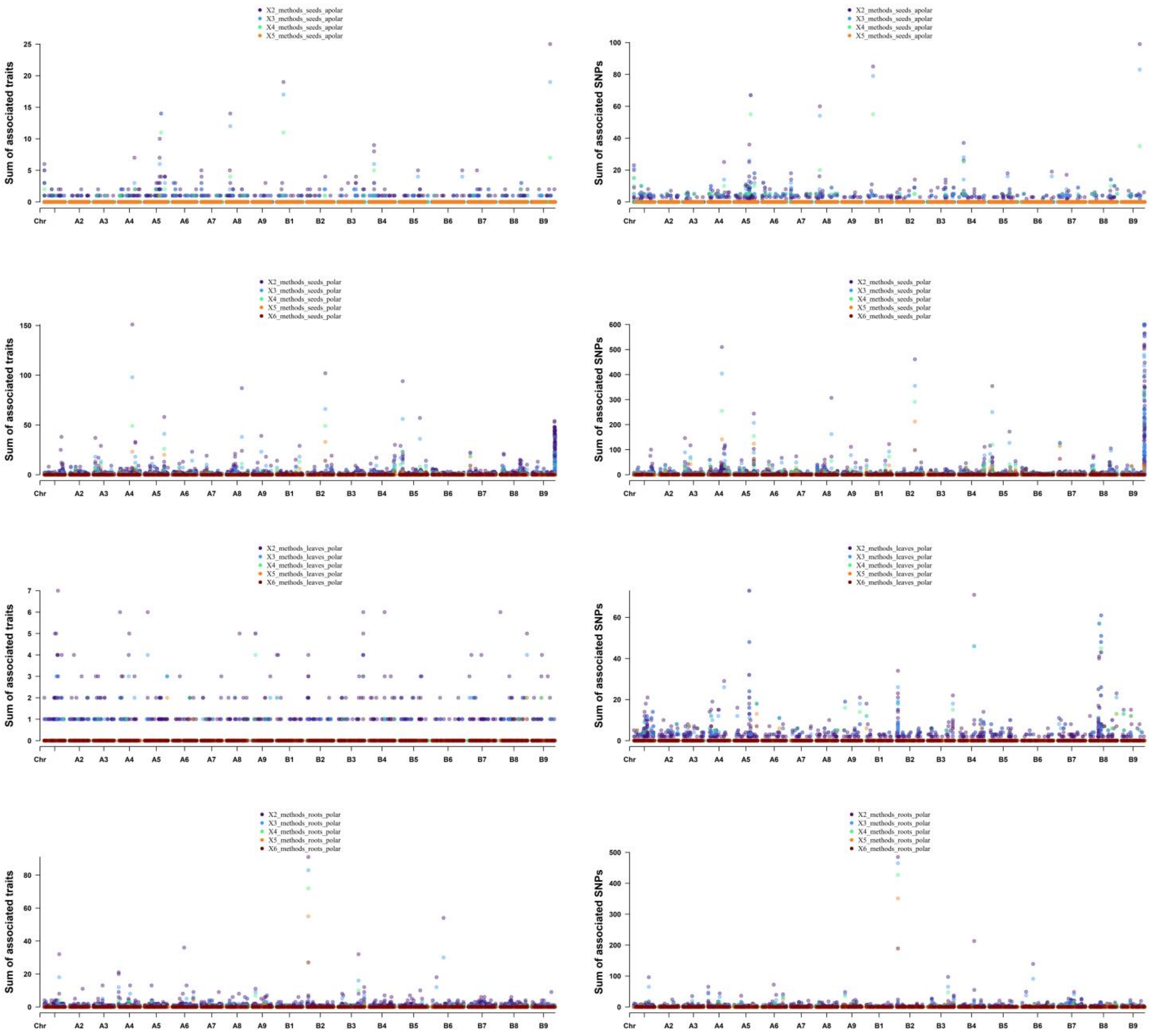

Figure S8. Overlaid Manhattan plots of genome-wide association studies (GWAS) of the total number of associated traits or SNPs for seed apolar and polar metabolites, and leaf and root polar secondary metabolites across six methods. Each dot represents a significant SNP detected by multiple GWAS models (BLINK, FarmCPU, EMMA-MLM, HE-MLM, MLMM, CMLM), colored by the number of methods in which the association was supported: two (purple), three (blue), four (green), five (orange), or six (red). For seed apolar metabolites, 412 associations were detected in ≥ two methods, 287 in ≥ three, 115 in ≥ four, one in ≥ five, and none in all six. For seed polar metabolites, 1,484 associations were detected in ≥ two methods, 1,164 in ≥ three, 624 in ≥ four, 309 in ≥ five, and 99 in all six. In leaves, 178 associations were detected in ≥ two methods, 96 in ≥ three, 44 in ≥ four, 20 in ≥ five, and five in all six. In roots, 381 associations were detected in ≥ two methods, 324 in ≥ three, 209 in ≥ four, 119 in ≥ five, and 46 in all six.

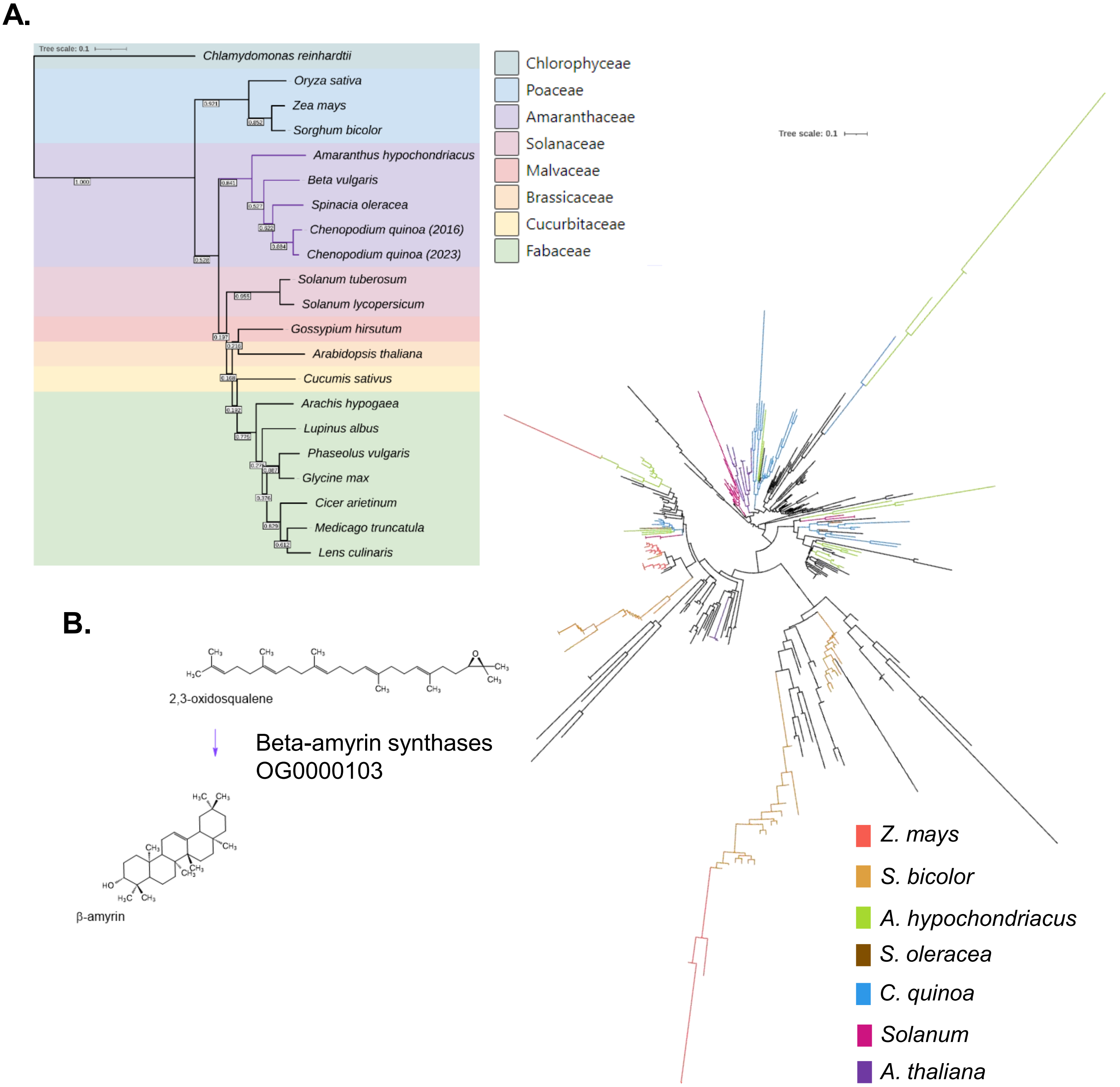

Figure S9. Identification of orthologues genes. OrthoFinder was used to discover orthologues across 20 crop species and eight families (**A**). In total 31,450 orthogroups were detected, OG0000103 containing beta-amyrin synthases serves as an example showing a high degree of duplication and speciation among species allowing conclusions about evolutionary lineages (**B**).

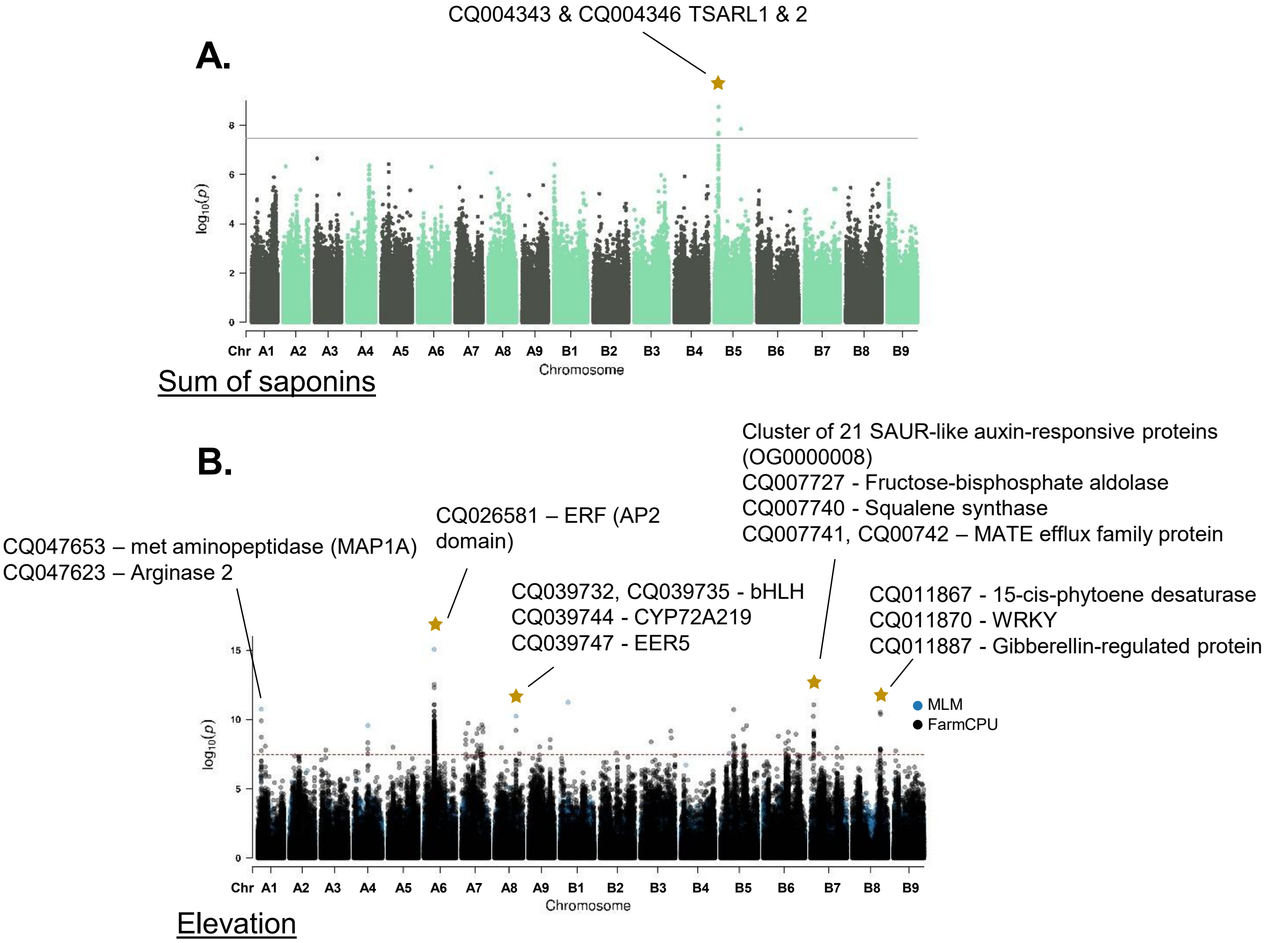

Figure S10. New genome-wide association panel maps to known loci. (**A**) The summed intensities of saponins measured by LC-MS mapped to the previously identified transcription factor triterpene saponin biosynthesis activating regulator 1 (TSARL1) and TSARL2 basic helix-loop-helix (bHLH) by Patirange et al., 2023. (**B**) GWAS results of mapping the elevation reveal four overlapping loci (asterisk) to previously identified loci of the principal component 1 (PC1), separating highland (Type **I**, blue) from lowland (Type **II**, red) accessions by Patirange et al., 2023. Here, five QTL containing 35 genes involved in hypoxia and plant development could be identified using farmCPU and MLM as GWAS models. ERF = ethylene response factor, AP2 = APETALA2, EER = enhanced ethylene response protein, SAUR = small auxin up-regulated RNA. The asterisk indicates similar associations.

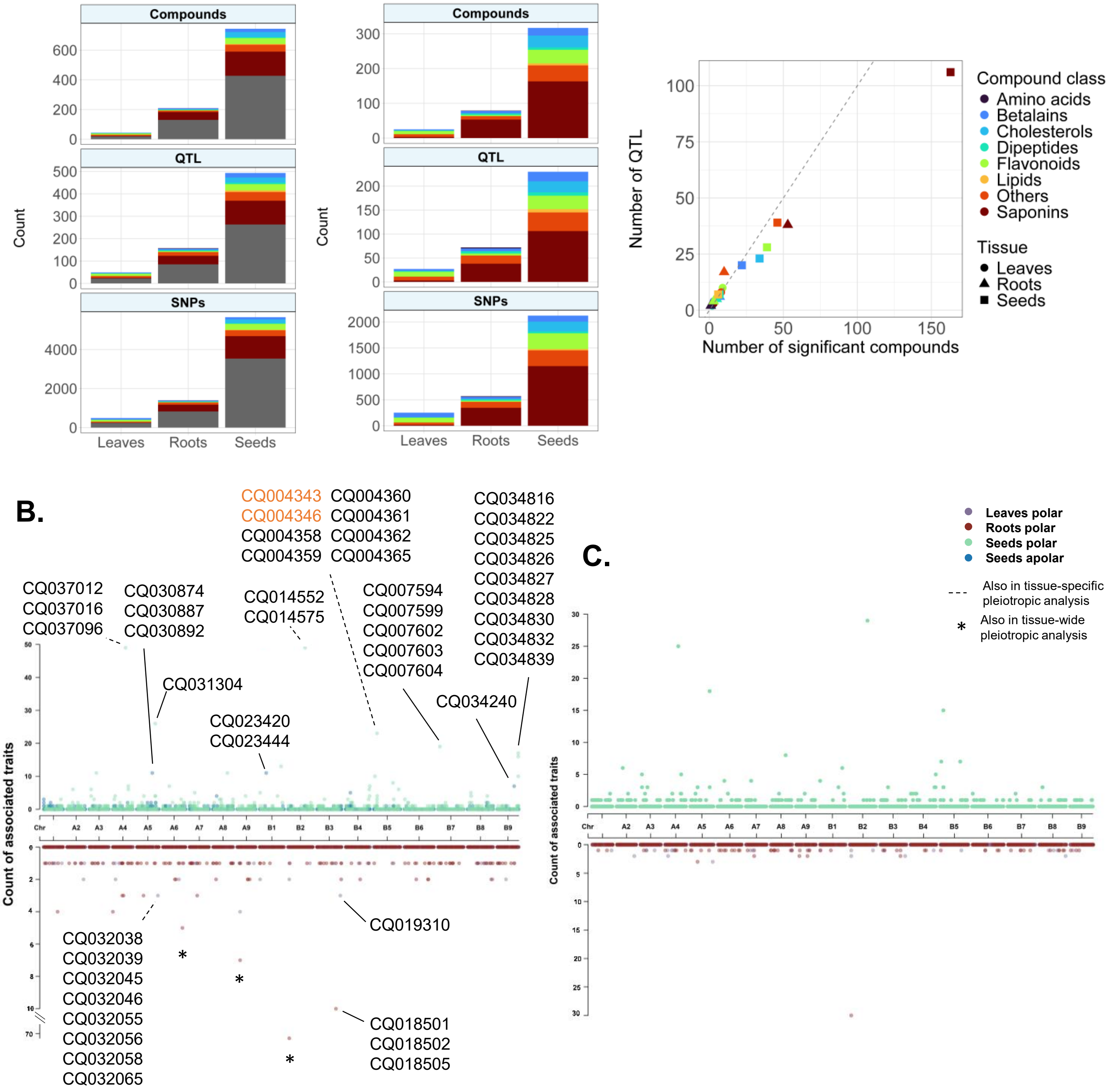

Figure S11. Sum of genomic associated annotated secondary metabolites. (**A**) Stacked bar plot with number of significant compounds, QTL in a 50 kb sliding window and SNPs using four genome-wide association study (GWAS) methods with and without untargeted compounds. Number of identified QTL in a 50 kb sliding window versus number of compounds with a GWAS association grouped by compound class and shaped by tissue without untargeted compounds. (**B**) Count of all metabolic features with significant genomic associations across tissues: 652 seed secondary metabolites, 119 seed lipid features, 44 leaf secondary metabolites, and 209 root secondary metabolites, detected in 573, 166, and 167 quinoa accessions, respectively. In total, 365, 82, 46, and 137 marker–trait associations (MTAs) were identified for seed secondary metabolites, seed lipids, and leaf and root secondary metabolites, respectively. Candidate genes are highlighted. (**C**) Count of annotated 317, 25 and 79 metabolic features of seed secondary metabolic and lipid features (upper), leaf and root secondary metabolic features (lower) of 573, 166 and 167 quinoa accessions, respectively, with a genomic association. In total 204, 26, 66 marker-trait association were detected for seed secondary metabolites and lipids, and leaf and root secondary metabolites.

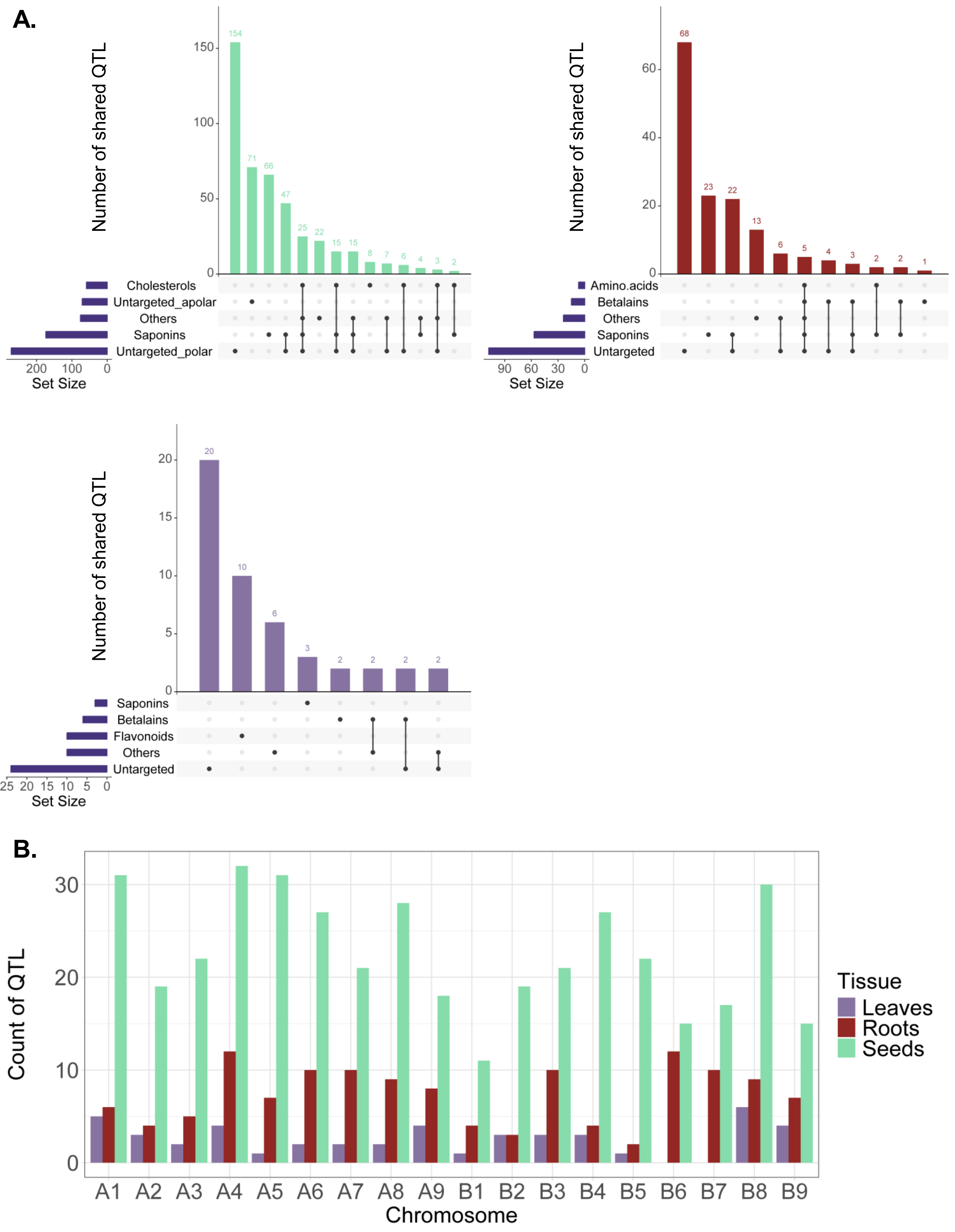

Figure S12. Total number of QTL in seeds, roots and leaves. (**A**) Intersections of QTL in a 50 kb sliding window in seeds (green), roots (red), and leaves (purple). In total 493, 157, and 49 QTL with 148, 46, and 6 shared QTL could be detected across compound classes for seeds, roots and leaves, respectively. (**B**) Count of QTL per chromosome of leaves, roots and seeds.

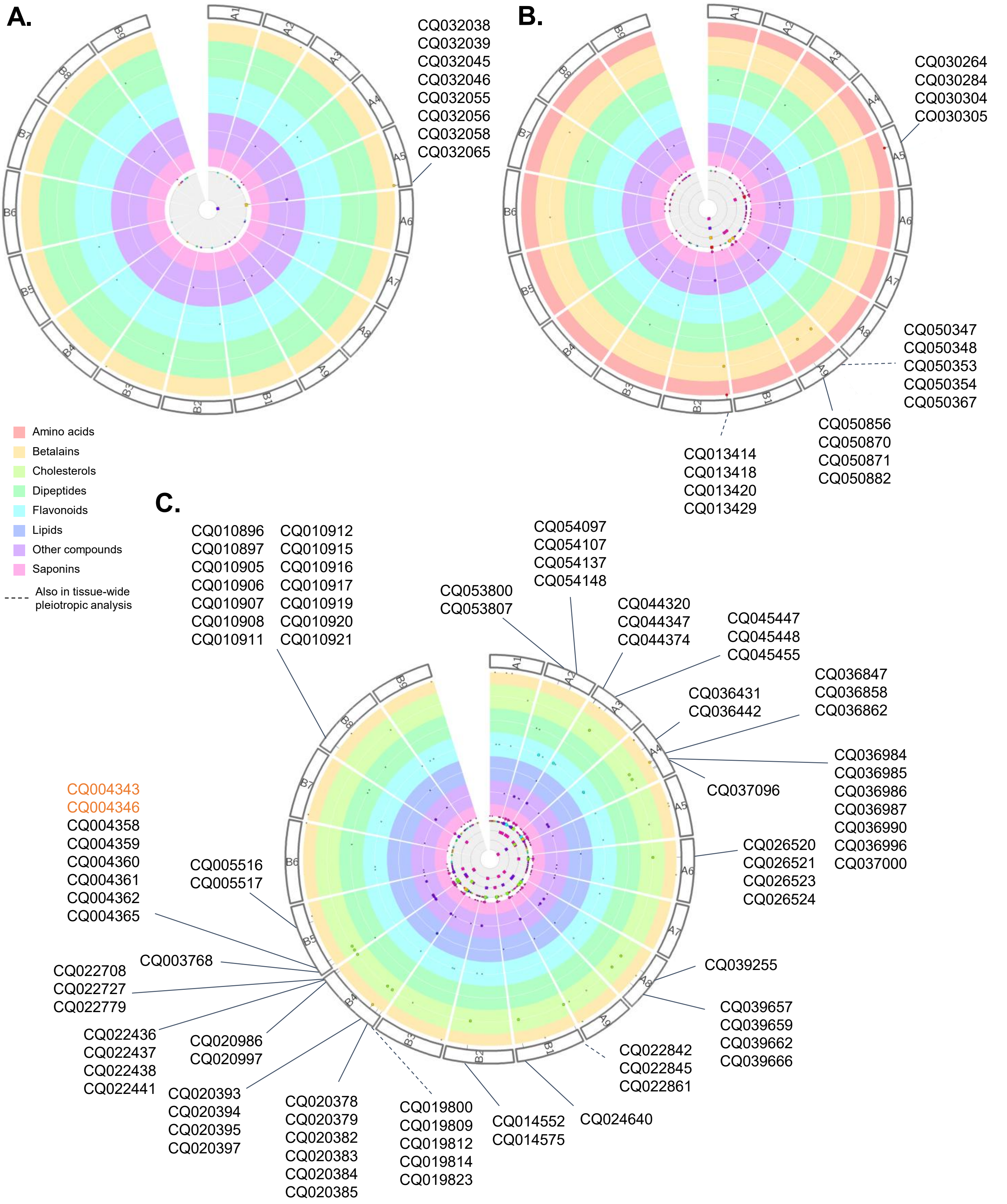

Figure S13. Pleiotropic analysis of annotated secondary metabolites and lipids in leaves, roots and seeds using ≥ four GWAS methods. Pleiotropic analysis of (A) leaves, (B) roots, and (C) seed secondary metabolites in a 50 kb window. The outer ring represents the chromosomes; the inner rings show compound classes per tissue; and the dots indicate SNPs associated with genomic regions. The innermost circle summarizes the total number of associations per QTL. (A = amino acids, B = betalains, D = dipeptides, F = flavonoids, O = others, L = lipids, S = saponins) identifying 1, 4, and 24 common QTL, respectively.

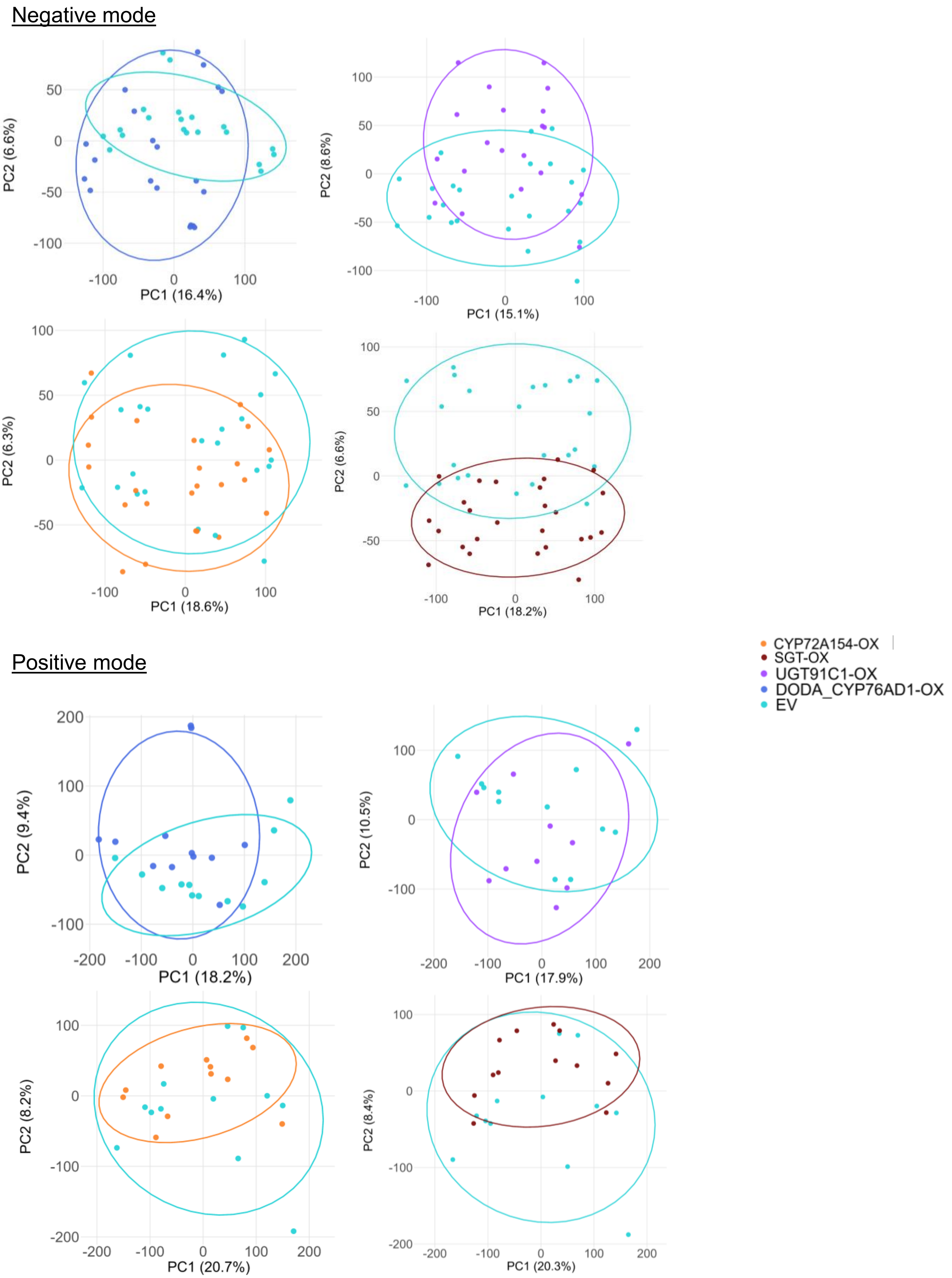

Figure S14. Transient overexpression in *Nicotiana benthamiana* leaves. Transient overexpression (OX) of CYP72A154 ($n_{negative}$ = 34,644 compounds, $n_{positive}$ = 63,358 compounds, $n_{CYP72A154\text{-}OX}$ = 24 lines, n_{EV} = 24 lines), SGT ($n_{negative}$ = 34,990 compounds, $n_{positive}$ = 63,937 compounds, $n_{SGT\text{-}OX}$ = 26 lines, n_{EV} = 24 lines), UGT91C1 ($n_{negative}$ = 35,604 compounds, $n_{positive}$ = 64,617 compounds, $n_{UGT91C1\text{-}OX}$ = 20 lines, n_{EV} = 24 lines), DODA and CYP76AD1 ($n_{negative}$ = 34,799 compounds, $n_{positive}$ = 63,257 compounds, $n_{DODA\text{-}CYP76AD1\text{-}OX}$ = 24 lines, n_{EV} = 22 lines), and empty vector. Metabolic features were detected in negative and positive mode LC-MS.

A.

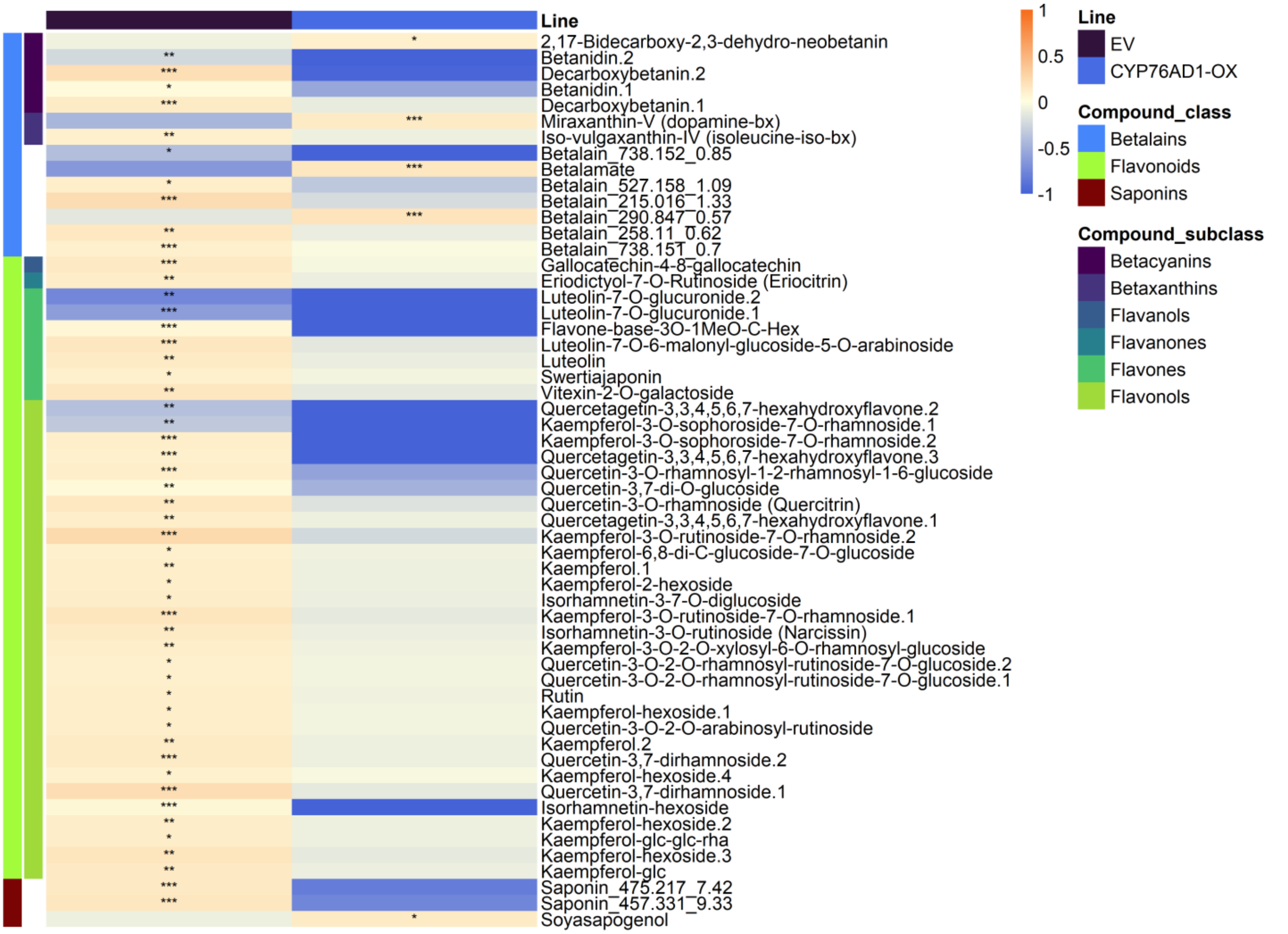

B.

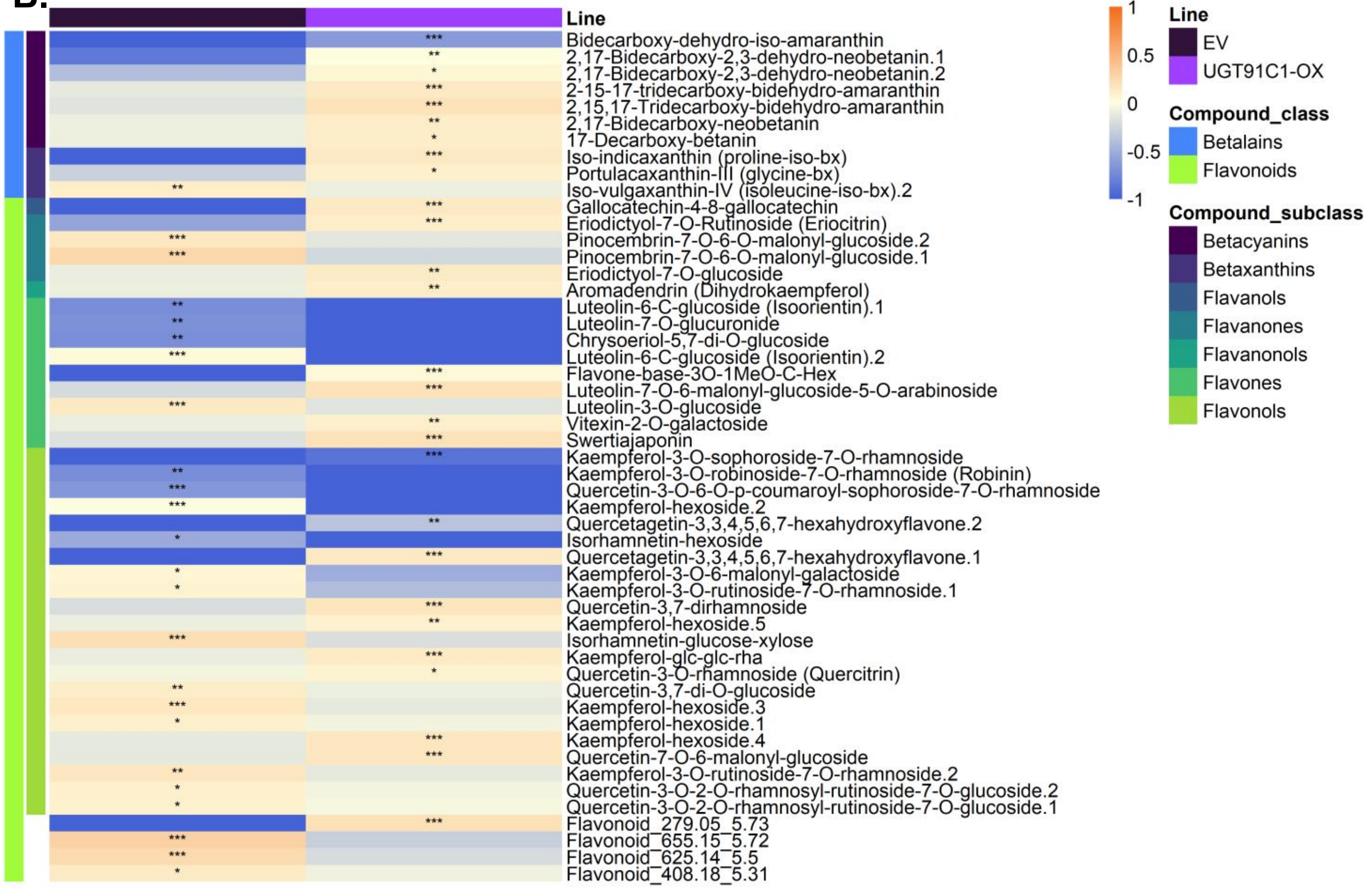

Figure S15. Transient overexpression in *Chenopodium quinoa* leaves. Heatmaps of transient overexpression (OX) of (**A**) CYP76AD1, and (**B**) UGT91C1 compared to empty vector.

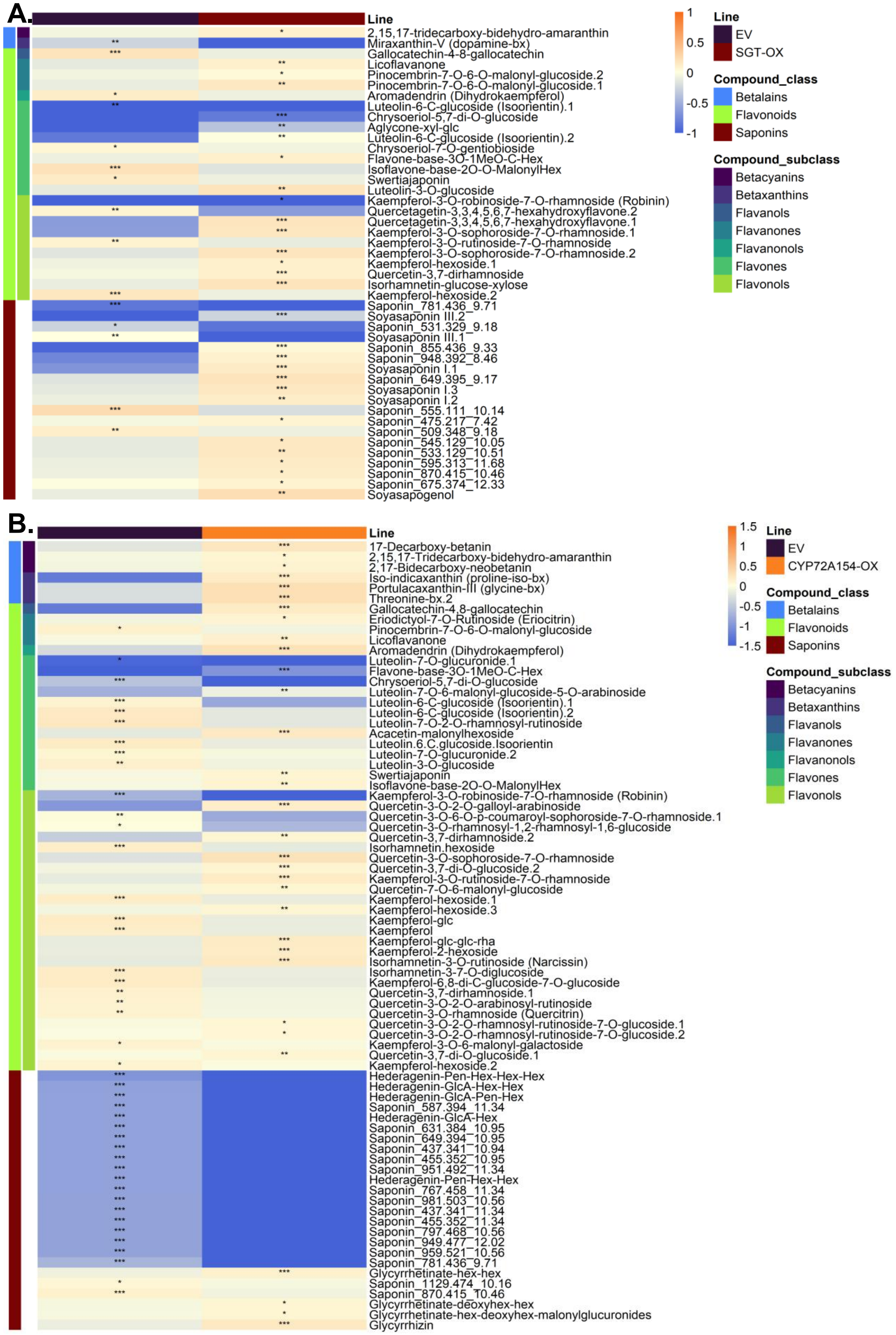

Figure S16. Transient overexpression in *Chenopodium quinoa* leaves. Heatmaps of transient overexpression (OX) of (**A**) soyasapogenol B glucuronide galactosyltransferase (SGT), and (**B**) CYP72A154 compared to empty vector.

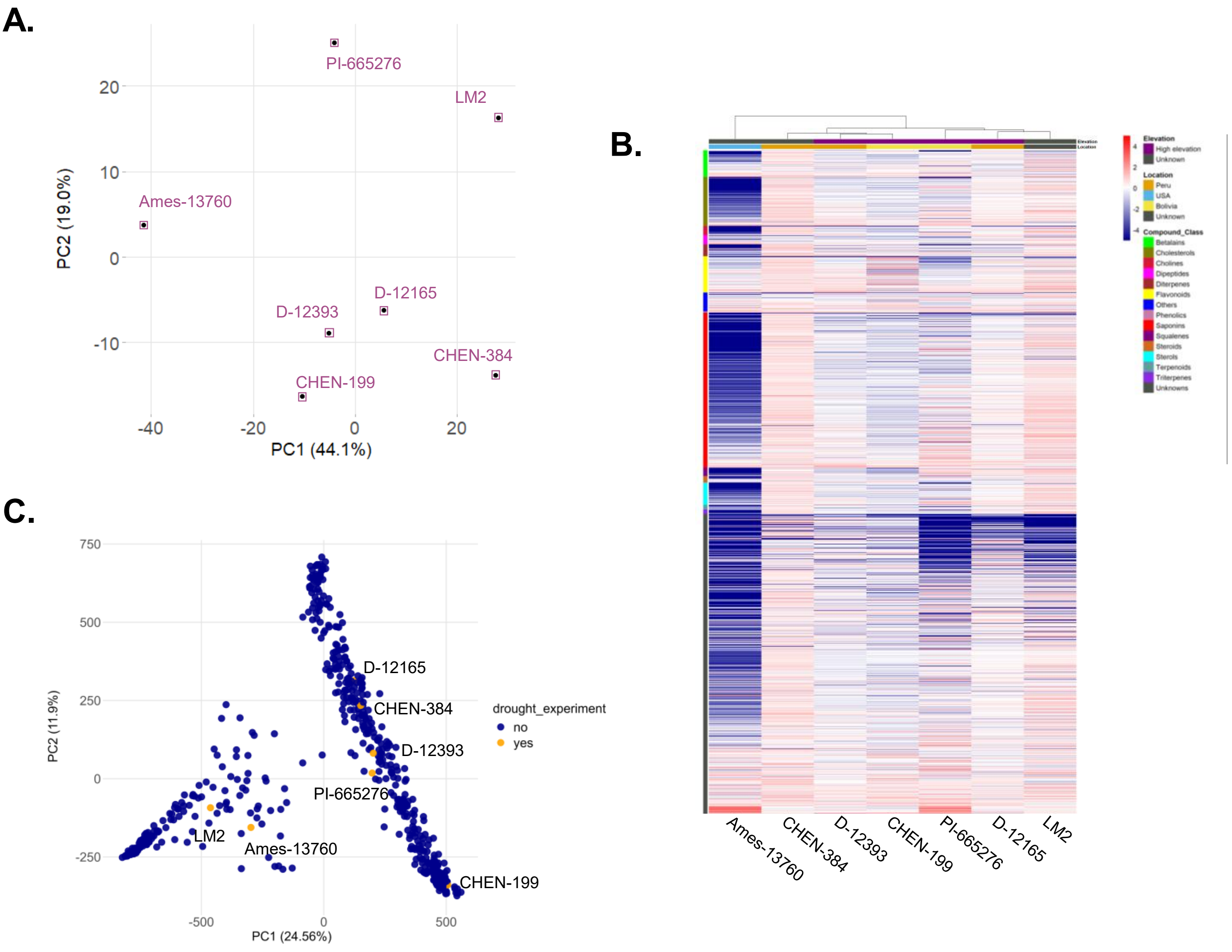

Figure S17. Seed metabolic diversity of 7 selected quinoa accessions for an integrative analysis. (**A**) Principal component analysis (PCA) of 7 accessions using 3029 metabolic features comprising 950 major peaks picked from the chromatograms and 2079 compounds putatively annotated (see material and methods). (**B**) Heatmap calculated using Euclidean distance measure and Ward clustering method. (**C**) PCA of 603 quinoa accessions. The accessions used for the drought experiment are coloured in orange.

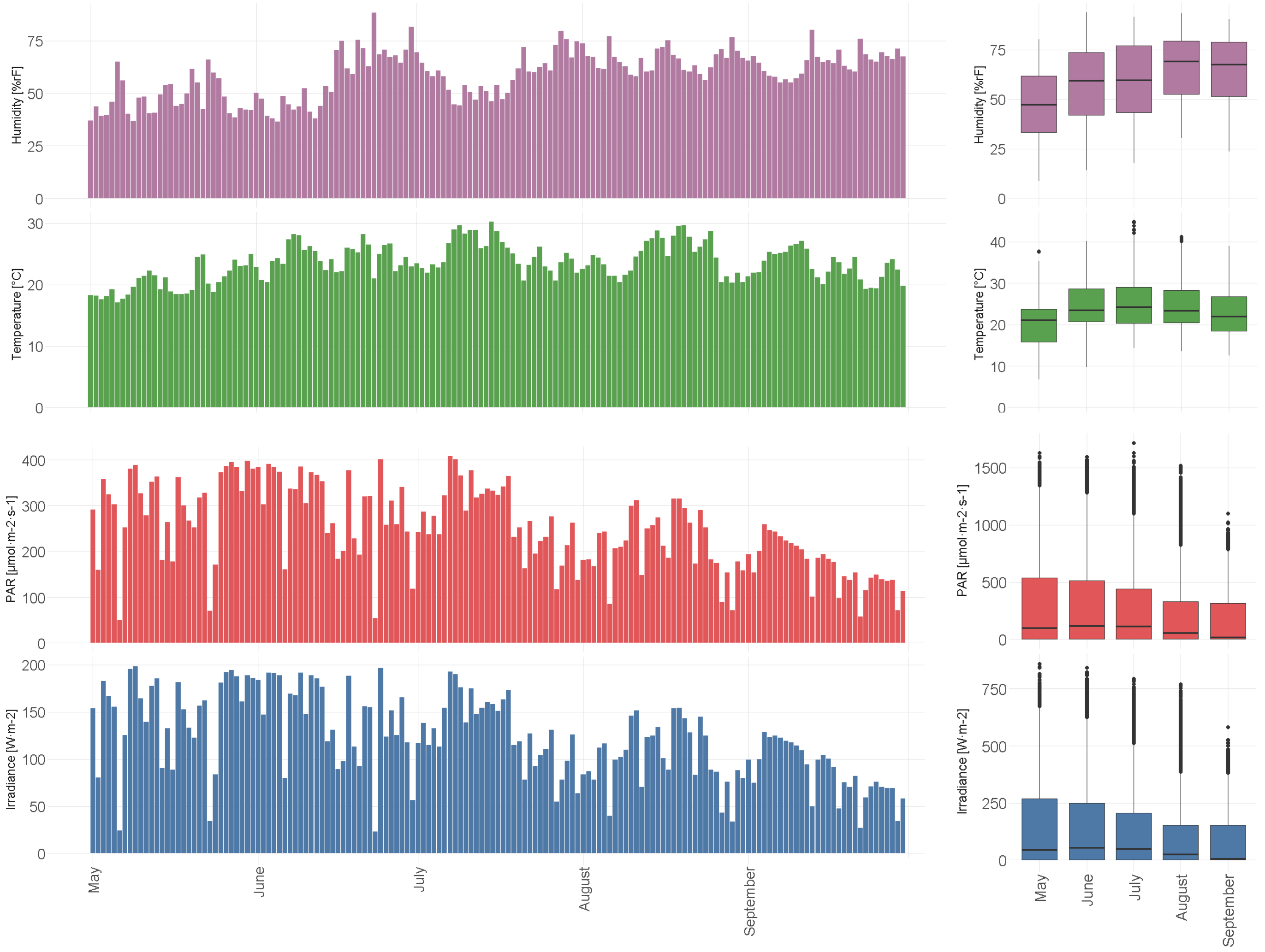

	Minimum	Average	Median	Maximum
Humidity [%rF]	8.4	58.9	60.3	94
Temperature [°C]	6.7	23.5	22.6	44.9
PAR [µmol·m^{-2}·s^{-1}]	0	248.8	81	1717
Irradiance [W·m^{-2}]	0	122.3	37	859

Figure S18. Climate conditions in the polytunnel. Barplot per day and boxplots per month of humidity [%rF], temperature [°C], photosynthetically active radiation (PAR) [µmol·m^{-2}·s^{-1}], and irradiance[W·m^{-2}] from May to September 2023. The table shows the minimum, average, median and maximum values per condition. Boxplots show median, interquartile range (IQR), and 1.5x IQR whiskers.

3 pm (drought plants 31 hours without water)

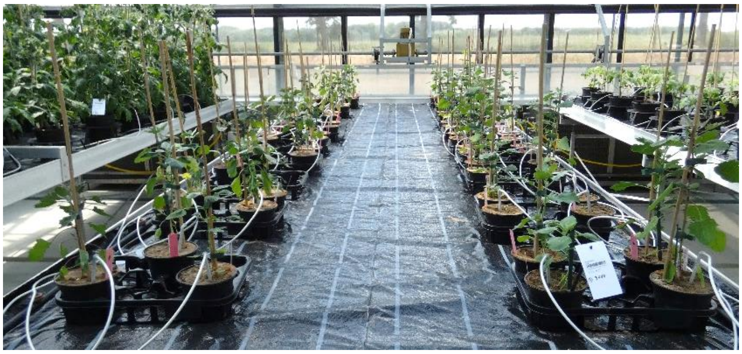

Drought Control

7 am (drought plants 47 hours without water)

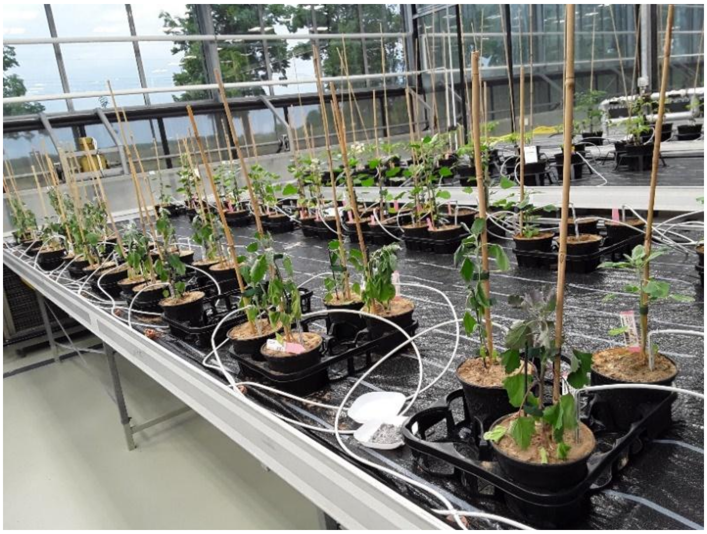

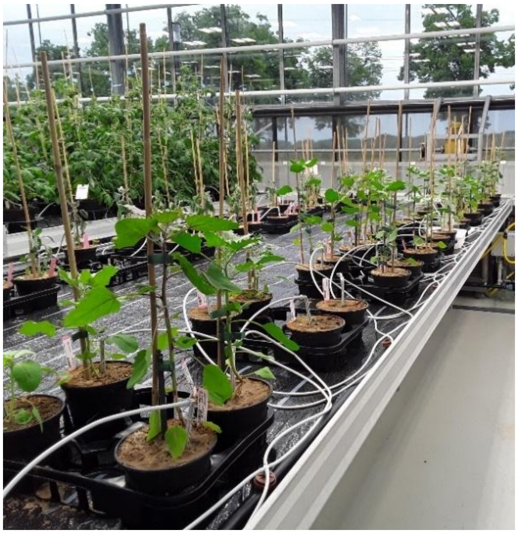

Drought Control

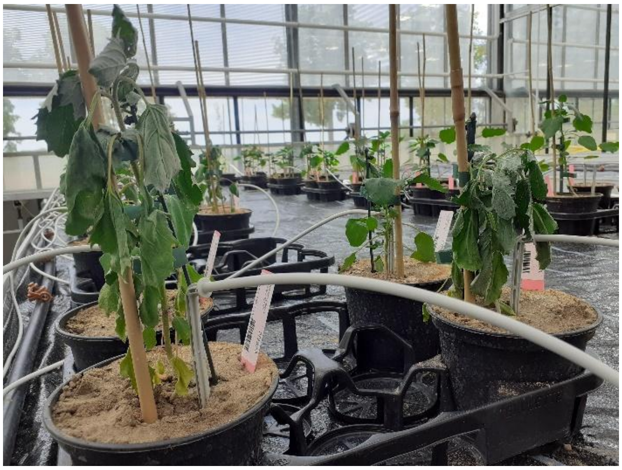

Figure S19. Quinoa drought experiment in the greenhouse. *Chenopodium quinoa* accessions under drought stress were irrigated every second day while control plants every day. The water supply was adjusted according to the turgescence of the control plants. For this experiment, pots with an upper (top opening) diameter of 12.5 cm were used.

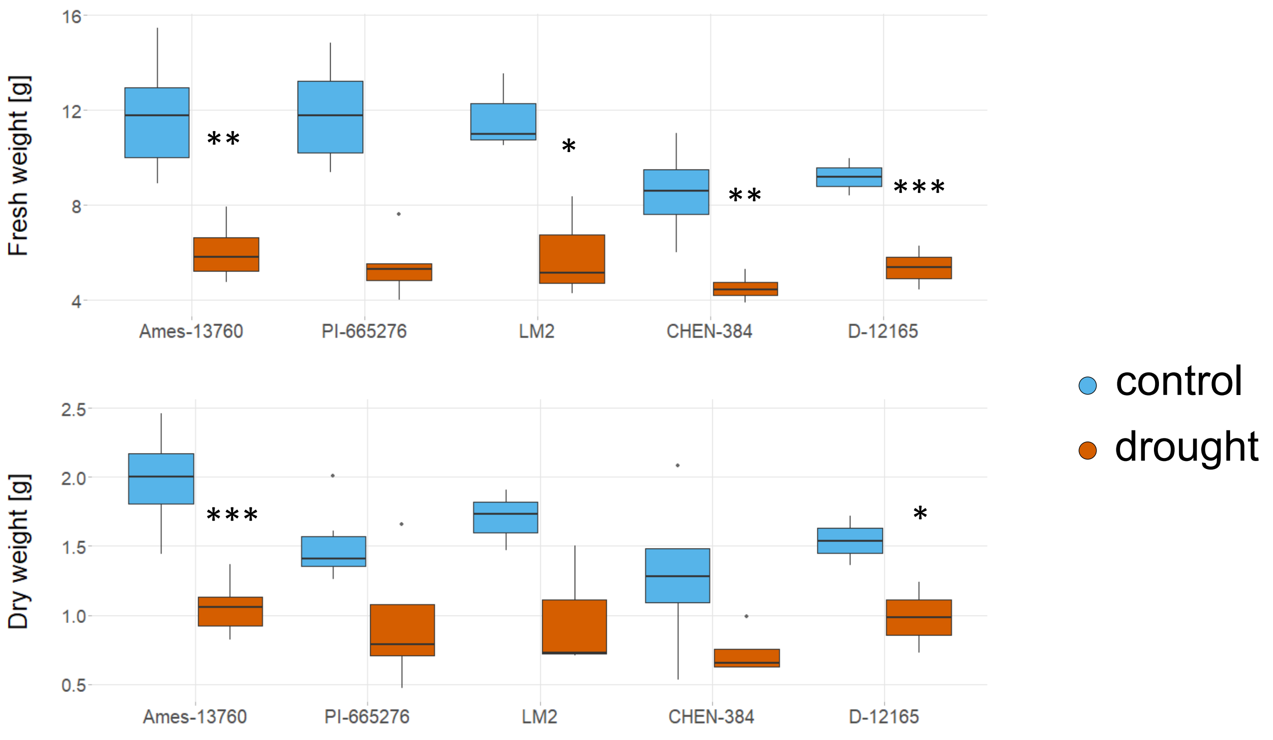

Figure S20. Quinoa drought experiment in the greenhouse. Fresh and dry weight of the *Chenopodium quinoa* accessions under control and drought stress conditions ($n_{Drought} = 5$, $n_{Control} = 5$). Plants under drought were irrigated every second day while control plants every day. The water supply was adjusted according to the turgescence of the control plants. Boxplots show median, interquartile range (IQR), and 1.5x IQR whiskers. Asterisks indicate significances between control and drought (p-value < 0.001***, < 0.01**, < 0.05*) using Student’s t-test.

3 pm (drought plants 31 hours without water)

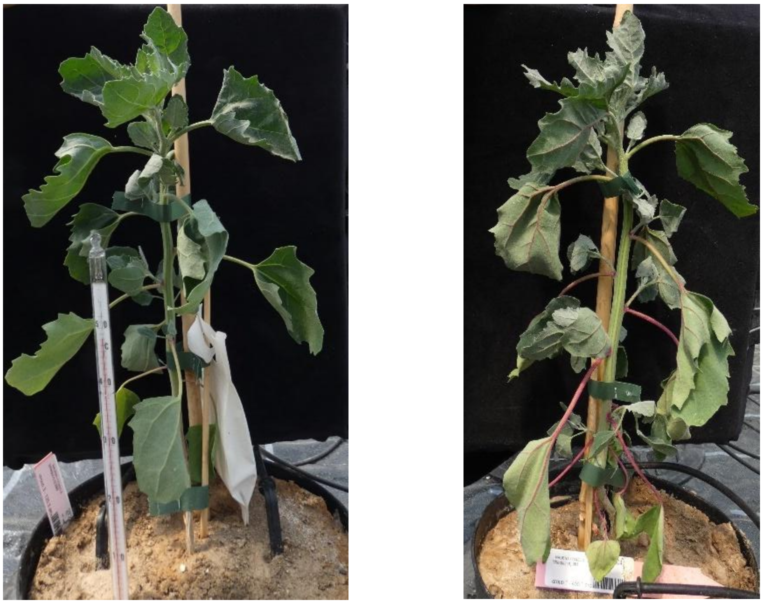

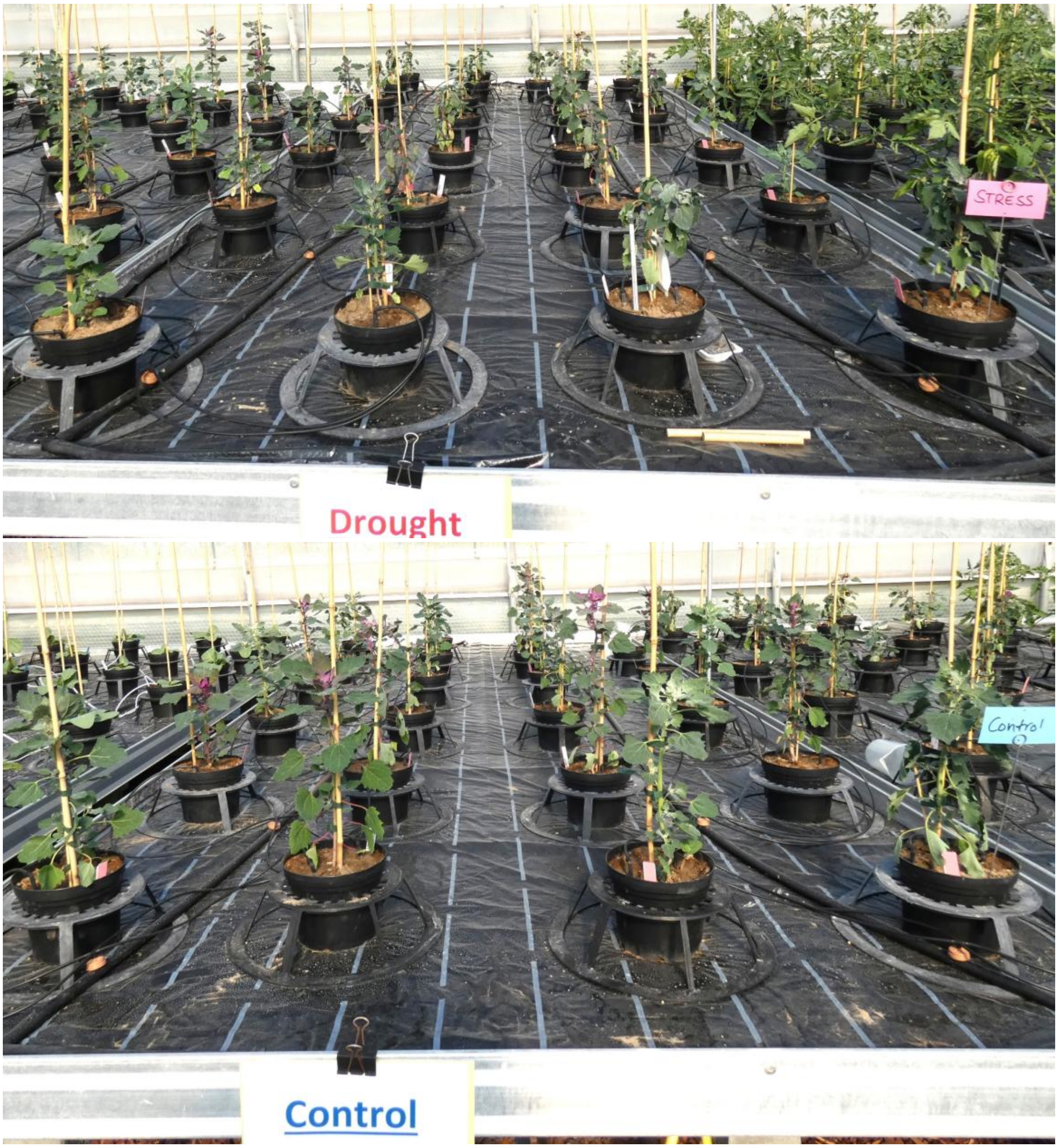

Figure S21. Quinoa drought experiment in the polytunnel. *Chenopodium quinoa* accessions under drought stress were irrigated every second day while control plants every day. The water supply was adjusted according to the turgescence of the control plants. Soil temperature reached 48 °C on 08/06/2023. For this experiment, pots with an upper (top opening) diameter of 19 cm were used.

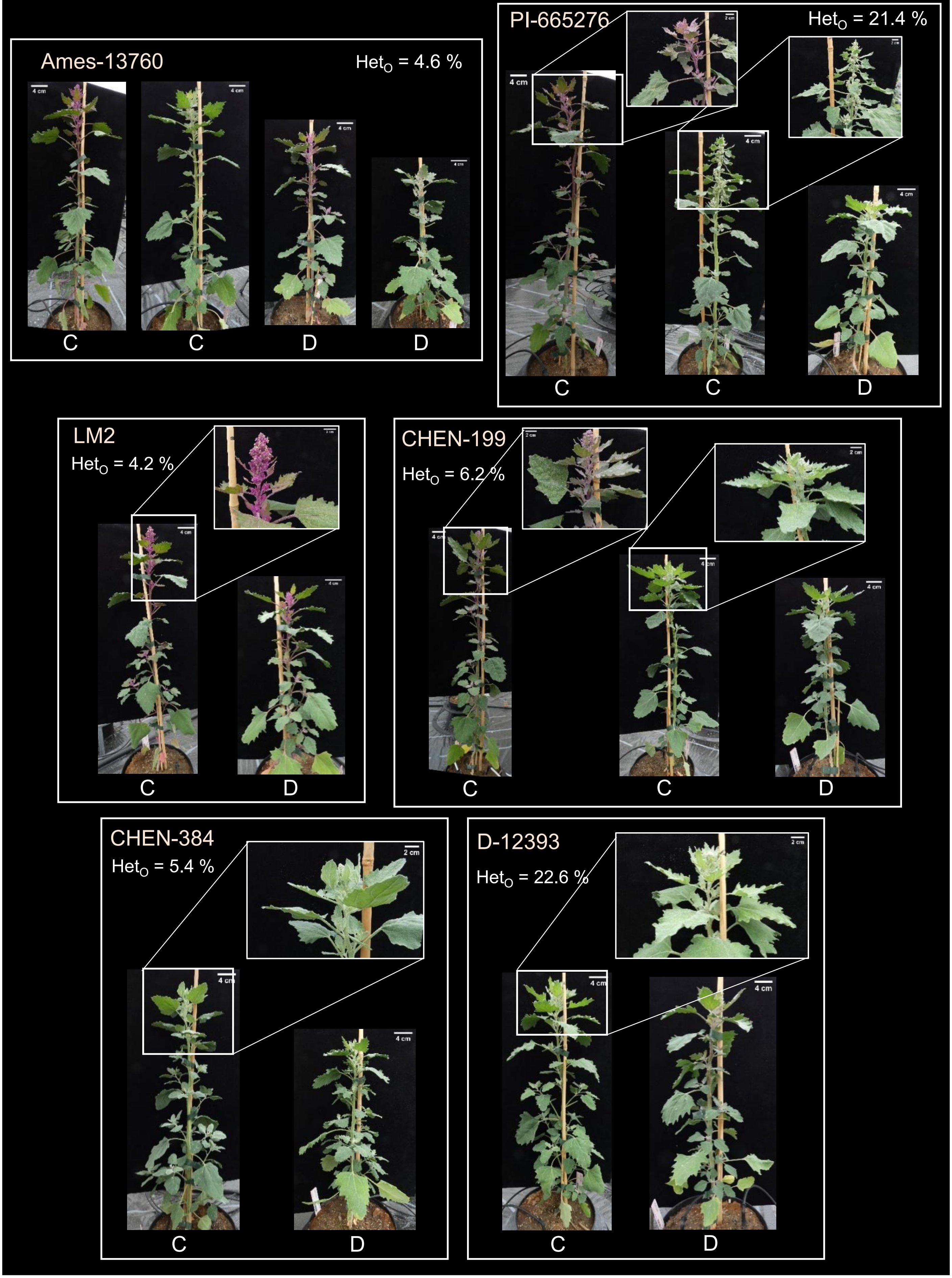

Figure S22. Phenotype of the *Chenopodium quinoa* accessions grown in the polytunnel. Ames-13760, PI-665276, LM2, CHEN-199, CHEN-384, D-12393 under control (C) and drought (D) conditions with calculated observed heterozygosity (Het_O) in percent. Scale bar = 4 cm (whole plant), 2 cm (zoom in). Colors of names indicate clusters in PCA (Figure 8).

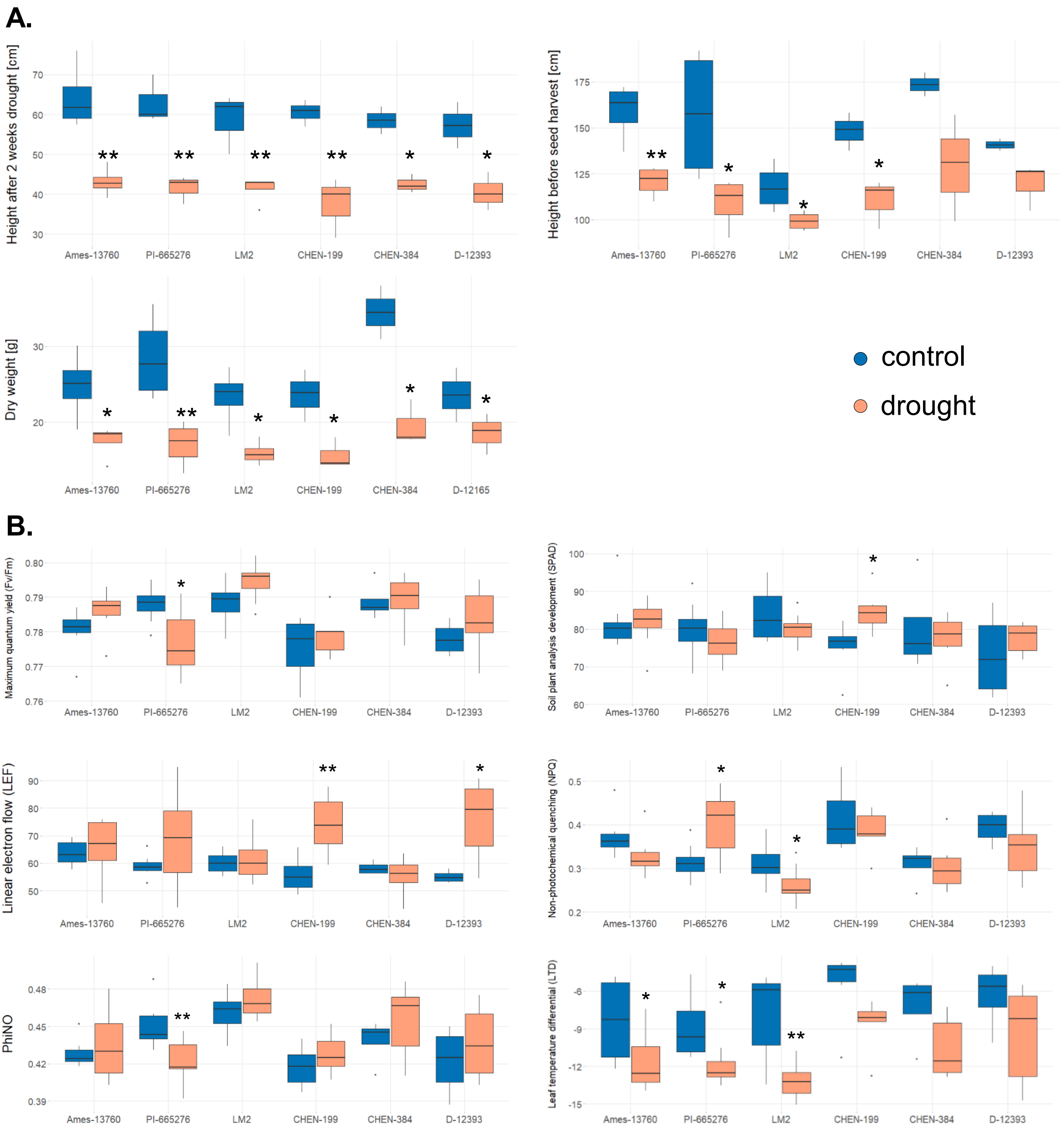

Figure S23. Phenotype of Chenopodium quinoa accessions grown in the polytunnel. (**A**) Height and dry weight of the *C. quinoa* accessions Ames-13760, PI-665276, LM2, CHEN-199, CHEN-384, D-12393 under control (blue) and drought (orange) conditions. (**B**) Photosynthetic activity of the *C. quinoa* accessions ($n_{Drought}$ = 4, $n_{Control}$ = 4). Maximum quantum yield as Fv/Fm, soil plant analysis development (SPAD) value as an indicator of plant nitrogen status and relative chlorophyll, linear electron flow (LEF), nonphotochemical chlorophyll fluorescence quenching (NPQ) as a measure of dissipated heat or fluorescence, PhiNO-ratio as a measure of excited electrons that are lost in non-regulated processes and cause photodamage, leaf temperature differential (LTD), the temperature difference between the leaf and its environment. ***$p < 0.001$, **$p < 0.001$, *$p < 0.01$; Student's *t*-test. Boxplots show median, interquartile range (IQR), and 1.5x IQR whiskers.

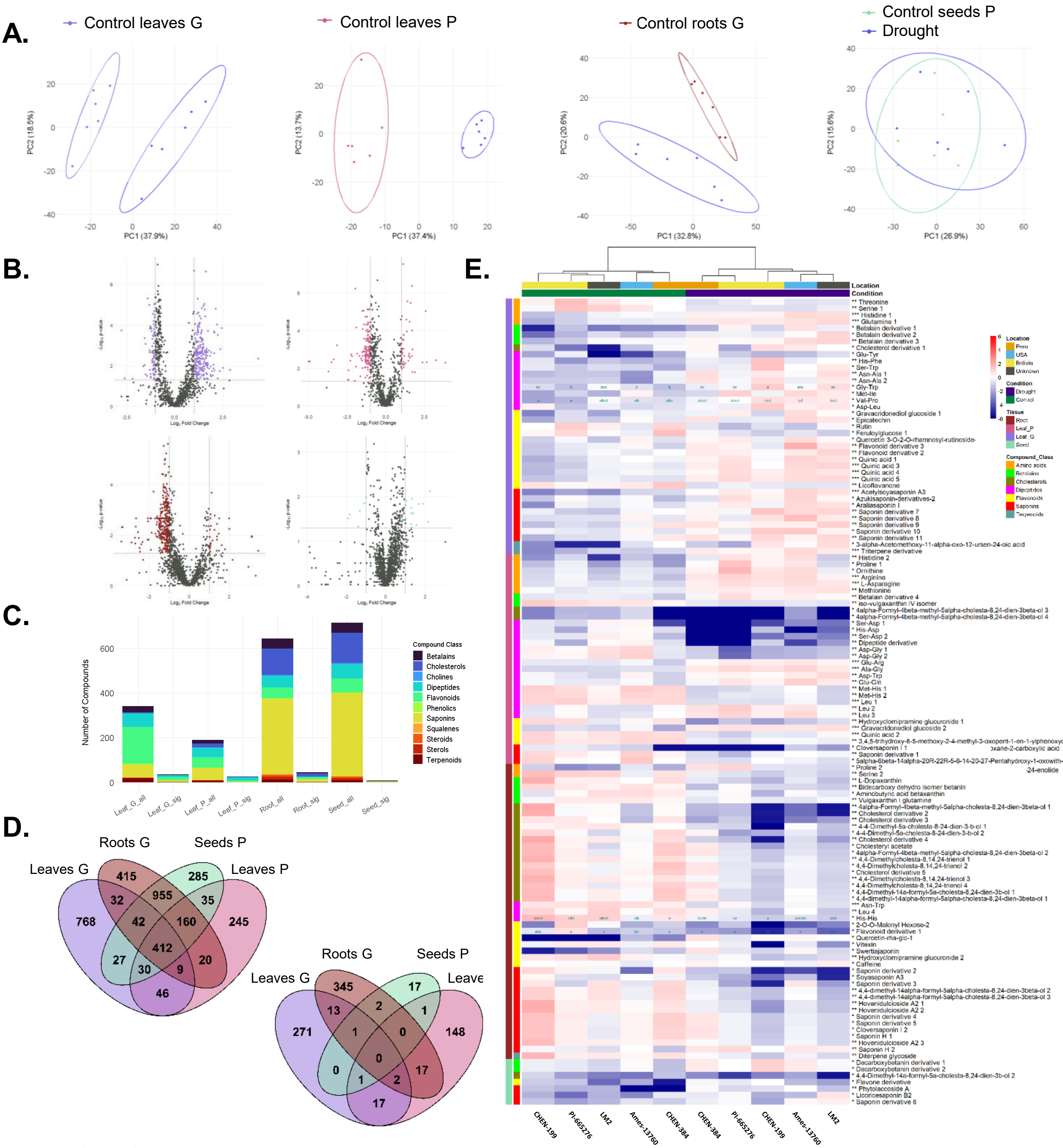

Figure S24. Secondary metabolic diversity of six quinoa accessions under drought conditions. Roots and leaves of the accessions CHEN-199, D-12165, Ames-13760, CHEN-384, PI-665276 and LM2 were harvested in the greenhouse (G) while leaves and seeds of the accessions CHEN-199, Ames-13760, CHEN-384, D-12393, PI-665276 and LM2 were harvested in the polytunnel (P). (**A**) Principal component analysis of 1365, 956, 2045 and 1945 metabolic features of leaves G and P, roots G and seeds P, respectively. Ellipse shows the 80 % confidence interval. (**B**) Volcano plot of leaves grown in the greenhouse (purple) and polytunnel (pink), roots (red) and seeds (green). Significance was calculated by Wilcoxon test. Cut off: $p < 0.05$ and $|\log_2$ fold change$| > 1$. (**C**) Counts of compound classes per tissue without unknown compounds with either all identified compounds ("_all", 549 leaf G, 370 leaf P, 858 root and 940 seed compounds) or only compounds with $p < 0.05$ ("_sig", 117 leaf G, 138 leaf P, 105 root and 61 seed compounds). (**D**) Venn-diagram of all and only significant (p-value < 0.05, log2 fold change > |1|) common compounds in leaves G and P, roots (G) and seeds (P). (**E**) Heatmap of significant (p-value < 0.05, $\log_2$ fold change > |1|; total: 112 compounds; Leaves G: 35 compounds – 3 betalains, 1 cholesterols, 9 dipeptides, 12 flavonoids, 8 saponins, 2 terpenoids; Leaves P: 26 compounds – 2 betalains, 2 cholesterols, 15 dipeptides, 4 flavonoids, 3 saponins; Roots: 44 compounds – 4 betalains, 15 cholesterols, 3 dipeptides, 8 flavonoids, 13 saponins, 1 terpenoid; Seeds: 7 compounds – 2 betalains, 1 cholesterols, 1 flavonoids, 3 saponins) compounds of the five common accessions grown in the greenhouse and the polytunnel identified using Wilcoxon test.

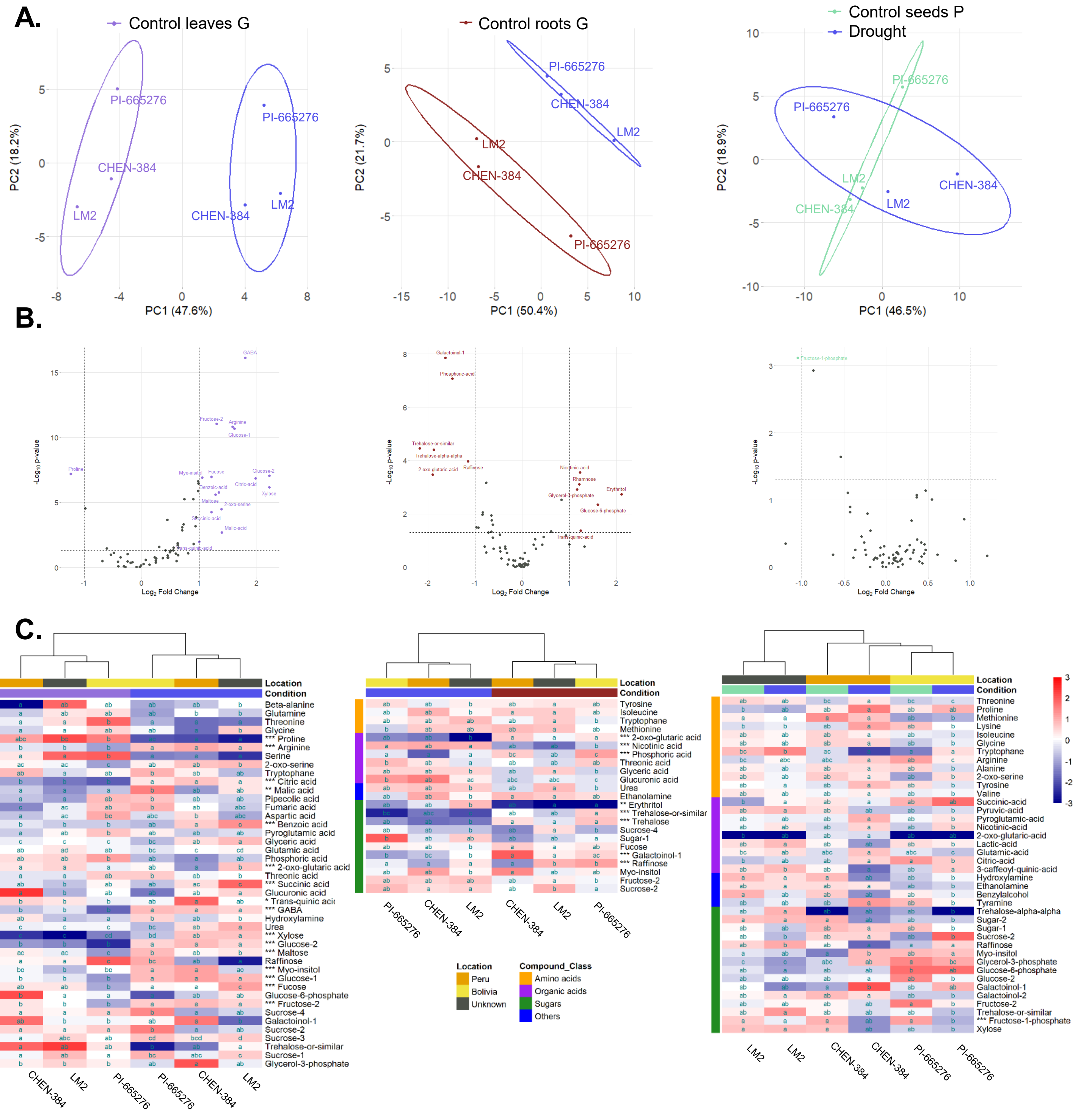

Figure S25. Primary metabolites of three quinoa accessions under drought conditions. Leaves (purple), roots (red) from the greenhouse and seeds (green) from the polytunnel of the accessions CHEN-384, PI-665276 and LM2 were utilized for GC-MS analysis. (**A**) Principal component analysis with the 80 % confidence interval and (**B**) volcano plot of control versus drought of all accessions calculated by Wilcoxon test with cut off criterion of $p < 0.05$ and $|\log_2$ fold change$| > 1$ of 69 (18 amino acids, 20 organic acids, 24 sugars, 7 others), 67 (17 amino acids, 20 organic acids, 24 sugars, 7 others) and 70 (18 amino acids, 20 organic acids, 25 sugars, 7 others) primary metabolic features of leaves, roots and seeds, respectively. (**C**) Heatmap of significant compounds of the five common accessions grown in the greenhouse and the polytunnel (43 compounds of leaves, 23 compounds of roots, 40 compounds of seeds). Letters indicate significances calculated by Kruskal-Wallis test and post hoc Dunn's test with *p*-value < 0.05. Asterisks next to the name indicate significances from (B; p-value < 0.001***, < 0.01**, < 0.05*).

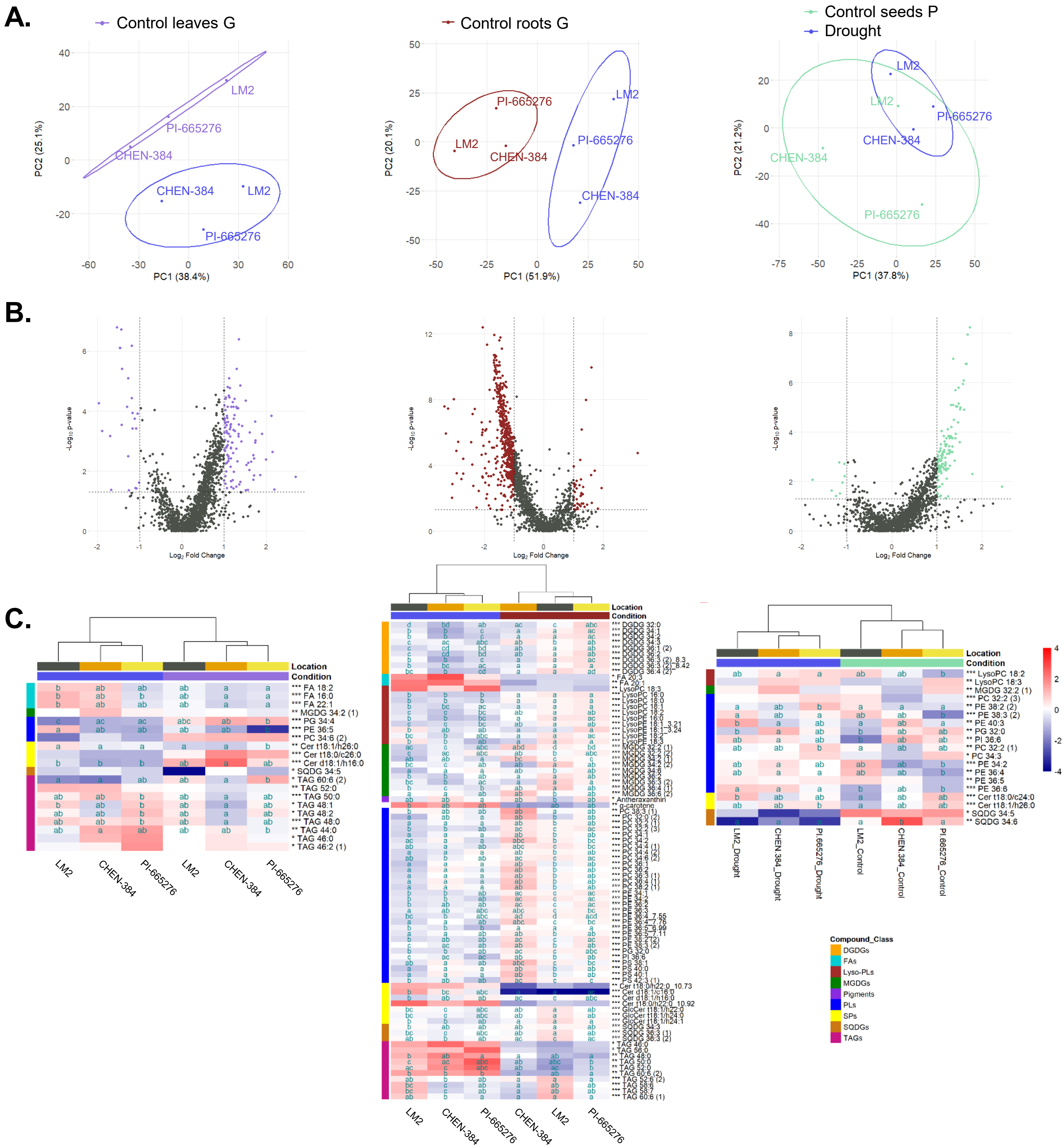

Figure S26. Lipids of three quinoa accessions under drought conditions. Leaves (purple), roots (red) from the greenhouse and seeds (green) from the polytunnel of the accessions CHEN-384, PI-665276 and LM2 were utilized for UPLC-MS analysis. (**A**) Principal component analysis with the 80 % confidence interval and (**B**) volcano plot of control versus drought of all accessions calculated by Wilcoxon test with cut off criterion of $p < 0.05$ and $|\log_2 \text{fold change}| > 1$ of 1707 (7 pigments, 20 fatty acids, 224 annotated lipids, 627 less confident annotated lipids and 829 unknown), 1736 (7 pigments, 20 fatty acids, 224 annotated lipids, 637 less confident annotated lipids and 843 unknown) and 1605 (7 pigments, 20 fatty acids, 226 annotated lipids, 629 less confident annotated lipids and 768 unknown) lipid features of leaves, roots and seeds, respectively. (**C**) Heatmap of significant compounds (20 compounds of leaves: 4 fatty acids (FAs), 1 monogalactosyldiacylglycerol (MGDG), 3 phospholipids (PLs), 3 sphingolipids (SPs), 1 sulfoquinovosyldiacylglycerol (SQDG), 9 triacylglycerols (TAGs); 82 compounds of roots: 9 digalactosyldiacylglycerols (DGDGs), 2 FAs, 10 *lyso*-PLs, 9 MGDGs, 2 pigments, 30 PLs, 7 SPs, 3 SQDGs, 10 TAGs; 19 compounds of seeds: 2 *lyso*-PLs, 1 MGDG, 12 PLs, 2 SPs, 2 SQDGs). Letters indicate significances calculated by Kruskal-Wallis test and post hoc Dunn's test with *p*-value < 0.05. Asterisks next to the name indicate significances from (B; *p*-value < 0.001***, < 0.01**, < 0.05*).

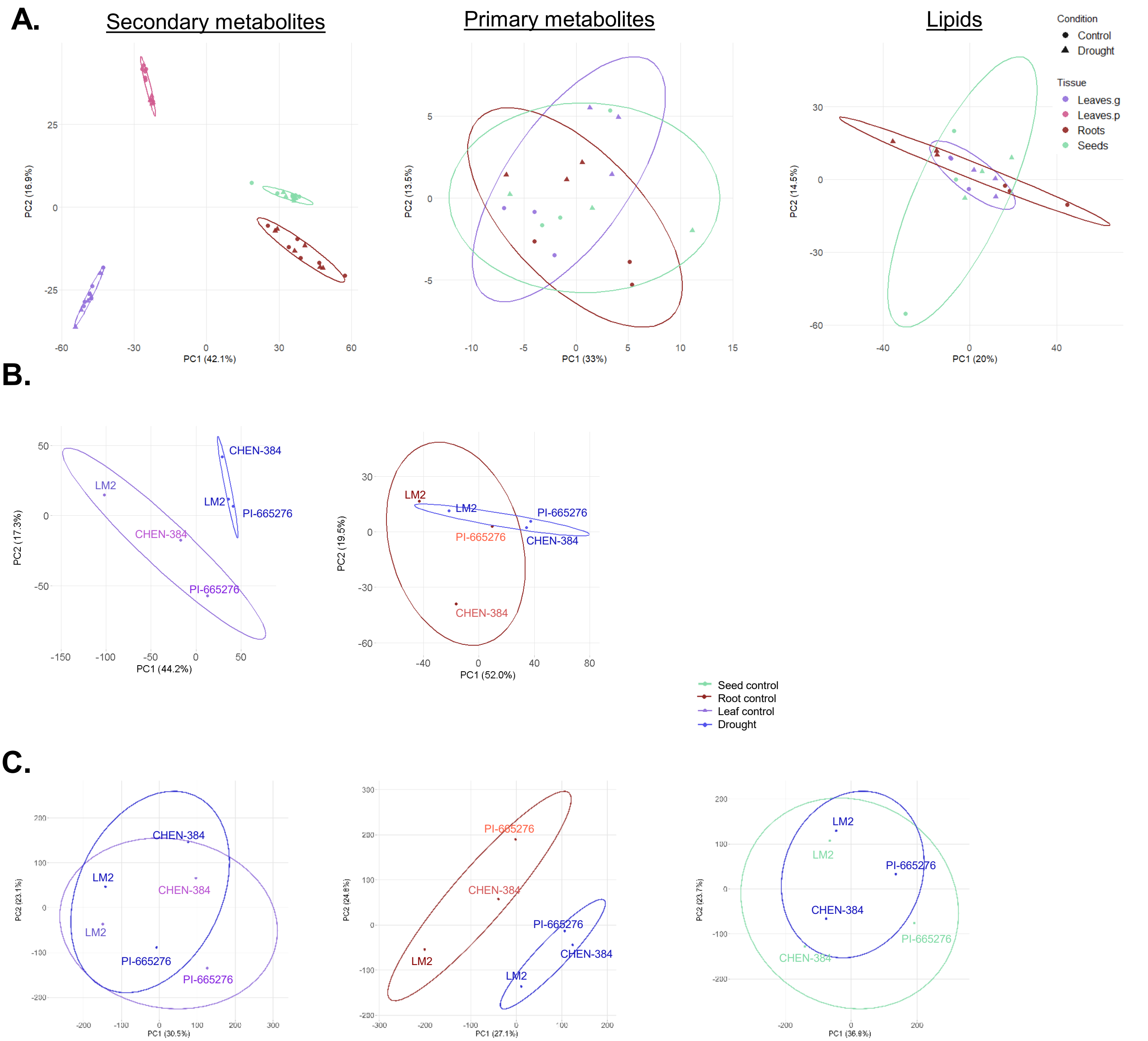

Figure S27. Metabolic, proteomic and transcriptomic diversity of quinoa accessions under drought conditions. (**A**) Metabolic diversity of control versus drought CHEN-384, LM2, PI-665276, CHEN-199, Ames-13760 and either D-12393 or D-12165 in leaves, seeds and roots. Principal component analysis of secondary (n = 3,480 metabolites), primary (n = 72) and lipid (n = 1,808 metabolites) features from leaves of the greenhouse (g) or polytunnel (p), roots and seeds. (**B**) Principal component analysis of 6,574 proteins of quinoa leaves (purple) and 2,001 proteins of quinoa roots (red) of the accessions CHEN-384, PI-665276 and LM2. (**C**) Principal component analysis of 47,732 genes of quinoa leaves (purple), 50,332 genes of quinoa root (red) and 46,557 genes of quinoa seeds (green). Ellipse shows the 80 % confidence interval.

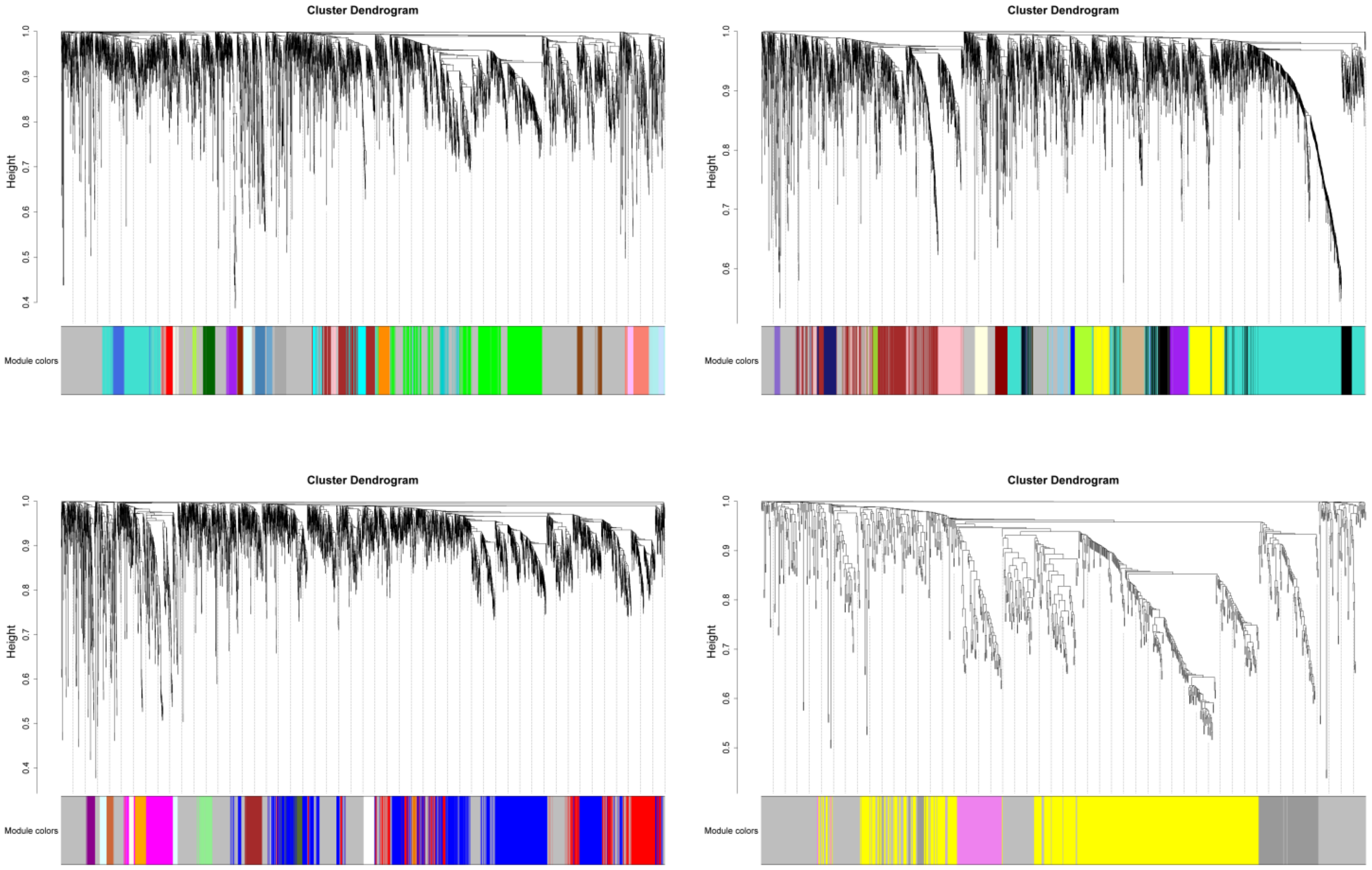

Figure S28. Feature dendrograms of weighted gene co-expression network analysis (WGCNA) identifying 46 modules of four block wise clusters. First cluster comprised 4998 features, the second 4975, the third 4863 and the fourth 972.

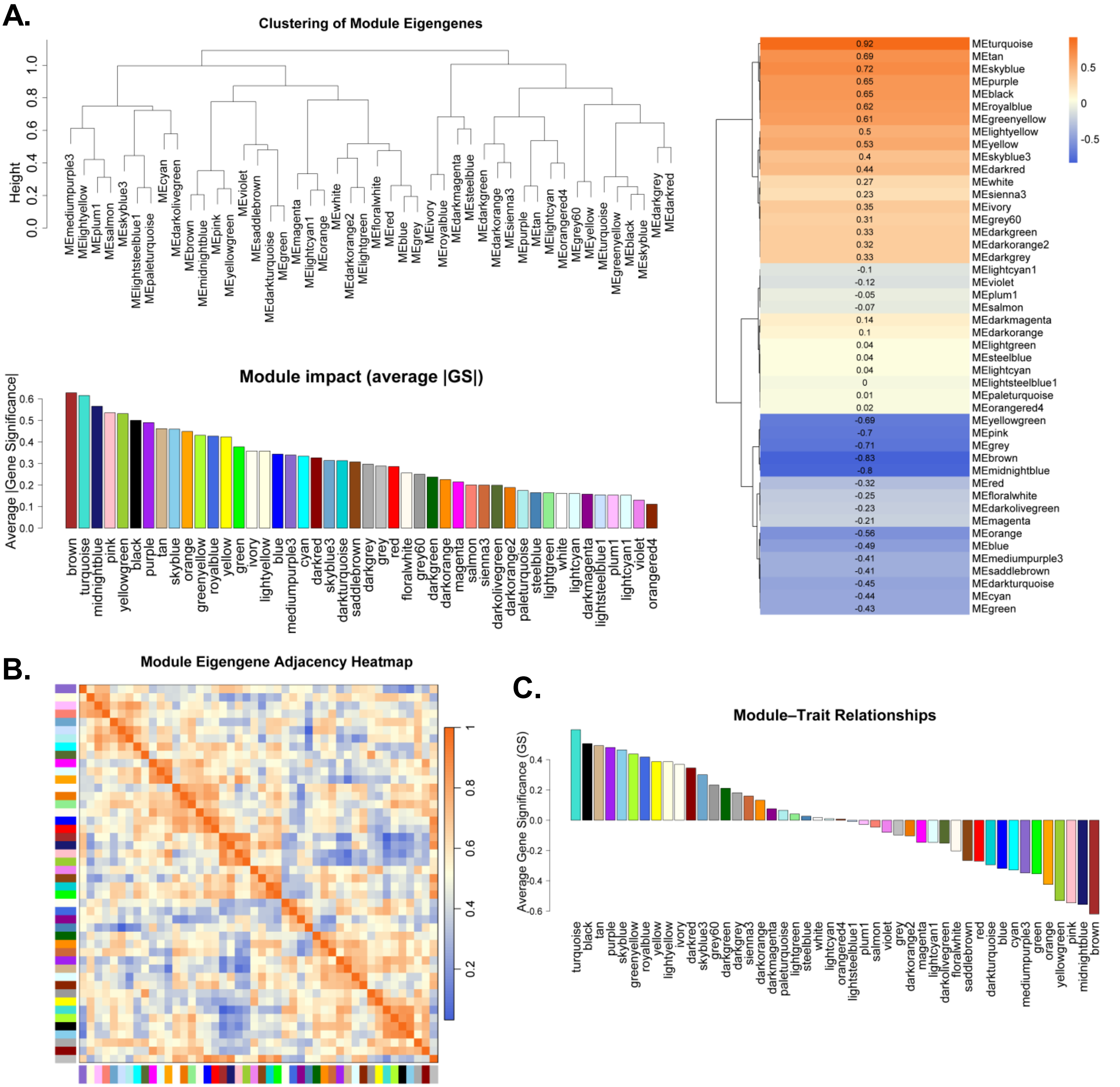

Figure S29. Module overview of weighted gene co-expression network analysis (WGCNA). WGCNA identified 46 modules across 15,809 proteomic, metabolomic and transcriptomic features. (**A**) Dendrogram of the clustering of module eigengenes with module impact as average |gene significance| (GS) as bar plot and as heatmap representation. (**B**) Heatmap of module eigengene adjacency. (**C**) Module-trait relationship of weighted gene co-expression network analysis (WGCNA) of 15,809 metabolic, transcriptomic and proteomic features across leaf, root and seed tissues of CHEN-384, LM2, and PI-665276.

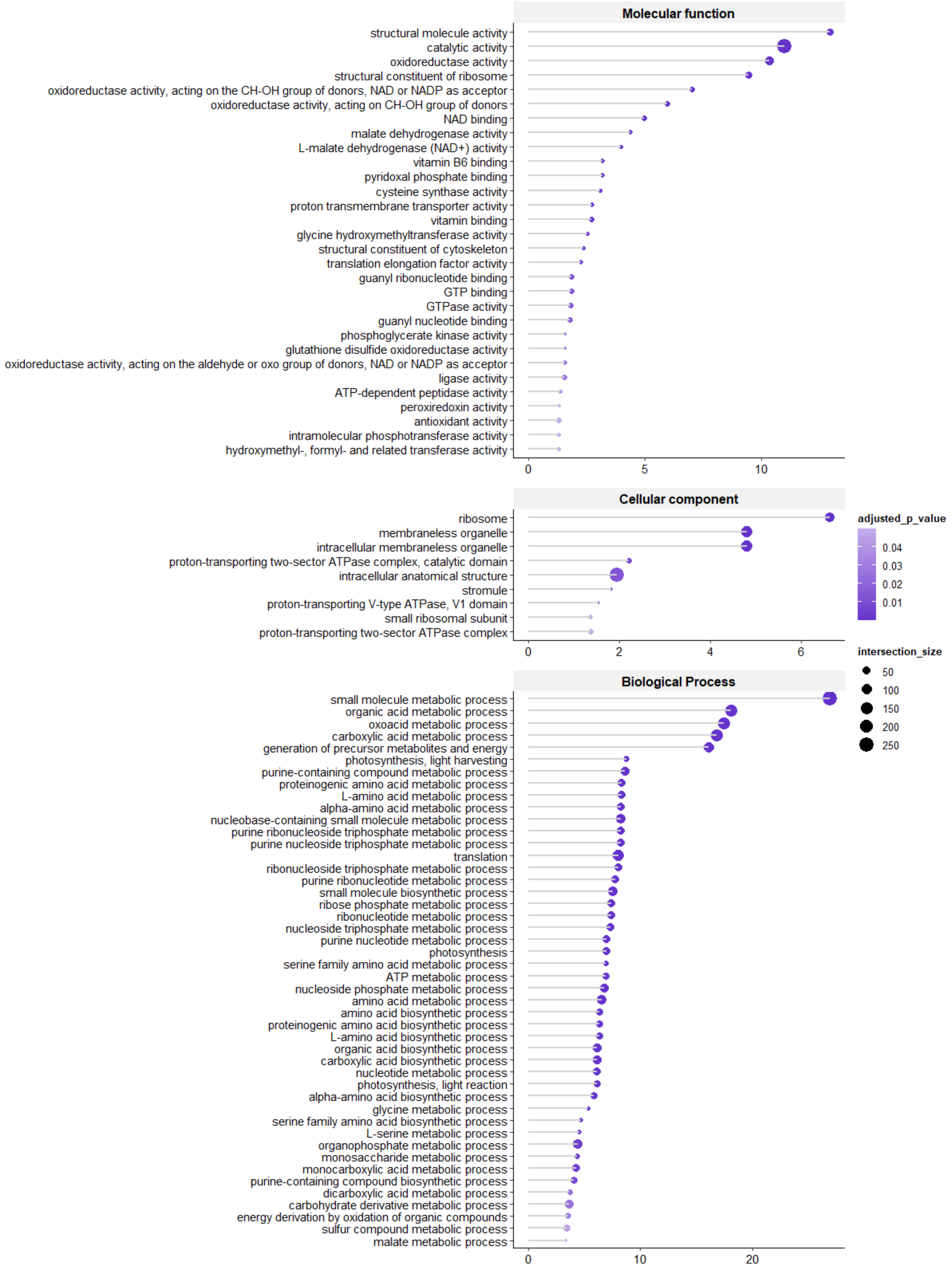

Figure S30. Gene ontology assignment of the leaf proteome of PI-665276, LM2, and CHEN-384 grown in the greenhouse under control and drought conditions. Significant proteins of leaves grown under drought and control conditions calculated by either Student's *t*-test or by Wilcoxon test based on their normal distribution. Cut-off criterion: p-value < 0.05, $|\log_2 \text{fold change}| > 1$. For GO analysis g:Profiler was used with a cut-off criterion of adjusted p-value < 0.05 for molecular function and cellular component and < 0.001 for biological process.

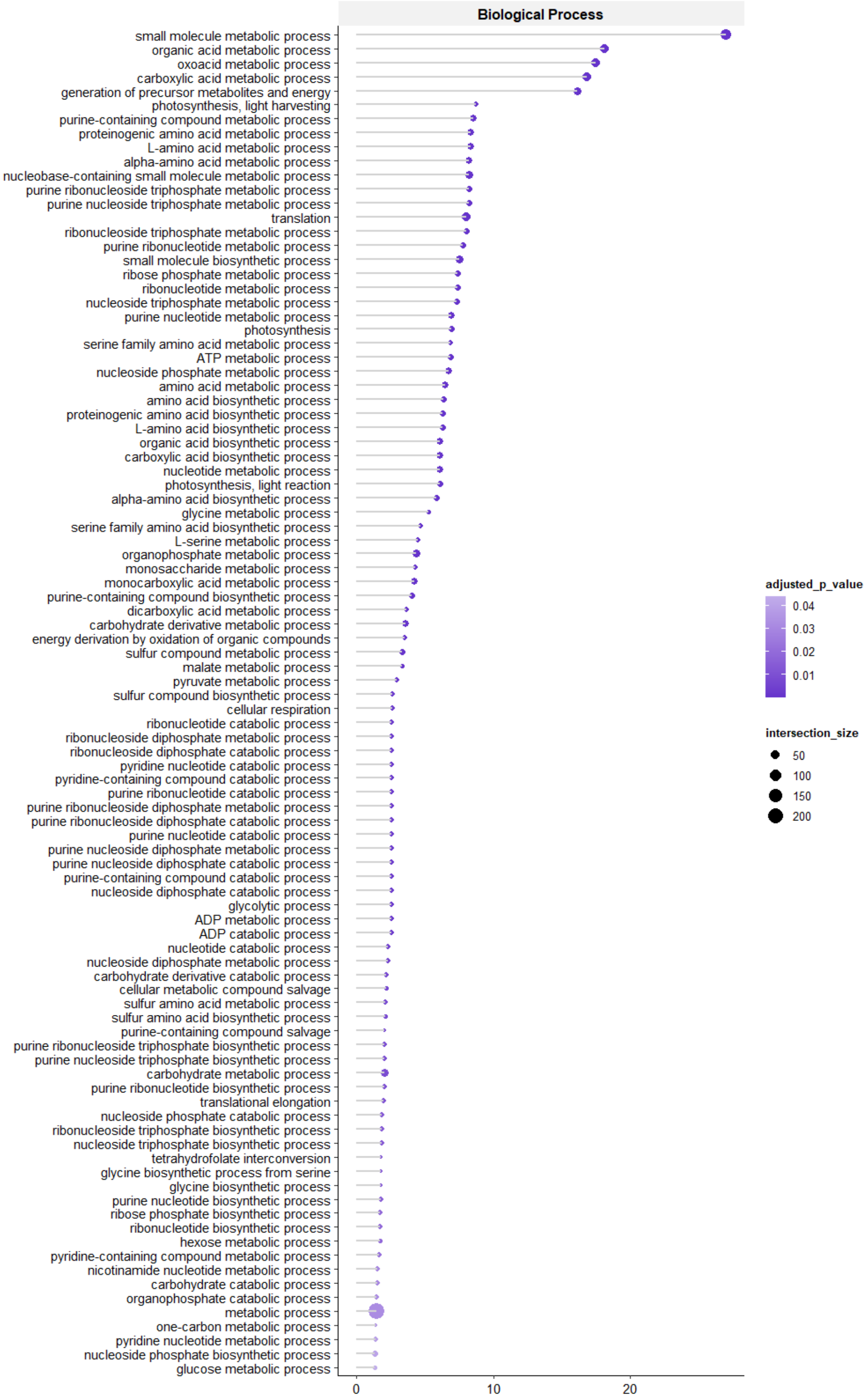

Figure S31. Significant leaf proteins of PI-665276, LM2, and CHEN-384 with an assignment of a biological process during gene ontology analysis. Significant proteins of leaves grown under drought and control conditions calculated by either Student's *t*-test or by Wilcoxon test based on their normal distribution. Cut-off criterion: *p*-value < 0.05, $|\log_2$ fold change$| > 1$. For GO analysis g:Profiler was used with a cut-off criterion of adjusted *p*-value < 0.05.

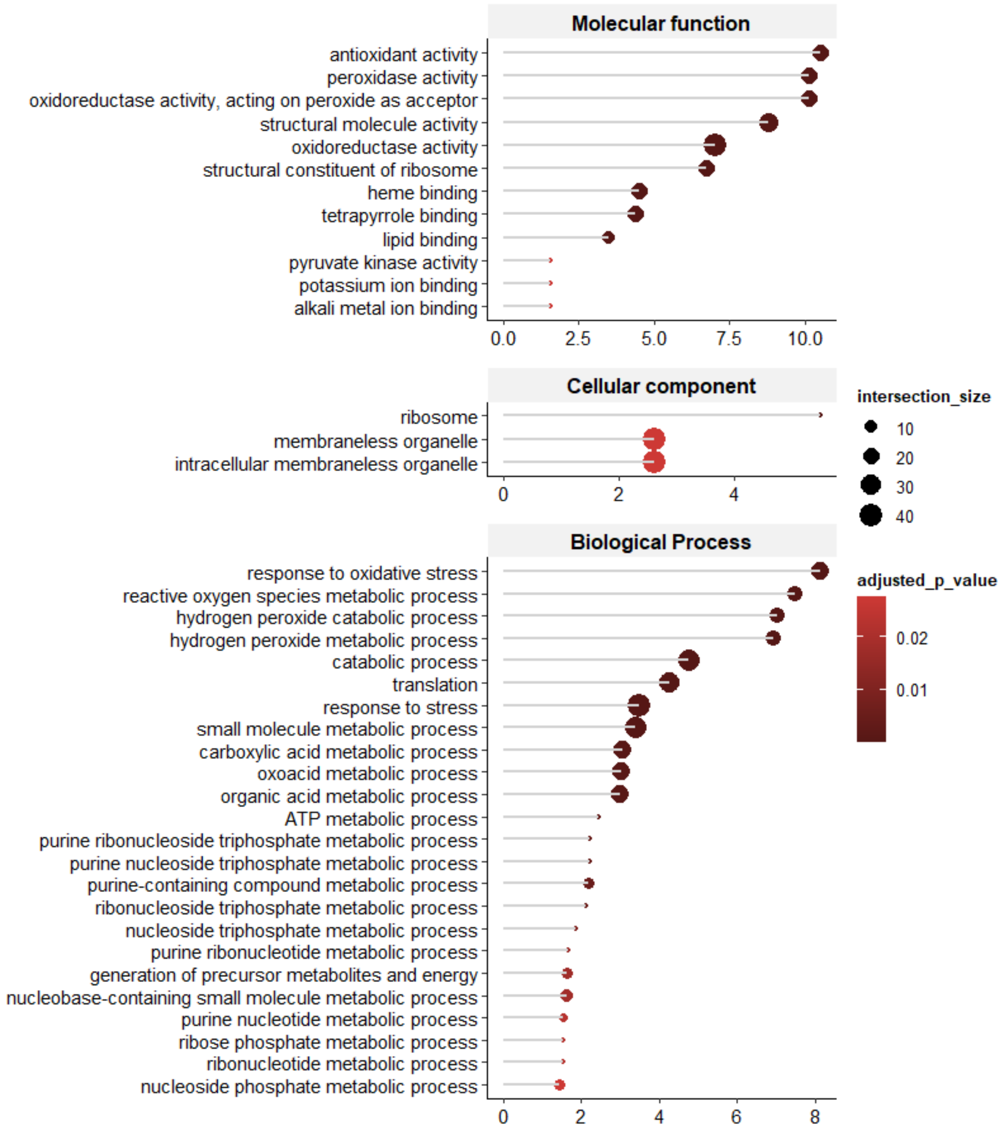

Figure S32. Gene ontology assignment of the root proteome of PI-665276, LM2, and CHEN-384 grown in the greenhouse under control and drought conditions. Significant proteins of leaves grown under drought and control conditions calculated by either Student's *t*-test or by Wilcoxon test based on their normal distribution. Cut-off criterion: *p*-value < 0.05, |$\log_2$ fold change| > 1. For GO analysis g:Profiler was used with a cut-off criterion of adjusted *p*-value < 0.05.

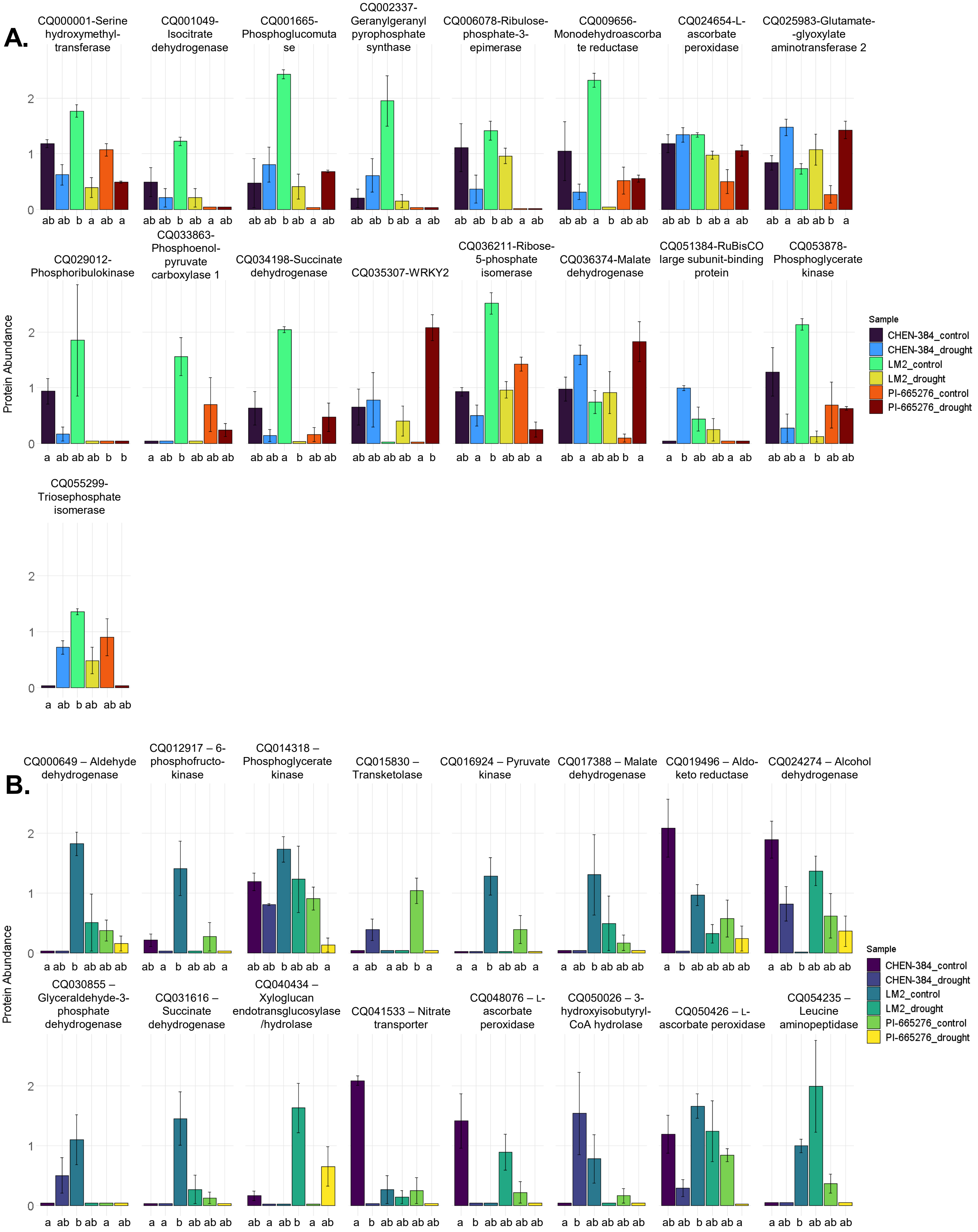

Figure S33. Selected differential expressed proteins of leaves and roots of PI-665276, LM2, and CHEN-384 grown in the greenhouse under control and drought conditions. Proteins involved in drought response in (**A**) leaves ($n_{drought/control}$ = 3 replicates) and (**B**) roots ($n_{drought/control}$ = 3 replicates). Significances were determined using Kruskal-Wallis test with post hoc Dunn's test. Data are presented as mean ± standard deviation, letters indicate significance $p < 0.05$.

A.

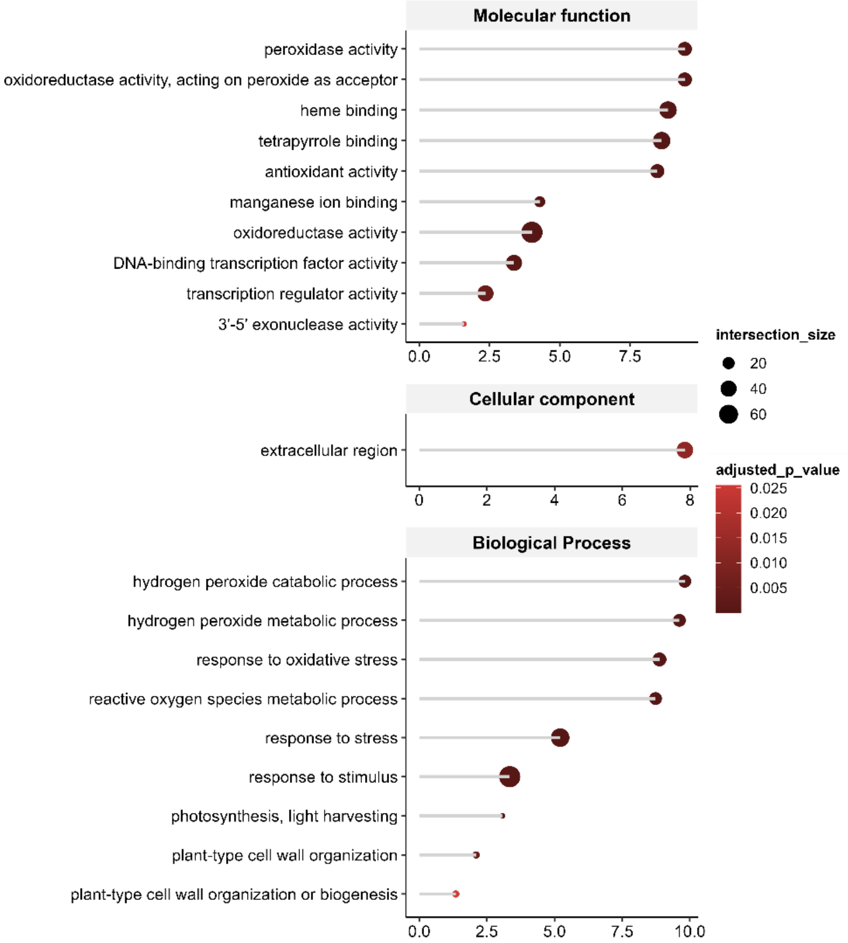

B.

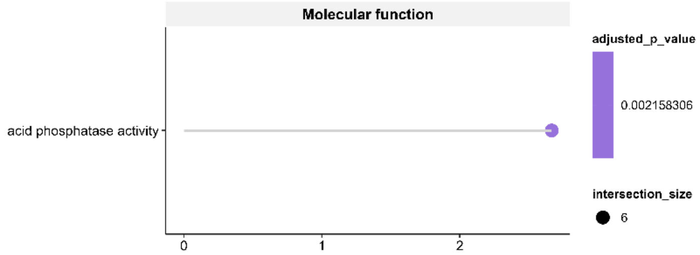

Figure S34. Gene ontology assignment of the root and leaf transcriptome of PI-665276, LM2, and CHEN-384 grown in the greenhouse under control and drought conditions. Significant transcripts of A) roots and B) leaves grown under drought and control conditions calculated by either Student's *t*-test or by Wilcoxon test based on their normal distribution. Cut-off criterion: *p*-value < 0.05, |$\log_2$ fold change| > 1. For GO analysis g:Profiler was used with a cut-off criterion of adjusted *p*-value < 0.05.
